# Limitations of nomogram models in predicting survival outcomes for glioma patients

**DOI:** 10.3389/fimmu.2025.1547506

**Published:** 2025-03-18

**Authors:** Jihao Xue, Hang Liu, Lu Jiang, Qijia Yin, Ligang Chen, Ming Wang

**Affiliations:** ^1^ Department of Neurosurgery, The Affiliated Hospital, Southwest Medical University, Luzhou, Sichuan, China; ^2^ Department of Urology or Nursing, Dazhou First People’s Hospital, Dazhou, Sichuan, China; ^3^ College of Nursing, Chongqing Medical University, Chongqing, Chongqing, China; ^4^ Academician (Expert) Workstation of Sichuan Province, The Affiliated Hospital, Southwest Medical University, Luzhou, China; ^5^ Neurological Diseases and Brain Function Laboratory, The Affiliated Hospital, Southwest Medical University, Luzhou, China

**Keywords:** glioma, nomogram, proportional hazards (PH) assumption, cox regression model, prediction

## Abstract

**Purpose:**

Glioma represents a prevalent and malignant tumor of the central nervous system (CNS), and it is essential to accurately predict the survival of glioma patients to optimize their subsequent treatment plans. This review outlines the most recent advancements and viewpoints regarding the application of nomograms in glioma prognosis research.

**Design:**

With an emphasis on the precision and external applicability of predictive models, we carried out a comprehensive review of the literature on the application of nomograms in glioma and provided a step-by-step guide for developing and evaluating nomograms.

**Results:**

A summary of thirty-nine articles was produced. The majority of nomogram-building research has used limited patient samples, disregarded the proportional hazards (PH) assumption in Cox regression models, and some of them have failed to incorporate external validation. Furthermore, the predictive capability of nomograms is influenced by the selection of incorporated risk factors. Overall, the current predictive accuracy of nomograms is moderately credible.

**Conclusion:**

The development and validation of nomogram models ought to adhere to a standardized set of criteria, thereby augmenting their worth in clinical decision-making and clinician-patient communication. Prior to the clinical application of a nomogram, it is imperative to thoroughly scrutinize its statistical foundation, rigorously evaluate its accuracy, and, whenever feasible, assess its external applicability utilizing multicenter databases.

## Introduction

Gliomas constitute a prevalent and heterogeneous class of primary tumors within the central nervous system (CNS), including glioblastoma (GBM) and low-grade glioma (LGG). About 75% of malignant primary brain tumors in adults are gliomas, which can diffusely penetrate the brain parenchyma (1, 2). Gliomas are neuroectoderm-derived tumors that arise from glial cells or their progenitor cells, which encompass a spectrum of histological subtypes including ependymomas, oligodendrogliomas, and astrocytomas, among others (3). In accordance with the World Health Organization (WHO) classification of tumors of the CNS published in 2021, GBM is categorized as a WHO grade 4 tumor, whereas gliomas of WHO grade 2 and 3 are categorized as LGG (4, 5). Gliomas, especially glioblastoma multiforme, have a high death rate and are strongly linked to neurological impairment. Their propensity for recurrence following surgical intervention presents a formidable challenge for neurosurgeons and neuro-oncologists (6). In order to help clinicians create individual treatment programs and make decisions about follow-up and imaging intervals, a useful and user-friendly predictive model that can provide reliable prognostic information for glioma patients is desperately needed.

The nomogram constitutes a statistical predictive model that amalgamates multiple risk factors to estimate individual survival probabilities. Given its capacity to produce numerical probabilities for the occurrence of clinical events and depict them via straightforward graphical displays, nomograms exhibit substantial advantages in comparison to the conventional TNM staging system for numerous cancers (7). In recent years, a multitude of nomogram models tailored specifically for gliomas have been developed and have garnered significant acceptance and popularity among neurosurgeons (8–12). However, it is crucial to acknowledge the inherent limitations of these studies that have produced nomogram models, which encompass the relatively small sample sizes incorporated, the existence of racial disparities, the absence of external validation datasets, inadequate incorporation of variables, and the non-compliance with the proportional hazards (PH) assumption. These limitations undoubtedly pose challenges in interpreting these predictive outcomes within a more extensive clinical setting.

In this review, we rigorously assessed the precision of the nomograms and the credibility of their predictive outcomes across the included studies, while also engaging in a thorough discussion of the aforementioned limitations and proposing potential avenues for optimization. Furthermore, we have delineated a systematic flowchart for the development of nomogram models aimed at predicting the prognosis of glioma patients.

## Materials and methods

### Literature identification

We started data analysis in November 2024 after conducting a thorough literature review in the PubMed, Web of Science, and Embase databases that included publications published between January 1, 2004, and October 30, 2024. For additional information on the search strategy and retrieval subject terms, please refer to **Supplementary Data Sheet 1**.

### Study selection and data collection

All search outcomes were exported into the reference management software (EndNote X9.3.2), with duplicates being eliminated J.X. (for all studies), H.L., Q.Y., L.C., and M.W. (each examined every quarter) independently evaluated the titles and abstracts according to the inclusion criteria. Uncertainty-filled abstracts were included for full-text evaluation. The full-text articles were examined by J.X. to verify whether they were included or not. J.X. checked all data for accuracy and summarized (1) study information (investigator, publication year, tumor type, dataset utilized, sample size, data source); (2) metadata of glioma patients (if available); (3) risk score formula.

### Data source

RNA-seq profiles and corresponding clinicopathological information for glioma, LGG, and GBM patients were downloaded from The Cancer Genome Atlas (TCGA) and Chinese Glioma Genome Atlas (CGGA) (mRNAseq_array_301, mRNAseq_325 and mRNA_693) database. In addition, gene expression and glioma patient survival data in the Gravendeel database were downloaded from GlioVis. Metadata of patients in the TCGA-LGG, ZN-LGG, and SU-LGG cohorts collected by Liu et al. was obtained as well. The detailed methodology of the study is presented in **Supplementary Data Sheet 1**.

### Establishment and evaluation of the nomogram model

Drawing upon the datasets and prognostic risk factors employed in the selected studies, we utilized the “rms” R package (https://cran.r-project.org/web/packages/rms/index.html) to develop respective nomogram models for predicting the survival outcomes of glioma patients. To ascertain the precision and dependability of these models, we generated calibration plots for each and assessed the PH assumption based on “survival” R package (https://www.rdocumentation.org/packages/survival/versions/3.1-8).

### Statistical analysis

R software (version 4.0.2) was adopted for all statistical analysis in this research, and *p* < 0.05 was regarded as statistically significant. To evaluate the genetic and clinicopathological features associated with OS (overall survival), univariate and multivariate Cox regression analysis models were constructed.

## Results

**Figure 1:**
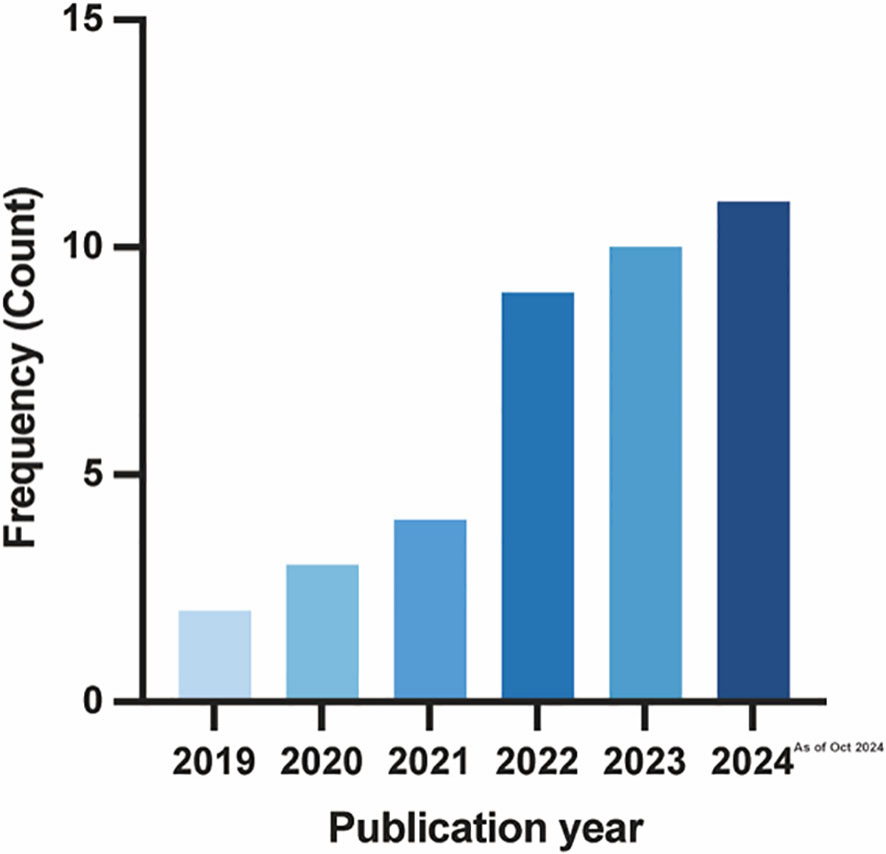
Flow diagram for screening research. Identified and included studies from the database searches (PubMed, Embase, and Web of Science).

**Figure 2:**
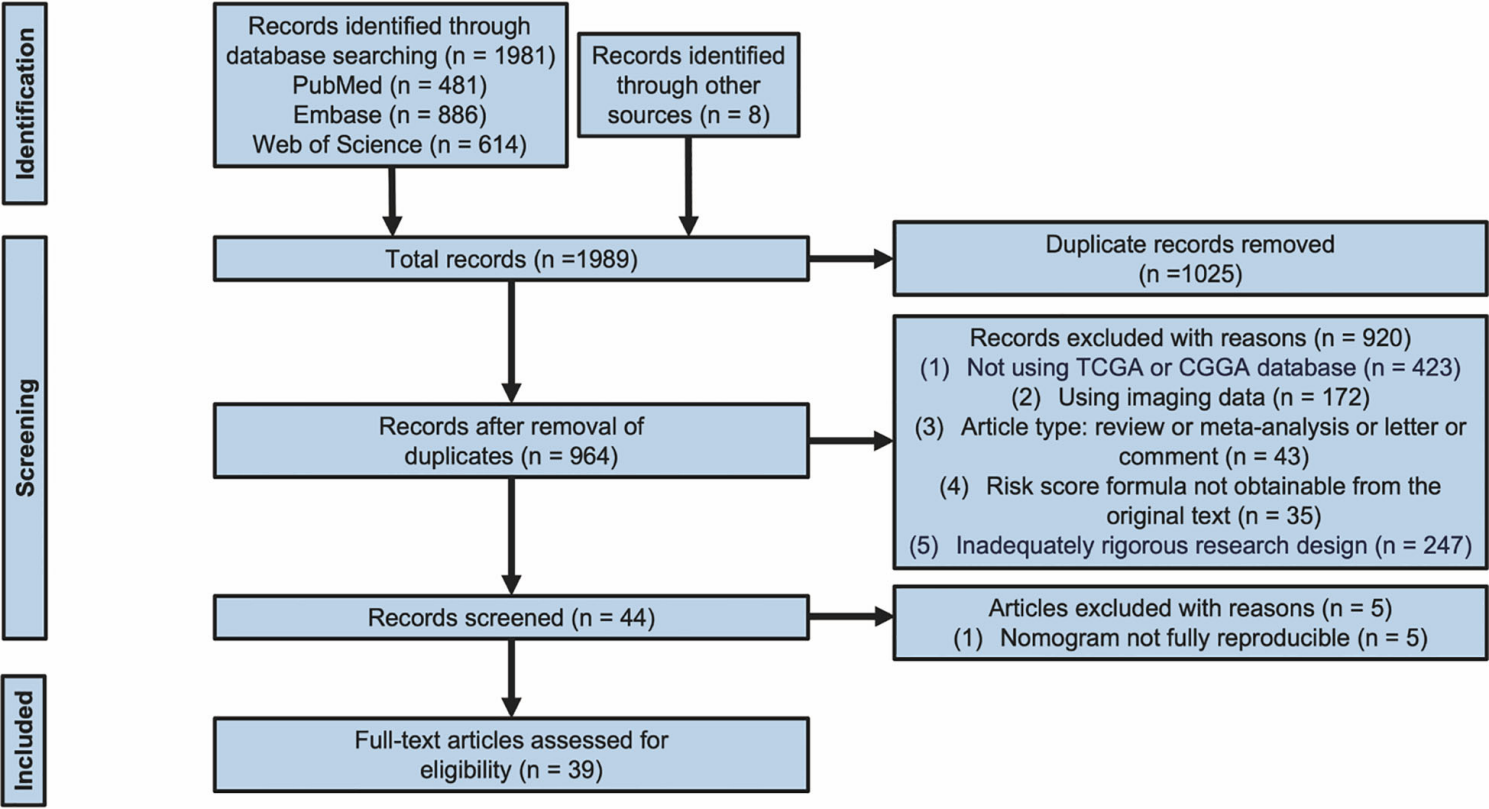
Publication year of the 39 included studies. Note that the number for 2024 is based on the articles published until 30th October 2024 and is therefore incomplete.

The flowchart provides a summary of the study screening procedure (**Figure 1**). Following a preliminary screening and deduplication of the 1,989 records found in the literature search conducted on October 30, 2024, we found 964 possibly pertinent citations. 44 studies were selected for full-text review after an additional screening of abstracts and titles. Among them, 5 studies (13–17) were disqualified because the authors either used alternative data formats or neglected to include selection criteria for survival and clinicopathological feature data, making it impossible to create nomogram models. Ultimately, 39 studies (8, 10, 11, 14, 17–51) met all the standards for inclusion. **Figure 2** displays the publication years of the included articles. In our review, over 75% of the articles were published in 2022 or later, with only a handful published prior to 2020. However, it must be taken into account that the total number of articles published in recent years has increased (52). This observation may indicate the recent developments in the techniques or methodologies for constructing prognostic nomograms and further underscores the escalating research interest in this particular domain.

### Study characteristics

The characteristics of the studies incorporated in this comprehensive review were summarized in **Tables 1**, **2**, with a special emphasis on the fact that they all hail from China. 28 studies (71.8%) (10, 11, 14, 18–26, 28, 29, 31, 32, 37, 39–46, 48, 49, 51) established nomogram models for predicting the survival rates of glioma patients, 8 studies (20.5%) (8, 17, 30, 33–36, 38) formulated nomogram models to forecast the survival outcomes of LGG patients, and 3 studies (7.7%) (27, 47, 50) constructed nomogram models for anticipating the survival probabilities of GBM patients. All of the investigations included multiple datasets, with 39 studies (representing 100% of the total) (8, 10, 11, 14, 17–51) incorporating the TCGA database and 33 studies (constituting 84.6% of the total) (10, 11, 14, 17–26, 28–38, 40, 42–45, 47–49, 51) utilizing the CGGA database. Nevertheless, it is worth highlighting that only 7 studies (17.9%) (18, 21, 28, 34, 43, 48, 51) employed external datasets for the validation of the nomogram, whereas 32 studies (82.1%) (8, 10, 11, 14, 17, 19, 20, 22–27, 29–33, 35–42, 44–47, 49, 50) did not utilize external datasets for the purpose of nomogram validation. The LASSO regression analysis was used in 18 studies (46.2%) (14, 17, 24, 25, 27, 34–38, 40–45, 47, 51) to determine the most relevant predictive feature genes. The risk score formula for glioma patients was then created using the gene expression levels as follows: *Risk score = Σ (Coefi × Exp)* The risk coefficient is denoted by Coefi in this formula, whereas the gene expression level is denoted by Exp.

**Table 1:** Researches on utilizing nomograms for prognosis prediction in glioma patients.

Investigator	Tumor type	Dataset utilized	Sample size: total cases	Data source	Risk score formula	External validation	C-index	*P*-value for the global test of the nomogram	Conformity to the PH assumption
*An et al. (18)	Glioma	TCGA (n=702) CGGA (n=693)	1395	http://cancergenome.nih.gov/ http://www.cgga.org.cn	NA	Yes	TCGA=0.861 (0.850-0.871) CGGA=0.749 (0.737-0.762)	TCGA=3.74e-05 CGGA= 0.0002	TCGA (No) CGGA (No)
Chang et al. (19)	Glioma	GSE4290 (n=176) GSE50161 (n=130) TCGA (n=592) CGGA325 (n=286) CGGA693 (n=429) Tissue Microarray (n=124)	1737	GEO, TCGA, CGGA database and tissue microarray	NA	No	CGGA325 = 0.808 (0.795-0.822)	CGGA325 = 0.0145	CGGA325 (No)
Yu et al. (20)	Glioma	TCGA (n=689) GTEx (n=1157) GSE4290 (n=84) CGGA325 (n=NA) CGGA693 (n=NA)	NA	https://xenabrowser.net/datapages/ GEO database http://www.cgga.org.cn/index.jsp	NA	No	TCGA=0.838 (0.827-0.849)	TCGA=1.27e-08	TCGA (No)
*Jiang et al. (21)	Glioma	TCGA (n=607) GTEx (n=NA) CGGA325+CGGA693 (n=961)	NA	https://portal.gdc.cancer.gov/ http://www.cgga.org.cn/	NA	Yes	TCGA=0.828 (0.816-0.840) CGGA=0.744 (0.735-0.754)	TCGA=0.0112 CGGA=8.45e-05	TCGA (No) CGGA (No)
Wang et al. (22)	Glioma	TCGA (n=607) GTEx (n=1152) CGGA (n=693) GSE50161 (n=63)	2515	TCGA, CGGA, GEO, GTEx database	NA	No	TCGA=0.857 (0.846-0.867) CGGA=0.749 (0.736-0.762)	TCGA=0.0002 CGGA=1.58e-06	TCGA (No) CGGA (No)
Song et al. (23)	Glioma	TCGA (n=523) GTEx (n=1152) CGGA (n=NA)	NA	https://www.cancer.gov/ccg/research/genome-sequencing/tcga https://www.genome.gov/Funded-Programs-Projects/Genotype-Tissue-Expression-Project http://www.cgga.org.cn	NA	No	TCGA=0.813 (0.791-0.836)	TCGA=0.0034	TCGA (No)
Chen et al. (25)	Glioma	TCGA (n=672) CGGA325+CGGA693 (n=1013)	1685	https://xenabrowser.net/ http://www.cgga.org.cn/	RS=0.4712 × *CALR* expression + 0.1171 × *CANX* expression + 0.2059 × *PSMB8* expression + 0.0198 × *PDIA3* expression + 0.0966 × *HLA-B* expression	No	TCGA=0.861 (0.848-0.873)	TCGA=0.0045	TCGA (No)
Han et al. (26)	Glioma	TCGA (n=667) CGGA693 (n=693) Rembrandt (n = 510)	1870	http://cancergenome.nih.gov http://www.cgga.org.cn http://rembrandt.nci.nih.gov	NA	No	CGGA693 = 0.751 (0.737-0.765)	CGGA693 = 6.83e-05	CGGA693 (No)
Zhang et al. (27)	GBM	TCGA-GBM (n=159) GSE83300 (n=50)	209	TCGA, GEO database	RS=0.2988 × *PDIA4* expression + 0.1705 × *PILRB* expression + 0.2448 × *DUSP6* expression + 0.3055 × *PTPRN* expression − 0.2095 × *CBLN1* expression	No	TCGA-GBM=0.862 (0.850-0.873)	TCGA-GBM=1.23e-06	TCGA-GBM (No)
*Wang et al. (28)	Glioma	TCGA (n=529) CGGA301 (n=301) CGGA325 (n=325) CGGA693 (n=693)	1848	TCGA, CGGA database	NA	Yes	TCGA-LGG=0.841 (0.821-0.860) CGGA301-LGG=0.699 (0.669-0.729) CGGA325-LGG=0.732 (0.709-0.755) CGGA693-LGG=0.702 (0.682-0.722)	TCGA-LGG=0.0002 CGGA301-LGG=0.0516 CGGA325-LGG=0.0035 CGGA693-LGG=0.0515	TCGA-LGG (No) CGGA301-LGG (Yes) CGGA325-LGG (No) CGGA693-LGG (Yes)
Zhang et al. (29)	Glioma	TCGA (n=529) TCGA-GBM (microarray, n= 489) CGGA301 (n=301) CGGA693 (n=693) GSE16011 (n=284) Gill (n=92) IVY-GBM (n=270) Rembrandt (n=580)	3238	http://cancergenome.nih.gov/ http://www.cgga.org.cn https://www.ncbi.nlm.nih.gov/geo/ Gill database http://glioblastoma.alleninstitute.org/ https://caintegrator.nci.nih.gov/rembrandt/	NA	No	TCGA=0.752 (0.739-0.765)	TCGA=3.35e-05	TCGA (No)
Ma et al. (30)	LGG	TCGA (n=616) CGGA325 (n=325) CGGA693 (n=693)	1634	http://cancergenome.nih.gov/ http://www.cgga.org.cn	NA	No	TCGA=0.827 (0.805-0.848) CGGA325 = 0.789 (0.767-0.811)	TCGA=0.0008 CGGA325 = 0.0034	TCGA (No) CGGA (No)
Ge et al. (31)	Glioma	TCGA (n=696) CGGA325 (n=325) CGGA693 (n=693) Nantong University Affiliated Hospital (n=183)	1897	TCGA, CGGA database, self-collected data	NA	No	TCGA=0.844 (0.832-0.855)	TCGA=1.81e-08	TCGA (No)
Peng et al. (32)	Glioma	TCGA (n=NA) GTEx (n=NA) CGGA (n=NA) Department of Neurosurgery of Wuhan Union Hospital (n=89)	NA	https://portal.gdc.cancer.gov/ https://gtexportal.org/ http://www.cgga.org.cn/ self-collected data	NA	No	TCGA=0.862 (0.852-0.873)	TCGA=3.6e-05	TCGA (No)
Zhi et al. (33)	LGG	TCGA (n=528 LGG + 168 GBM) CGGA325 (n=182 LGG + 139 GBM) CGGA693 (n=443 LGG + 249 GBM)	1709	http://cancergenome.nih.gov http://www.cgga.org.cn/	NA	No	TCGA-LGG-OS=0.858 (0.841-0.876) TCGA-LGG-DSS=0.872 (0.856-0.889)	TCGA-LGG-OS=3.58e-06 TCGA-LGG-DSS=2.06e-06	TCGA-LGG-OS (No) TCGA-LGG-DSS (No)
*Zhang et al. (34)	LGG	TCGA-LGG (n=529) GTEx (n=1152) CGGA301 (LGG, n=174) CGGA325 (LGG,n=182)	2037	https://portal.gdc.cancer.gov/ https://xenabrowser.net/datapages/ http://cgga.org.cn/	CRG score=0.164859 × *C21orf62* expression + 0.293187 × *DRAXIN* expression + 0.882099 × *ITPRID2* expression + 0.625577 × *MAP3K1* expression + 0.256801 × *MOXD1* expression	Yes	TCGA=0.846 (0.824-0.867) CGGA=0.709 (0.690-0.728)	TCGA=0.0003 CGGA=9.85e-05	TCGA (No) CGGA (No)
Li et al. (35)	LGG	TCGA-LGG (n=529) GTEx (n=1152) CGGA-microarray (LGG, n=174) CGGA-sequencing (LGG, n=443) REMBRANDT (LGG, n=162) GSE16011 (LGG, n=107)	2567	https://portal.gdc.cancer.gov https://xenabrowser.net/datapages/ https://www.ncbi.nlm.nih.gov/ http://www.betastasis.com/glioma/rembrandt/ https://www.ncbi.nlm.nih.gov/	SnG-Risk score=0.129695 × *AURKA* expression + 0.301414 × *CENPA* expression + 0.057793 × *LIMK1* expression − 0.753527 × *PATZ1* expression + 0.154034 × *TGFB1I1* expression + 0.362497 × *TLR3* expression	No	TCGA-LGG=0.826 (0.797-0.855)	TCGA-LGG=3.33e-05	TCGA-LGG (No)
Li et al. (36)	LGG	TCGA-LGG (n=529) CGGA-microarray (LGG, n=174) CGGA-sequencing (LGG, n=625) REMBRANDT (LGG, n=162)	1490	TCGA, CGGA, REMBRANDT database,	RS=0.21536 × *ABCC3* expression + 0.23117 × *HOXA4* expression + 0.06596 × *HOXC10* expression + 0.04934 × *NNMT* expression + 0.37831 × *SCNN1B* expression	No	TCGA-LGG=0.853 (0.832-0.875)	TCGA-LGG=0.0039	TCGA-LGG (No)
Wang et al. (37)	Glioma	TCGA (n=NA) CGGA301 (n=301) CGGA325 (n=325) CGGA693 (n= 668) GSE108474 (n=NA) GSE43378 (n=NA) GSE16011 (n=NA) GSE68838 (n=NA)	NA	TCGA, CGGA, GEO database	RS= 0.028 × *CXCL1* expression + 0.027 × *CXCL9* expression + 0.14 × *CXCL10* expression + 0.04 × *CXCL11* expression − 0.12 × *CXCL12* expression + 0.047 × *CXCL14* expression	No	TCGA=0.849 (0.836-0.862)	TCGA=0.0305	TCGA (No)
Zhu et al. (38)	LGG	TCGA-LGG (n=515) CGGA325 (LGG, n=186) CGGA693 (LGG, n=144)	845	https://portal.gdc.cancer.gov/ http://www.cgga.org.cn/	RS=0.3413 × *TNFRSF11B* expression + 0.1794 × *METTL7B* expression − 0.2905 × *SSTR2* expression + 0.3566 × *OXTR* expression + 0.2803 × *CDKN2C* expression + 0.1194 × *H19* expression	No	TCGA-LGG=0.817 (0.793-0.841)	TCGA-LGG=0.0230	TCGA-LGG (No)
Geng et al. (39)	Glioma	TCGA (n=670) GTEx (n=1152) First Hospital of Jilin University (n=NA)	NA	XENA, self-collected data	NA	No	TCGA-OS=0.822 (0.803-0.841) TCGA-PFI=0.732 (0.713-0.750)	TCGA-OS=0.0014 TCGA-PFI=0.3300	TCGA-OS (No) TCGA-PFI (Yes)
Yu et al. (40)	Glioma	TCGA (n=669) CGGA325 (n=NA) CGGA693 (n= NA)	NA	https://portal.gdc.cancer.gov/ http://www.cgga.org.cn	CDRS RS= 0.490 × *BDKRB2* expression + 0.522 × *RFFL* expression + 0.551 × *SHISA5* expression − 0.668 × *TRAF3* expression + 0.731 × *FANCD2* expression + 0.703 × *GNS* expression	No	TCGA=0.885 (0.872-0.898)	TCGA=0.0085	TCGA (No)
Zhou et al. (41)	Glioma	TCGA-LGG (n=510) TCGA-GBM (n=159) GSE184941 (n=69) GSE108474 (n=70)	808	R/ Bioconductor package TCGAbiolinks https://www.ncbi.nlm.nih.gov/geo/query/acc.cgi?acc=GSE184941 https://www.ncbi.nlm.nih.gov/geo/query/acc.cgi?acc=GSE108474	RS=0.086 × *HOXA7* expression + 0.242 × *WEE1* expression + 0.247 × *IGF2BP3* expression + 0.052 × *DUSP10* expression	No	TCGA-LGG=0.808 (0.783-0.832)	TCGA-LGG=0.0012	TCGA-LGG (No)
Wu et al. (42)	Glioma	TCGA (n=NA) GTEx (n=NA) CGGA325 (n=NA) CGGA693 (n= NA)	NA	https://portal.gdc.cancer.gov/ http://www.cgga.org.cn/	RS=0.1983 × *CREB5* expression + 0.2359 × *ATF1* expression + 0.1581 × *ATF7* expression + 0.4231 × *CREB1* expression + 0.1578 × *CREB3L1* expression + 0.6542 × *CREB3L2* expression − 0.4324 × *CREBBP* expression − 0.8175 × *EP300* expression − 0.0533 × *FOS* expression + 0.1922 × *FOSL2* expression + 0.1508 × *JUN* expression	No	TCGA=0.864 (0.852-0.876)	TCGA=0.0161	TCGA (No)
*Peng et al. (43)	Glioma	TCGA (n=597) GTEx (n=NA) CGGA325 (n=305) CGGA693 (n= 655)	NA	https://portal.gdc.cancer.gov/ http://www.cgga.org.cn/	GA-MSCRGPI= − 0.260 × *MCM7* expression + 0.285 × *CDK6* expression + 0.709 × *ORC1* expression − 0.153 × *CCL20* expression + 0.202 × *TNFRSF12A* expression − 0.293 × *POLA1* expression + 0.271 × *TRAF1* expression − 0.310 × *TIAM1* expression	Yes	TCGA=0.857 (0.844-0.869) CGGA325 = 0.740 (0.726-0.754) CGGA693 = 0.714 (0.702-0.727)	CGGA693 = 0.0029 CGGA325 = 0.0002 TCGA=0.2300	CGGA693 (No) CGGA325 (No) TCGA (Yes)
Wang et al. (44)	Glioma	TCGA (n=702) CGGA325 (n=325) GSE16011 (n=268) Rembrandt (n=454)	1749	http://cancergenome.nih.gov/ CGGA dataset http://www.ncbi.nlm.nih.gov/geo/query/acc.cgi?acc=GSE16011 https://www.ncbi.nlm.nih.gov/geo/query/acc.cgi?acc=GSE108474	RS=0.0173 × *TNFSF4* expression + 0.0341 × *CD70* expression + 0.0371 × *TNFSF14* expression + 0.0426 × *TNFRSF19* expression + 0.0467 × *NGFR* expression + 0.1234 × *TNFRSF11B* expression + 0.1599 × *TNFRSF14* expression + 0.2198 × *TNFRSF12A* expression	No	TCGA=0.867 (0.857-0.878)	TCGA=0.0456	TCGA (No)
Lin et al. (11)	Glioma	TCGA (n=690) CGGA (n=929) GSE16011 (n=276)	1895	http://cancergenome.nih.gov www.cgga.org.cn/ GEO database	NA	No	CGGA=0.791 (0.781-0.800)	CGGA=4.15e-09	CGGA (No)
Han et al. (10)	Glioma	TCGA (n=669) CGGA (n=325) Rembrandt (n=510) Gill [24] (n=93) Ivy dataset (n=269)	1866	http://cancergenome.nih.gov http://www.cgga.org.cn http://rembrandt.nci.nih.gov https://www.ncbi.nlm.nih.gov/geo/	NA	No	CGGA=0.735 (0.719-0.751)	CGGA=1.62e-06	CGGA (No)
Liu et al. (8)	LGG	TCGA-LGG (n=488) ZN-LGG (n=70) SU-LGG (n=37) TCGA-GBM (n=380) ZN-GBM (n=77)	1052	GDC portal, self-collected data	NA	No	TCGA-LGG=0.828 (0.807-0.848)	TCGA-LGG=5.88e-05	TCGA-LGG (No)

LGG, low-grade glioma; GBM, glioblastoma multiforme; NA, not available; RS, risk score; ZN, Zhongnan Hospital of Wuhan University; SU, Medical Center of Stanford University; PH, proportional hazards; OS, overall survival; DSS, disease specific survival; PFI, progress free interval; *, study showing a decline of more than 10.0% in the C-index.

These studies fail to meet the proportional hazards assumption.

**Table 2:** Researches on utilizing nomograms for prognosis prediction in glioma patients.

Investigator	Tumor type	Dataset utilized	Sample size: total cases	Data source	Risk score formula	External validation	C-index	P-value for the global test of the nomogram	Conformity to the PH assumption
He et al. (45)	Glioma	TCGA (n=691) CGGA693 (n=590)	1281	https://portal.gdc.cancer.gov/ http://www.cgga.org.cn/	RS=0.18 × *GINS2* expression + 0.17 × *EGR1* expression + 0.58 × *ECT2* expression	No	TCGA=0.853 (0.842-0.865)	TCGA=0.2832	TCGA (Yes)
Zeng et al. (24)	Glioma	TCGA (n=596) CGGA325 (n=312)	908	https://portal.gdc.cancer.gov/ http://www.cgga.org.cn/	RS=0.249 × *HLA-DQA2* expression + 0.179 × *HOXA3* expression + 0.227 × *SAA2* expression	No	TCGA=0.860 (0.849-0.872)	TCGA=0.2663	TCGA (Yes)
Zhang et al. (46)	Glioma	TCGA (n=706) GTEx (n=1152) GSE14805 (n=38)	1896	https://portal.gdc.cancer.gov/ https://gtexportal.org/ http://www.ncbi.nlm.nih.gov/projects/geo/	NA	No	TCGA=0.815 (0.796-0.833)	TCGA=0.0781	TCGA (Yes)
Zhao et al. (47)	GBM	TCGA-GBM (n=169) CGGA (GBM, n=249)	418	https://xena.ucsc.edu/ http://www.cgga.org.cn	RS=0.17 × *EN1* expression + 0.09 × *TUBB2A* expression + 0.14 × *HSPB1* expression + 0.19 × *LOXL1* expression + 0.09 × *RGS4* expression + 0.08 × *L1CAM* expression + 0.20 × *GPR143* expression	No	TCGA-GBM=0.716 (0.688-0.744)	TCGA-GBM=0.6113	TCGA-GBM (Yes)
Wang et al. (48)	Glioma	TCGA (n=598) CGGA301 (n=301) REMBRANDT (n=NA) Gravendeel (n=211)	NA	http://gliovis.bioinfo.cnio.es/ http://www.cgga.org.cn/	NA	Yes	CGGA301 = 0.739 (0.723-0.755) Gravendeel=0.733 (0.713-0.752) TCGA=0.859 (0.848-0.870)	CGGA301 = 0.0561 Gravendeel=0.4008 TCGA=0.0004	CGGA301 (Yes) Gravendeel (Yes) TCGA (No)
Song et al. (17)	LGG	TCGA-LGG (n=469) CGGA (LGG, n=405) CGGA-microarray (n=118) GSE16011 (n=88) GSE61374 (n=136)	1216	TCGA data portal, CGGA database, https://www.ncbi.nlm.nih.gov/geo/	RS=2.1627 × *IGFBP5* expression + 1.8334 × *CENPF* expression + 1.4131 × *CD101* expression + 1.3129 × *SIGLEC1* expression + 1.3071 × *TMPRSS3* expression + 0.8839 × *SIGLEC8* expression + 0.8605 × *BIRC5* expression + 0.8552 × *EMP1* expression + 0.4835 × *SPP1* expression + 0.4357 × *PDCD1LG2* expression + 0.3861 × *FABP5* expression + 0.2623 × *CD3*7 expression + 0.2018 × *CD300LF* expression + 0.0448 × *ADAMTS3* expression − 0.0003 × *PROK2* expression − 0.3417 × *CBX6* expression − 0.9706 × *GPR27* expression − 1.2091 × *CRYBB1* expression − 1.5871 × *ANKRD22* expression − 3.3937 × *HEY1* expression	No	TCGA-LGG=0.887 (0.869-0.905)	TCGA-LGG=0.0906	TCGA-LGG (Yes)
Dai et al. (49)	Glioma	TCGA (n=NA) GTEx (n=NA) CGGA (n=NA)	NA	https://portal.gdc.cancer.gov/repository http://www.cgga.org.cn/	NA	No	TCGA-OS=0.789 (0.775-0.803) TCGA-DSS=0.786 (0.771-0.801) TCGA-PFI=0.722 (0.707-0.737)	TCGA-OS=0.0691 TCGA-DSS=0.1676 TCGA-PFI=0.0215	TCGA-OS (Yes) TCGA-DSS (Yes) TCGA-PFI (No)
Xie et al. (50)	GBM	TCGA-GBM (n=168) GTEx (n=NA) GSE43289/GSE43378/GSE15824/GSE34152/GSE50161/GSE66354/GSE16011 (n=NA)	NA	https://portal.gdc.cancer.gov/ https://www.gtexportal.org/home/datasets	NA	No	TCGA-GBM=0.628 (0.599-0.656)	TCGA-GBM=0.5165	TCGA-GBM (Yes)
Zeng et al. (14)	Glioma	TCGA (n=699) CGGA (n=325)	1024	http://cancergenome.nih.gov http://www.cgga.org.cn	RS= − 0.143 × *ATL1* expression − 0.094 ×* GRIA3* expression + 0.171 × *HPX* expression − 0.305 × *IL17D* expression − 0.132 × *KLHDC1* expression − 0.096 × *NCAM2* expression − 0.124 × *TRIM67* expression	No	TCGA=0.856 (0.844-0.868)	TCGA=0.1571	TCGA (Yes)
*Wang et al. (51)	Glioma	TCGA (n=672) CGGA-microarray (n=263) CGGA325 (n=320) CGGA693 (n=693) GSE108474 (n=294)	2242	https://cancergenome.nih.gov/ http://www.cgga.org.cn/ https://www.ncbi.nlm.nih.gov/gds	RS=0.30145833 × *ARNTL* expression + 0.08807400 × *ARNTL2* expression + 0.14204612 × *BHLHE40* expression − 0.36049846 × *CRY2* expression − 0.06305725 × *CSNK1E* expression − 0.03387665 × *HLF* expression − 0.04254696 × *NR1D2* expression − 0.08047881 × *PER3* expression + 0.01191781 × *RORC* expression + 0.17275780 × *TIMELESS* expression	Yes	TCGA=0.863 (0.852-0.875) CGGA325 = 0.691 (0.671-0.710)	TCGA=0.3776 CGGA325 = 0.1789	TCGA (Yes) CGGA325 (Yes)

NA, not available; RS, risk score; OS, overall survival; DSS, disease specific survival; PFI, progress free interval; *, study showing a decline of more than 10.0% in the C-index.

These studies adhere to the proportional hazards assumption.

### Assessment of nomograms in included research

After independently screening the data utilized in each research in accordance with the specific criteria for data inclusion in each included study, we performed univariate and multivariate COX regression analysis for every investigation. Following that, we developed corresponding nomogram models to forecast the survival prospects of glioma patients based on the several independent prognostic variables used by these investigations (**Supplementary Figures 1A**-**39A**; **Supplementary Data Sheet 2**). Apart from that, we evaluated the accuracy and reliability of each nomogram. This encompassed generating calibration curves (**Supplementary Figures 1B**-**39B**; **Supplementary Data Sheet 2**), figuring out the concordance index (C-index) (**Tables 1**, **2**), performing rigorous PH assumption testing on the variables included in each model (**Supplementary Tables 1**-**39**; **Supplementary Data Sheet 3**), and confirming whether the model as a whole complied with the PH assumption. In these studies that incorporated external datasets, we also used the corresponding data to plot additional calibration curves for verification (**Supplementary Figures 1C**, **4C**, **10C-E**, **16C**, **25C-D**, **34C-D**, **39C**; **Supplementary Data Sheet 2**). Among all the studies analyzed by us, 31 studies (79.5%) (8, 14, 17–25, 27, 28, 30–46, 51) exhibited C-index values exceeding 0.8. Nevertheless, among the 7 studies that validated the predictive performance of their nomograms using external datasets, a notable 85.7% (6 studies) (18, 21, 28, 34, 43, 51) reported a decline of more than 10.0% in the C-index, suggesting a reduction in accuracy for prediction when the nomograms were tested against external data. Among the 29 studies (74.4%) (8, 10, 11, 18–23, 25–44), the *p*-values obtained from the global validation test of the nomogram were ≤ 0.05 (**Table 1**). Conversely, in 10 studies (25.6%) (14, 17, 24, 45–51), the *p*-values exceeded 0.05 for the global validation test (**Table 2**). In cases where the global test yields a *p*-value greater than 0.05, it is deemed that the multifactorial model fulfills the proportional hazards assumption.

### Developing a predictive survival model tailored for glioma patients

**Figure 3:**
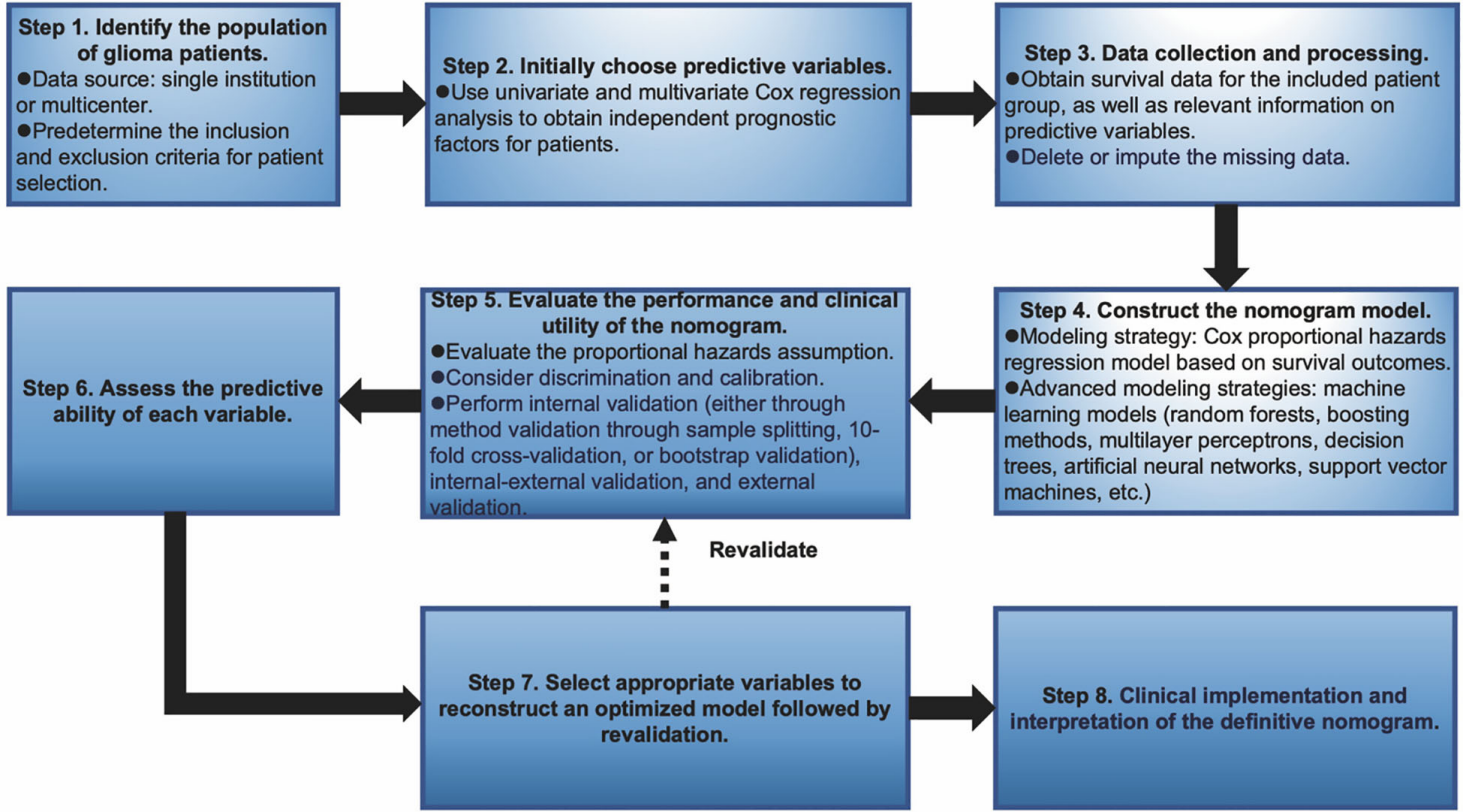
Graphical overview of proposed steps for developing and validating a predictive survival model tailored for glioma patients.

**Figure 4:**
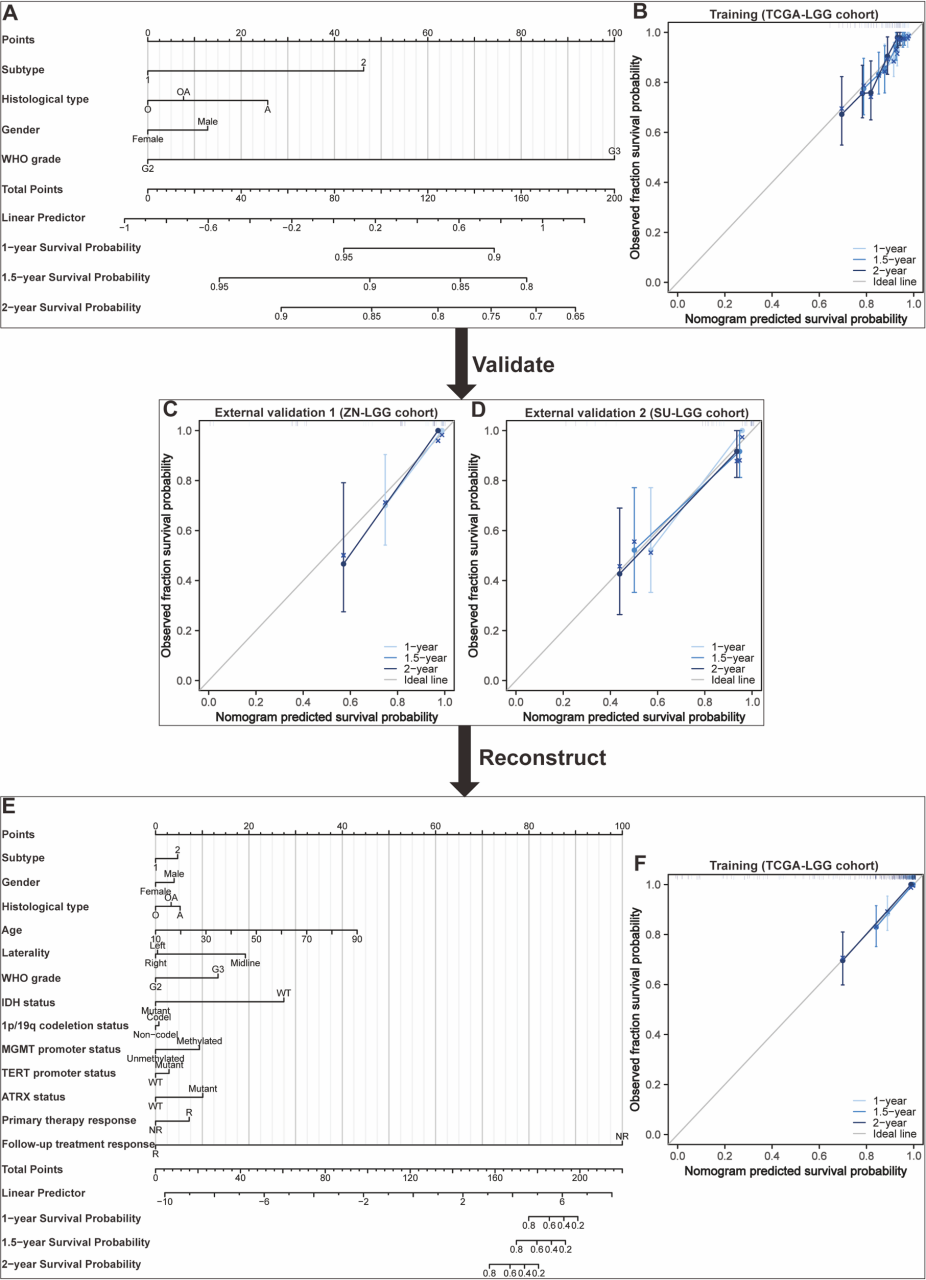
Development and validation of nomograms targeted at lower-grade glioma (LGG) as an illustrative example. **(A)** The first developed nomogram for predicting 1-, 1.5-, and 2-year overall survival (OS) based on the TCGA-LGG cohort. **(B)** Calibration plots showing the predicted probabilities of 1-, 1.5-, and 2-year OS by the first constructed model in the TCGA-LGG cohort compared to actual outcomes. **(C)** Calibration plots showing the predicted probabilities of 1-, 1.5-, and 2-year OS by the first constructed model in the ZN-LGG cohort compared to actual outcomes. **(D)** Calibration plots showing the predicted probabilities of 1-, 1.5-, and 2-year OS by the first constructed model in the SU-LGG cohort compared to actual outcomes. **(E)** The reconstructed nomogram for predicting 1-, 1.5-, and 2-year OS based on the TCGA-LGG cohort. **(F)** Calibration plots showing the predicted probabilities of 1-, 1.5-, and 2-year OS by the reconstructed model in the TCGA-LGG cohort compared to actual outcomes.

**Figure 5:**
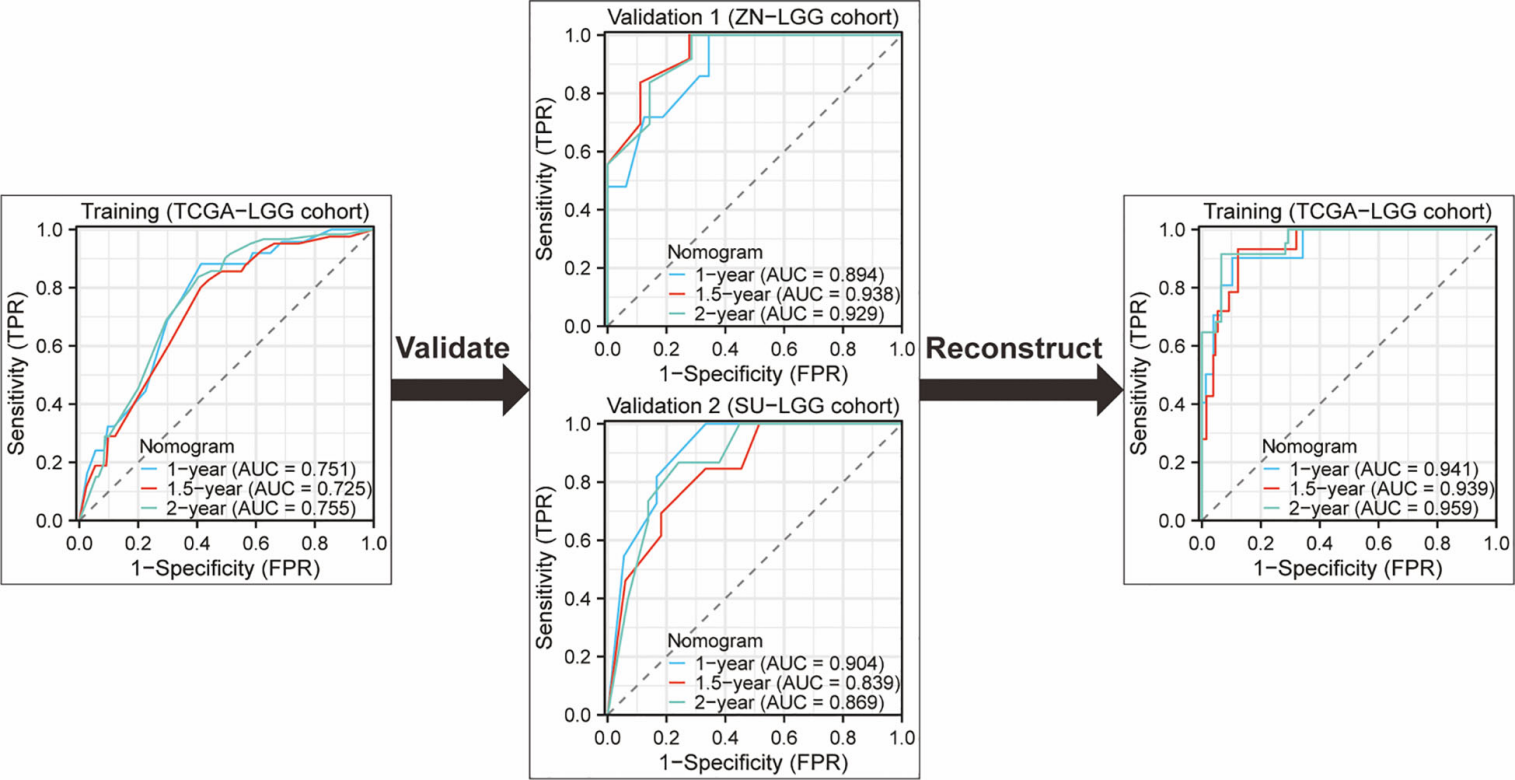
ROC curves and AUC for 1-Year, 1.5-year, and 2-year OS predictions using the original nomogram model in training set and external validation set. Additionally, ROC curves and AUC for 1-year, 1.5-year, and 2-year OS predictions using the optimized nomogram model.

Nomograms greatly increase the readability of predictive model results due to their ability to translate complex regression equations into visual graphics. This helps doctors create individualized treatment plans and increases the precision and efficacy of clinical decision-making processes. Consequently, we have delineated the sequence of independent steps entailed in developing nomograms for predicting glioma patients’ survival and suggested diverse verification methods to evaluate them. The fundamental procedures for establishing a nomogram specifically for glioma patients encompass identifying the patient cohort, initially screening predictive variables, collecting and processing data, constructing the nomogram model, assessing the clinical applicability and predictive accuracy of the survival outcome prediction model, evaluating the predictive capability of individual variables, selecting appropriate variables to reconstruct an optimized model followed by revalidation, and ultimately, clinical implementation and interpretation of the definitive nomogram (**Figure 3**). To provide a concrete illustration of these steps, we utilized datasets acquired from ref. 8 to develop a nomogram as an example (**Supplementary Tables 40**-**42**; **Supplementary Data Sheet 3**). After sample screening, the prognostic factors were determined by Cox regression analysis. Utilizing the TCGA-LGG cohort as the training dataset, we initially selected patient subtype, histological type, gender, and WHO grade to construct a nomogram for predicting 1-, 1.5-, and 2-year OS in patients with LGG (**Figure 4A**). This model adheres to the PH assumption not only within the training set but also in the external validation sets, namely the ZN-LGG cohort (external validation set 1) and the SU-LGG cohort (external validation set 2) (**Supplementary Table 43**; **Supplementary Data Sheet 3**). The predictive capability of the nomogram is robust, evidenced by a C-index of 0.705 upon validation within the training dataset (**Figure 4B**). Moreover, this predictive performance has been further corroborated through calibration analysis conducted on external validation sets 1 and 2, yielding C-indices of 0.894 and 0.831, respectively (**Figures 4C, D**). Subsequently, we selected appropriate molecular and clinical variables again to further refine and optimize the nomogram model (**Figure 4E**). The PH hypothesis test confirmed that the refined model adhered to the necessary assumptions (**Supplementary Table 44**; **Supplementary Data Sheet 3**). Furthermore, we validated that this optimized model exhibited superior predictive power than the original model, as shown by a higher C-index of 0.886 as opposed to 0.705 (**Figure 4F**). In addition, the original model demonstrated the following predicted area under the receiver operating characteristic (ROC) curves (AUC) values for the 1-year, 1.5-year, and 2-year survival rates within the training set: 0.751, 0.725, and 0.755, respectively. In the external validation set 1, the predicted AUC values for the same survival rates were 0.894, 0.938, and 0.929, respectively. Similarly, in the external validation set 2, the predicted AUC values were 0.904, 0.839, and 0.869 for the 1-year, 1.5-year, and 2-year survival rates, respectively. In contrast, the optimized model exhibited predicted AUC values of 0.941, 0.939, and 0.959 for the 1-year, 1.5-year, and 2-year survival rates, respectively (**Figure 5**). Due to the limited sample size included, the example presented in this study serves merely as a point of reference, and additional external validation is needed to continue to improve and optimize the model.

## Discussion

Numerous studies have been dedicated to developing prognostic assessment models for glioma patients, which not only reflect the high priority placed on patient prognosis evaluation but also provide invaluable guidance for clinical practice. Traditional assessment methodologies, exemplified by the Karnofsky Performance Status (KPS) scale for glioma patients, integrate various factors, including the patient’s age, general well-being, neurological status, and radiological findings, to comprehensively assess their overall health condition, thereby facilitating the formulation of tailored treatment regimens (53). However, as research progresses, various new prognostic assessment tools have continually emerged, with nomograms being a particularly noteworthy one (54). Through the integration of diverse clinical and molecular factors, nomograms are designed to enhance the precision of survival probability predictions for patients, ultimately aiding clinicians in tailoring individualized treatment regimens. For example, nomograms can be used to assess the efficacy of particular therapeutic interventions, such as radiotherapy and chemotherapy, and to make reliable predictions regarding the survival outcomes of glioma patients undergoing specific treatments (10, 11, 26, 29).

### Limited data and lack of external validation

Despite demonstrating substantial potential in predicting the survival outcomes of glioma patients and furnishing clinicians with an intuitive and relatively straightforward instrument for assessing patient survival probabilities, nomograms still possess a range of common limitations that must be meticulously addressed prior to their broader adoption in clinical practice. Firstly, a prevalent issue observed in current research on the development and validation of nomogram models is the inclusion of relatively limited data, which inherently constrains the predictive accuracy and generalization capacity of these models (55). As a predictive tool grounded in statistical principles, the efficacy of nomograms heavily relies on the abundance and diversity of the training data. In instances where data quantity is inadequate, the model may struggle to adequately capture the intricate patterns and underlying correlations within the dataset, consequently leading to instability and biases in the predictive outcomes. Transfer learning has garnered significant attention in the application of small-sample problems. By transferring model knowledge trained on large datasets to small sample datasets, transfer learning can significantly enhance the model’s generalization ability and accuracy (56). Secondly, the construction of a nomogram is typically based on specific research cohorts and datasets, which may not comprehensively represent the actual circumstances of the entire glioma patient population. For instance, certain studies may primarily rely on data derived from European and American populations (54). However, glioma, being a disease with notable racial and geographical disparities, is likely influenced by a multitude of factors, including genetic background, environmental conditions, lifestyle habits, and others, in terms of its pathogenesis, pathological types, treatment responses, and prognosis (57). The nomogram created based on particular population data and applied directly to other ethnic/racial groups without adequate adjustment or validation may result in lower prediction accuracy and even misleading clinical decisions because it fails to take into account the differences in incidence and survival rates among various ethnic groups (58). Furthermore, the majority of studies included in this review did not employ external validation cohorts to further evaluate the predictive performance of the nomogram models after their construction. External validation constitutes a crucial step in ensuring the reliability and stability of the models, as it aids researchers in determining the applicability of the models across diverse patient populations (59). Additionally, external validation serves as an effective means of identifying potential issues with the models, such as overfitting, where the model performs exceptionally well on the training data but exhibits poor generalization capabilities on new data, or underfitting, where the model fails to adequately capture crucial information within the data, leading to suboptimal prediction outcomes (60, 61). Consequently, the absence of external validation not only undermines the credibility of the models but also hinders the broad application of the research findings in clinical practice. Additionally, in seven studies that employed external datasets to assess the predictive performance of their nomogram models, six studies showed a decline of over 10.0% in the C-index compared to the training set. This decrement may be attributed to potential discrepancies in data quality, variations in sample size, inadequate feature selection, or models being either overly intricate or simplistic between the external datasets and the training set (62). To address these challenges, future studies ought to endeavor to gather more extensive and representative multicenter datasets for the development of nomogram models. Simultaneously, upon completion of model construction, a rigorous validation process employing independent external cohorts should be adhered to, in accordance with standardized protocols, to thoroughly assess the predictive performance of the model. This validation should encompass not only the accuracy of prognostic predictions for patients but also the stability and applicability of the model across diverse clinical contexts.

### Selection of variables

Although the nomogram model’s accuracy in forecasting is now considered to be moderately trustworthy, it has not yet achieved a high level of precision. This may be attributed to factors such as the inherent limitations of the model and the significant clinical heterogeneity observed among glioma patients (63). In particular, the existing nomogram models lack comprehensive integration of crucial factors influencing the prognosis of glioma patients, such as the Karnofsky performance status (KPS), specific copy number variations (CNV) like cyclin-dependent kinase inhibitor 2A/B (CDKN2A/B) deletion, mutation of the telomerase reverse transcriptase (TERT) promoter, regulator of telomere elongation helicase 1, and pivotal molecular markers like the codeletion of chromosome arms 1p and 19q (1p/19q), methylation status of the promoter region of the gene O (6)-methylguanine-DNA methyltransferase (MGMT), and mutation of isocitrate dehydrogenase (IDH) enzyme (64–67). Moreover, the incidence and progression of glioma are intimately associated with the immune microenvironment, necessitating the inclusion of microenvironment- and immunotherapy-related variables within nomogram models (68). Nevertheless, the bulk of research has not sufficiently considered or incorporated these important elements, which undoubtedly impedes the further enhancement of the predictive efficacy of the model. Hence, subsequent studies can selectively incorporate significant clinical pathological features, radiomic features, surgical resection range, CNV, single-cell sequence features, immune microenvironment features like immune cell infiltration, immunotherapy-related variables like PD-L1 expression, and molecular features like MGMT methylation status into a nomogram for predicting the survival of glioma patients.

### Data processing

The creation of nomograms is constrained by the quality and integrity of the data. In actual clinical settings, the input data necessary for the model may be incomplete or inaccurate due to a variety of factors, including inadequate documentation of patient information, data loss, or the presence of outliers. Multiple imputation is a commonly used method for handling missing data, but the imputed values may inherently possess a certain degree of inaccuracy, owing to the intrinsic characteristics of the original data and other imputation-related factors (69). In addition, Zhang et al. merged two datasets from distinct sources, namely the TCGA-GBM and GSE83300 cohorts, upon which they developed a nomogram (27). Completely eliminating biases across varied datasets is still a difficult task, even with their best efforts to perform background adjustment and quantitative normalization on the original files, followed by the use of the “Combat” algorithm to reduce batch effects (70). Notably, when datasets originate from different experimental platforms or techniques, inherent discrepancies among the data may persist, potentially leading to misleading biological interpretations of the consolidated dataset. Furthermore, in cases where batch effects within the dataset are excessively intricate or unrecognized confounding factors are present, the “Combat” algorithm may fall short in fully eradicating these effects, thereby compromising the quality of the merged data (71). Given the above challenges, future research should give more meticulous consideration to strategies for data integration during the design and execution phases.

### Cox proportional hazards model

From the perspective of model construction, the nomogram utilized for predicting patient survival outcomes primarily relies on both univariate and multivariate Cox regression analysis which can screen out independent risk factors (72). The Cox regression model postulates that the hazard ratio remains invariant across the entire follow-up duration, implying a constant covariate effect over time (73). However, multiple factors could cause nonproportionality of hazards to emerge frequently in practice (74). Failure to adhere to the proportional hazards assumption can introduce inaccurate prediction results and distorted statistical conclusions derived from the model (75, 76). Besides, the model faces difficulties in dealing with time-dependent covariates and nonlinear relationships between variables. Accordingly, prior to the application of a nomogram, the PH assumption on the relationship between covariates and outcomes should be validated by future researchers using Schoenfeld residuals or other approaches. Researchers ought to apply other alternative approaches like the Cox model with time-varying effects, the piecewise hazards model, and the accelerated failure time (AFT) model rather than the conventional Cox proportional hazards model when the PH assumption is not satisfied (74, 77). For example, Bayonas et al. used the accelerated failure time model to predict the progression-free survival of patients with advanced, well-differentiated neuroendocrine tumors who received somatostatin analog therapy (78). Similarly, Reckamp et al. conducted a study on the association between germline pathogenic variants and the age at diagnosis of lung adenocarcinoma, employing an AFT model (79).

### Appropriate assessment

In some studies, it can be observed that there is often a certain degree of deviation between the calibration curves of the nomogram and the ideal line (typically represented as a diagonal), implying that the predictive results of the nomogram may not align with the actual outcomes when predicting individual risks (19, 22, 23, 27, 31, 33, 41, 49). Additionally, despite exhibiting satisfactory fitting performance within the training cohort, the calibration curves experience a notable decline in both fitting accuracy and the C-index within the validation cohort (34). The augmentation of such deviation may originate from multiple facets, encompassing but not confined to sample selection bias, model overfitting, inappropriate selection of feature variables, and inaccuracies during the data preprocessing phase. In practical contexts, complex interactions and associations frequently exist among risk factors. While nomograms can simplify these factors into a single numerical value or score, they may inadvertently overlook crucial relationships among the variables involved. The utilization of nomograms for survival prediction in glioma patients necessitates consideration of individual differences and disease progression (80, 81). Clinical manifestations and prognosis in glioma patients may diverge significantly across individuals, which nomograms may inadequately capture. Therefore, when applying the model to specific patients, it is necessary to make comprehensive judgments and adjustments based on the actual situation and changes in the patient’s condition.

### Future prospects

Given the importance of predicting the survival outcomes of glioma patients in clinical practice, this article offers step-by-step guidelines to assist researchers in developing and evaluating clinical predictive nomogram models (82). With the rapid development of big data and machine learning technology, researchers can also explore the use of these advanced technologies to optimize the construction and validation process of nomogram models. For example, ensemble learning and deep learning can be employed to more efficiently handle large-scale datasets, thereby enhancing the predictive accuracy and robustness of the models (83). Meanwhile, improvements to the models can be achieved through techniques such as feature engineering, feature extraction and selection, and algorithm optimization. For instance, feature engineering can involve the generation of new features through the transformation or combination of original features, ultimately bolstering the expressive capacity of the model (84). Additionally, algorithm optimization can be carried out by adjusting model parameters or incorporating novel algorithms to further elevate model performance. Researchers can also adopt more advanced modeling strategies, including random forests, boosting methods, multilayer perceptrons, decision trees, artificial neural networks, support vector machines, and others (85). By integrating multi-dimensional information such as imaging, genomics, and clinical data, a more comprehensive and accurate prognostic prediction nomogram model can be constructed. To further explain the external validity and potential differences among different populations, external validation of the model is an indispensable step. Furthermore, GBM, being the highest-grade glioma, typically exhibits a more rapid growth rate and a poorer prognosis, whereas LGG displays relatively slower growth and a more favorable prognosis. The disparity in such biological behaviors necessitates the consideration of developing distinct prognostic models tailored to these two patient populations. This endeavor would facilitate more precise predictions of glioma patient prognosis and provide a scientific rationale for personalizing treatment regimens.

In conclusion, we have conducted an assessment of the nomograms included in our study. When the model significantly violates the proportional hazards assumption, meticulous attention must be devoted to adopting suitable analytical strategies. It is of crucial importance to ascertain the type of non-proportional hazards and to choose the most suitable analytical technique tailored to that specific context. Hence, in the face of the challenge posed by models violating the proportional hazards assumption, it is imperative for us to continually delve into and experiment with novel analytical strategies and techniques, thereby ensuring the precision and reliability of predictive outcomes. This endeavor not only enhances the scientific rigor and efficacy of clinical decision-making but also offers valuable insights and guidance for future research endeavors. A step-by-step guide for developing and validating nomograms offers potential clinical value.

## Data Availability

The original contributions presented in the study are included in the article/**Supplementary Material**. Further inquiries can be directed to the corresponding authors.

## References

[1] Ostrom QT Gittleman H Liao P Vecchione-Koval T Wolinsky Y Kruchko C . CBTRUS Statistical Report: Primary brain and other central nervous system tumors diagnosed in the United States in 2010-2014. Neuro Oncol. (2017) 19:v1–v88. doi: 10.1093/neuonc/nox158 29117289 PMC5693142

[2] Schaff LR Mellinghoff IK . Glioblastoma and other primary brain Malignancies in adults: A review. Jama. (2023) 329:574–87. doi: 10.1001/jama.2023.0023 PMC11445779 36809318

[3] van den Bent MJ Geurts M French PJ Smits M Capper D Bromberg JE . Primary brain tumours in adults. Lancet. (2023) 402:1564–79. doi: 10.1016/S0140-6736(23)01054-1 37738997

[4] Louis DN Perry A Wesseling P Brat DJ Cree IA Figarella-Branger D . The 2021 WHO classification of tumors of the central nervous system: a summary. Neuro Oncol. (2021) 23:1231–51. doi: 10.1093/neuonc/noab106 PMC8328013 34185076

[5] Yao L Hatami M Ma W Skutella T . Vaccine-based immunotherapy and related preclinical models for glioma. Trends Mol Med. (2024) 30:965–81. doi: 10.1016/j.molmed.2024.06.009 39013724

[6] Liu Y Zhou F Ali H Lathia JD Chen P . Immunotherapy for glioblastoma: current state, challenges, and future perspectives. Cell Mol Immunol. (2024) 21:1354–75. doi: 10.1038/s41423-024-01226-x PMC11607068 39406966

[7] Sternberg CN . Are nomograms better than currently available stage groupings for bladder cancer? J Clin Oncol. (2006) 24:3819–20. doi: 10.1200/JCO.2006.07.1290 16864852

[8] Liu XP Jin X Seyed Ahmadian S Yang X Tian SF Cai YX . Clinical significance and molecular annotation of cellular morphometric subtypes in lower-grade gliomas discovered by machine learning. Neuro Oncol. (2023) 25:68–81. doi: 10.1093/neuonc/noac154 35716369 PMC9825346

[9] Han M-Z Huang B Ni S-L Wang J Li X-G Bjerkvig R . A validated prognostic nomogram for patients with newly diagnosed lower-grade gliomas in a large-scale Asian cohort. Neuro-Oncology. (2020) 22:729–31. doi: 10.1093/neuonc/noaa027 PMC7229241 32025722

[10] Han M-Z Wang S Zhao W-B Ni SL Yang N Kong Y . Immune checkpoint molecule herpes virus entry mediator is overexpressed and associated with poor prognosis in human glioblastoma. Ebiomedicine. (2019) 43:159–70. doi: 10.1016/j.ebiom.2019.04.002 PMC6557785 30987862

[11] Lin W Sun Y Qiu X Huang Q Kong L Lu JJ . VMP1, a novel prognostic biomarker, contributes to glioma development by regulating autophagy. J Neuroinflammation. (2021) 18:165. doi: 10.1186/s12974-021-02213-z 34311746 PMC8311950

[12] Gorlia T van den Bent MJ Hegi ME Mirimanoff RO Weller M Cairncross JG . Nomograms for predicting survival of patients with newly diagnosed glioblastoma: prognostic factor analysis of EORTC and NCIC trial 26981-22981/CE.3. Lancet Oncol. (2008) 9:29–38. doi: 10.1016/S1470-2045(07)70384-4 18082451

[13] Li J Zhang S Chen S Yuan YB Zuo MR Li TF . Lipid metabolism-related gene signature predicts prognosis and depicts tumor microenvironment immune landscape in gliomas. Front Immunol. (2023) 14:1021678. doi: 10.3389/fimmu.2023.1021678 36860853 PMC9968762

[14] Zeng F Wang K Liu X Zhao Z . Comprehensive profiling identifies a novel signature with robust predictive value and reveals the potential drug resistance mechanism in glioma. Cell Commun Signal. (2020) 18:2. doi: 10.1186/s12964-019-0492-6 31907037 PMC6943920

[15] Yuan YS Jin X Chen L Liao JM Zhang Y Yu KW . A novel model based on necroptosis-related genes for predicting immune status and prognosis in glioma. Front Immunol. (2022) 13:1027794. doi: 10.3389/fimmu.2022.1027794 36389690 PMC9640834

[16] Chen J Shen S Li Y Fan J Xiong S Xu J . APOLLO: An accurate and independently validated prediction model of lower-grade gliomas overall survival and a comparative study of model performance. EBioMedicine. (2022) 79:104007. doi: 10.1016/j.ebiom.2022.104007 35436725 PMC9035655

[17] Song LR Weng JC Li CB Huo X Li H Hao S . Prognostic and predictive value of an immune infiltration signature in diffuse lower-grade gliomas. JCI Insight. (2020) 5. doi: 10.1172/jci.insight.133811 PMC7205440 32229719

[18] An W Ren C Yuan L Qiu Z Wang P Cheng Y . High expression of SIGLEC7 may promote M2-type macrophage polarization leading to adverse prognosis in glioma patients. Front Immunol. (2024) 15:1411072. doi: 10.3389/fimmu.2024.1411072 39211050 PMC11357930

[19] Chang X Pan J Zhao R Yan T Wang X Guo C . DDOST correlated with Malignancies and immune microenvironment in gliomas. Front Immunol. (2022) 13:917014. doi: 10.3389/fimmu.2022.917014 35812432 PMC9260604

[20] Yu K Ji Y Liu M Shen F Xiong X Gu L . High expression of CKS2 predicts adverse outcomes: A potential therapeutic target for glioma. Front Immunol. (2022) 13. doi: 10.3389/fimmu.2022.881453 PMC9160311 35663965

[21] Jiang Y Ji Q Long X Wang P Tu Z Zhang X . CLCF1 is a novel potential immune-related target with predictive value for prognosis and immunotherapy response in glioma. Front Immunol. (2022) 13. doi: 10.3389/fimmu.2022.810832 PMC8898905 35265072

[22] Wang J Li X Wang K Li K Gao Y Xu J . CLEC7A regulates M2 macrophages to suppress the immune microenvironment and implies poorer prognosis of glioma. Front Immunol. (2024) 15. doi: 10.3389/fimmu.2024.1361351 PMC11153702 38846954

[23] Song D Yang Q Li L Wei Y Zhang C Du H . Novel prognostic biomarker TBC1D1 is associated with immunotherapy resistance in gliomas. Front Immunol. (2024) 15. doi: 10.3389/fimmu.2024.1372113 PMC10961388 38529286

[24] Zeng Z Hu C Ruan W Zhang J Lei S Yang Y . A specific immune signature for predicting the prognosis of glioma patients with IDH1-mutation and guiding immune checkpoint blockade therapy. Front Immunol. (2022) 13. doi: 10.3389/fimmu.2022.1001381 PMC9500319 36159801

[25] Chen R Zhang H Wu W Li S Wang Z Dai Z . Antigen presentation machinery signature-derived CALR mediates migration, polarization of macrophages in glioma and predicts immunotherapy response. Front Immunol. (2022) 13:833792. doi: 10.3389/fimmu.2022.833792 35418980 PMC8995475

[26] Han M Sun Y Zhao W Xiang G Wang X Jiang Z . Comprehensive characterization of TNFSF14/LIGHT with implications in prognosis and immunotherapy of human gliomas. Front Immunol. (2022) 13:1025286. doi: 10.3389/fimmu.2022.1025286 36341396 PMC9632349

[27] Zhang B Xie L Liu J Liu A He M . Construction and validation of a cuproptosis-related prognostic model for glioblastoma. Front Immunol. (2023) 14:1082974. doi: 10.3389/fimmu.2023.1082974 36814929 PMC9939522

[28] Wang X Huang Y Li S Zhang H . Integrated machine learning methods identify FNDC3B as a potential prognostic biomarker and correlated with immune infiltrates in glioma. Front Immunol. (2022) 13:1027154. doi: 10.3389/fimmu.2022.1027154 36275754 PMC9582524

[29] Zhang L Qu X Xu Y . Molecular and immunological features of TREM1 and its emergence as a prognostic indicator in glioma. Front Immunol. (2024) 15:1324010. doi: 10.3389/fimmu.2024.1324010 38370418 PMC10869492

[30] Ma W Zhang K Bao Z Jiang T Zhang Y . SAMD9 is relating with M2 macrophage and remarkable Malignancy characters in low-grade glioma. Front Immunol. (2021) 12:659659. doi: 10.3389/fimmu.2021.659659 33936093 PMC8085496

[31] Ge X Xu M Cheng T Hu N Sun P Lu B . TP53I13 promotes metastasis in glioma via macrophages, neutrophils, and fibroblasts and is a potential prognostic biomarker. Front Immunol. (2022) 13:974346. doi: 10.3389/fimmu.2022.974346 36275718 PMC9585303

[32] Peng Z Wang J Tong S Wu Y Yi D Xiang W . Phosducin-like 3 is a novel prognostic and onco-immunological biomarker in glioma: A multi-omics analysis with experimental verification. Front Immunol. (2023) 14:1128151. doi: 10.3389/fimmu.2023.1128151 37006287 PMC10050339

[33] Zhi W Wang Y Jiang C Gong Y Chen Q Mao X . PLEKHA4 is a novel prognostic biomarker that reshapes the tumor microenvironment in lower-grade glioma. Front Immunol. (2023) 14:1128244. doi: 10.3389/fimmu.2023.1128244 37818357 PMC10560889

[34] Zhang H Li X Li Y Chen B Zong Z Shen L . An immune-related signature for predicting the prognosis of lower-grade gliomas. Front Immunol. (2020) 11:603341. doi: 10.3389/fimmu.2020.603341 33363544 PMC7753319

[35] Li J Wang J Liu D Tao C Zhao J Wang W . Establishment and validation of a novel prognostic model for lower-grade glioma based on senescence-related genes. Front Immunol. (2022) 13:1018942. doi: 10.3389/fimmu.2022.1018942 36341390 PMC9633681

[36] Li J Wang S Chi X He Q Tao C Ding Y . Identification of heterogeneous subtypes and a prognostic model for gliomas based on mitochondrial dysfunction and oxidative stress-related genes. Front Immunol. (2023) 14:1183475. doi: 10.3389/fimmu.2023.1183475 37334354 PMC10272431

[37] Wang Z Liu Y Mo Y Zhang H Dai Z Zhang X . The CXCL family contributes to immunosuppressive microenvironment in gliomas and assists in gliomas chemotherapy. Front Immunol. (2021) 12:731751. doi: 10.3389/fimmu.2021.731751 34603309 PMC8482424

[38] Zhu W Chen Z Fu M Li Q Chen X Li X . Cuprotosis clusters predict prognosis and immunotherapy response in low-grade glioma. Apoptosis. (2024) 29:169–90. doi: 10.1007/s10495-023-01880-y PMC10830610 37713112

[39] Geng R Zhao Y Xu W Ma X Jiang Y Han X . SIRPB1 regulates inflammatory factor expression in the glioma microenvironment via SYK: functional and bioinformatics insights. J Transl Med. (2024) 22:338. doi: 10.1186/s12967-024-05149-z 38594692 PMC11003053

[40] Yu M Huo D Yu K Zhou K Xu F Meng Q . Crosstalk of different cell-death patterns predicts prognosis and drug sensitivity in glioma. Comput Biol Med. (2024) 175:108532. doi: 10.1016/j.compbiomed.2024.108532 38703547

[41] Zhou Q Wang Y Xin C Wei X Yao Y Xia L . Identification of telomere-associated gene signatures to predict prognosis and drug sensitivity in glioma. Comput Biol Med. (2024) 168:107750. doi: 10.1016/j.compbiomed.2023.107750 38029531

[42] Wu Z Wang X Wu H Du S Wang Z Xie S . Identification of CREB5 as a prognostic and immunotherapeutic biomarker in glioma through multi-omics pan-cancer analysis. Comput Biol Med. (2024) 173:108307. doi: 10.1016/j.compbiomed.2024.108307 38547657

[43] Peng Z Wu Y Wang J Gu S Wang Y Xue B . Development and validation of a glioma-associated mesenchymal stem cell-related gene prognostic index for predicting prognosis and guiding individualized therapy in glioma. Stem Cell Res Ther. (2023) 14:56. doi: 10.1186/s13287-023-03285-9 37005685 PMC10068170

[44] Wang QW Lin WW Zhu YJ . Comprehensive analysis of a TNF family based-signature in diffuse gliomas with regard to prognosis and immune significance. Cell Commun Signal. (2022) 20:6. doi: 10.1186/s12964-021-00814-y 35000592 PMC8744324

[45] He H Liang L Jiang S Liu Y Huang J Sun X . GINS2 regulates temozolomide chemosensitivity via the EGR1/ECT2 axis in gliomas. Cell Death Dis. (2024) 15:205. doi: 10.1038/s41419-024-06586-w 38467631 PMC10928080

[46] Zhang W Zhang L Dong H Peng H . TGIF2 is a potential biomarker for diagnosis and prognosis of glioma. Front Immunol. (2024) 15:1356833. doi: 10.3389/fimmu.2024.1356833 38629068 PMC11020094

[47] Zhao S Chi H Yang Q Chen S Wu C Lai G . Identification and validation of neurotrophic factor-related gene signatures in glioblastoma and Parkinson’s disease. Front Immunol. (2023) 14:1090040. doi: 10.3389/fimmu.2023.1090040 36825022 PMC9941742

[48] Wang Y Zhao W Liu X Guan G Zhuang M . ARL3 is downregulated and acts as a prognostic biomarker in glioma. J Transl Med. (2019) 17:210. doi: 10.1186/s12967-019-1914-3 31234870 PMC6591946

[49] Dai F Yuan Y Hao J Cheng X Zhou X Zhou L . PDCD2 as a prognostic biomarker in glioma correlates with Malignant phenotype. Genes Dis. (2024) 11:101106. doi: 10.1016/j.gendis.2023.101106 39022129 PMC11252777

[50] Xie Z Hua W Wang H . Comprehensive analysis of mitochondrial dynamic-related genes on their functions and prognostic values for glioblastoma multiforme. Genes Diseases. (2024) 11. doi: 10.1016/j.gendis.2023.101084 PMC11177063 38882002

[51] Wang Z Su G Dai Z Meng M Zhang H Fan F . Circadian clock genes promote glioma progression by affecting tumour immune infiltration and tumour cell proliferation. Cell Prolif. (2021) 54:e12988. doi: 10.1111/cpr.12988 33442944 PMC7941241

[52] Fortunato S Bergstrom CT Börner K Evans JA Helbing D Milojević S . Science of science. Science. (2018) 359. doi: 10.1126/science.aao0185 PMC5949209 29496846

[53] Carson KA Grossman SA Fisher JD Shaw EG . Prognostic factors for survival in adult patients with recurrent glioma enrolled onto the new approaches to brain tumor therapy CNS consortium phase I and II clinical trials. J Clin Oncol. (2007) 25:2601–6. doi: 10.1200/JCO.2006.08.1661 PMC4118746 17577040

[54] Gittleman H Sloan AE Barnholtz-Sloan JS . An independently validated survival nomogram for lower-grade glioma. Neuro Oncol. (2020) 22:665–74. doi: 10.1093/neuonc/noz191 PMC7229246 31621885

[55] Zhang Z Cai Q Wang J Wang J Yao Z Ji F Hang Y . Development and validation of a nomogram to predict cancer-specific survival in nonsurgically treated elderly patients with prostate cancer. Sci Rep. (2023) 13:17719. doi: 10.1038/s41598-023-44911-z 37853026 PMC10584808

[56] Cheplygina V de Bruijne M Pluim JPW . Not-so-supervised: A survey of semi-supervised, multi-instance, and transfer learning in medical image analysis. Med Image Anal. (2019) 54:280–96. doi: 10.1016/j.media.2019.03.009 30959445

[57] Patel AP J Fisher L Nichols E Abd-Allah F Abdela J Abdelalim A . Global, regional, and national burden of brain and other CNS cancer, 1990-2016: a systematic analysis for the Global Burden of Disease Study 2016. Lancet Neurol. (2019) 18:376–93. doi: 10.1016/S1474-4422(18)30468-X PMC6416167 30797715

[58] Ostrom QT Cote DJ Ascha M Kruchko C Barnholtz-Sloan JS . Adult glioma incidence and survival by race or ethnicity in the United States from 2000 to 2014. JAMA Oncol. (2018) 4:1254–62. doi: 10.1001/jamaoncol.2018.1789 PMC6143018 29931168

[59] Collins GS Dhiman P Ma J Schlussel MM Archer L Calster Van B . Evaluation of clinical prediction models (part 1): from development to external validation. Bmj. (2024) 384:e074819. doi: 10.1136/bmj-2023-074819 38191193 PMC10772854

[60] Retel Helmrich IR van Klaveren D Steyerberg EW . Research Note: Prognostic model research: overfitting, validation and application. J Physiother. (2019) 65:243–5. doi: 10.1016/j.jphys.2019.08.009 31521555

[61] Ma Z Lai Y Kleijn WB Song YZ Wang L Guo J . Variational bayesian learning for dirichlet process mixture of inverted dirichlet distributions in non-gaussian image feature modeling. IEEE Trans Neural Netw Learn Syst. (2019) 30:449–63. doi: 10.1109/TNNLS.2018.2844399 29994731

[62] Eklund M Norinder U Boyer S Carlsson L . Choosing feature selection and learning algorithms in QSAR. J Chem Inf Model. (2014) 54:837–43. doi: 10.1021/ci400573c 24460242

[63] Zhang J Sun R Lyu Y Liu C Liu Y Feng Y . Proteomic profiling of gliomas unveils immune and metabolism-driven subtypes with implications for anti-nucleotide metabolism therapy. Nat Commun. (2024) 15:10005. doi: 10.1038/s41467-024-54352-5 39562821 PMC11577044

[64] Buckner JC Shaw EG Pugh SL Chakravarti A Gilbert MR Barger GR . Radiation plus procarbazine, CCNU, and vincristine in low-grade glioma. N Engl J Med. (2016) 374:1344–55. doi: 10.1056/NEJMoa1500925 PMC5170873 27050206

[65] Eckel-Passow JE Lachance DH Molinaro AM Walsh KM Decker PA Sicotte H . Glioma groups based on 1p/19q, IDH, and TERT promoter mutations in tumors. N Engl J Med. (2015) 372:2499–508. doi: 10.1056/NEJMoa1407279 PMC4489704 26061753

[66] Shete S Hosking FJ Robertson LB Dobbins SE Sanson M Malmer B . Genome-wide association study identifies five susceptibility loci for glioma. Nat Genet. (2009) 41:899–904. doi: 10.1038/ng.407 19578367 PMC4501476

[67] Stupp R Brada M van den Bent MJ Tonn JC Pentheroudakis G . High-grade glioma: ESMO Clinical Practice Guidelines for diagnosis, treatment and follow-up. Ann Oncol. (2014) 25 Suppl 3:iii93–101. doi: 10.1093/annonc/mdu050 24782454

[68] Elguindy MM Young JS Ho WS Lu RO . Co-evolution of glioma and immune microenvironment. J Immunother Cancer. (2024) 12. doi: 10.1136/jitc-2024-009175 PMC11624716 39631850

[69] Sterne JA White IR Carlin JB Spratt M Royston P Kenward MG . Multiple imputation for missing data in epidemiological and clinical research: potential and pitfalls. Bmj. (2009) 338:b2393. doi: 10.1136/bmj.b2393 19564179 PMC2714692

[70] Yu Y Mai Y Zheng Y Shi L . Assessing and mitigating batch effects in large-scale omics studies. Genome Biol. (2024) 25:254. doi: 10.1186/s13059-024-03401-9 39363244 PMC11447944

[71] Wachinger C Rieckmann A Pölsterl S . Detect and correct bias in multi-site neuroimaging datasets. Med Image Anal. (2021) 67:101879. doi: 10.1016/j.media.2020.101879 33152602

[72] Tang B Zhu J Shi Y Wang Y Zhang X Chen B . Tumor cell-intrinsic MELK enhanced CCL2-dependent immunosuppression to exacerbate hepatocarcinogenesis and confer resistance of HCC to radiotherapy. Mol Cancer. (2024) 23:137. doi: 10.1186/s12943-024-02049-0 38970074 PMC11225310

[73] Stensrud MJ Aalen JM Aalen OO Valberg M . Limitations of hazard ratios in clinical trials. Eur Heart J. (2018) 40:1378–83. doi: 10.1093/eurheartj/ehy770 30500891

[74] Gregson J Sharples L Stone GW Burman CF Öhrn F Pocock S . Nonproportional hazards for time-to-event outcomes in clinical trials: JACC review topic of the week. J Am Coll Cardiol. (2019) 74:2102–12. doi: 10.1016/j.jacc.2019.08.1034 31623769

[75] Jiang N Wu Y Li C . Limitations of using COX proportional hazards model in cardiovascular research. Cardiovasc Diabetol. (2024) 23:219. doi: 10.1186/s12933-024-02302-2 38926821 PMC11210144

[76] Bardo M Huber C Benda N Brugger J Fellinger T Galaune V . Methods for non-proportional hazards in clinical trials: A systematic review. Stat Methods Med Res. (2024) 33:1069–92. doi: 10.1177/09622802241242325 PMC11162097 38592333

[77] Xu L Xu Y . Misuse of the Cox proportional hazards model and alternative approaches in kidney outcome research. Kidney Int. (2024) 106:1186. doi: 10.1016/j.kint.2024.08.026 39577991

[78] Carmona-Bayonas A Jiménez-Fonseca P Lamarca Á Barriuso J Castaño Á Benavent M . Prediction of progression-free survival in patients with advanced, well-differentiated, neuroendocrine tumors being treated with a somatostatin analog: the GETNE-TRASGU study. J Clin Oncol. (2019) 37:2571–80. doi: 10.1200/JCO.19.00980 PMC6768612 31390276

[79] Reckamp KL Behrendt CE Slavin TP Gray SW Castillo DK Koczywas M . Germline mutations and age at onset of lung adenocarcinoma. Cancer. (2021) 127:2801–6. doi: 10.1002/cncr.v127.15 PMC8794435 33858029

[80] Zhang H Liao J Zhang X Zhao E Liang X Luo S . Sex difference of mutation clonality in diffuse glioma evolution. Neuro-Oncology. (2018) 21:201–13. doi: 10.1093/neuonc/noy154 PMC6374767 30256978

[81] Melin BS Barnholtz-Sloan JS Wrensch MR Johansen C Il'yasova D Kinnersley B . Genome-wide association study of glioma subtypes identifies specific differences in genetic susceptibility to glioblastoma and non-glioblastoma tumors. Nat Genet. (2017) 49:789–94. doi: 10.1038/ng.3823 PMC5558246 28346443

[82] Ryba A Özdemir Z Nissimov N Hönikl L Neidert N Jakobs M . Insights from a multicenter study on adult H3 K27M-mutated glioma: Surgical resection’s limited influence on overall survival, ATRX as molecular prognosticator. Neuro-Oncology. (2024) 26:1479–93. doi: 10.1093/neuonc/noae061 PMC11300017 38507506

[83] Cai Z Apolinário S Baião AR Pacini C Sousa MD Vinga S . Synthetic augmentation of cancer cell line multi-omic datasets using unsupervised deep learning. Nat Commun. (2024) 15:10390. doi: 10.1038/s41467-024-54771-4 39614072 PMC11607321

[84] Zhang YP Zhang XY Cheng YT Li B Teng X-Z Zhang J . Artificial intelligence-driven radiomics study in cancer: the role of feature engineering and modeling. Mil Med Res. (2023) 10:22. doi: 10.1186/s40779-023-00458-8 37189155 PMC10186733

[85] Rajkomar A Dean J Kohane I . Machine learning in medicine. N Engl J Med. (2019) 380:1347–58. doi: 10.1056/NEJMra1814259 30943338

