# Supplementary material for: Limitations of nomogram models in predicting survival outcomes for glioma patients
Source: Front Immunol. 2025 Mar 18;16:1547506. doi: 10.3389/fimmu.2025.1547506 (PMC11959071; doi:10.3389/fimmu.2025.1547506)
Supplement: Supplementary file 1 [file DataSheet1.zip › Data Supplement 1.docx]

###### Data Supplement 1

**Supplementary Method 1.** Search strategies

We started data analysis in November 2024 after conducting a thorough literature review in the PubMed, Web of Science, and Embase databases that included publications published between January 1, 2004, and October 30, 2024.

Firstly, the titles of the identified records were scrutinized, with the abstracts being examined subsequently. Following this, potentially eligible studies were thoroughly evaluated to ensure their compliance with the inclusion criteria. The protocol stipulated that in case of any uncertainty regarding a study's eligibility, the project management group would review the inclusion until consensus was reached; however, this situation was not met. Bibliographic information for all articles included in our review (**Supplementary Table 45**).

**Search equations**

**PubMed**

((((((((((((((Glioma[MeSH Terms]) OR (Gliomas[Title/Abstract])) OR (Glial Cell Tumors[Title/Abstract])) OR (Glial Cell Tumor[Title/Abstract])) OR (Tumor, Glial Cell[Title/Abstract])) OR (Tumors, Glial Cell[Title/Abstract])) OR (Mixed Glioma[Title/Abstract])) OR (Glioma, Mixed[Title/Abstract])) OR (Gliomas, Mixed[Title/Abstract])) OR (Mixed Gliomas[Title/Abstract])) OR (Malignant Glioma[Title/Abstract])) OR (Glioma, Malignant[Title/Abstract])) OR (Gliomas, Malignant[Title/Abstract])) OR (Malignant Gliomas[Title/Abstract])) AND ((((((((((Nomograms[MeSH Terms]) OR (Nomogram[Title/Abstract])) OR (Partin Tables[Title/Abstract])) OR (Partin Table[Title/Abstract])) OR (Table, Partin[Title/Abstract])) OR (Tables, Partin[Title/Abstract])) OR (Partin Nomograms[Title/Abstract])) OR (Nomogram, Partin[Title/Abstract])) OR (Nomograms, Partin[Title/Abstract])) OR (Partin Nomogram[Title/Abstract]))

**Embase**

#27. #15 AND #26

#26. #16 OR #17 OR #18 OR #19 OR #20 OR #21 OR #22 OR #23 OR #24 OR #25

#25. 'nomogram, parti':ti,ab

#24. 'nomograms, parti':ti,ab

#23. 'partin nomogram':ti,ab

#22. 'partin nomograms':ti,ab

#21. 'table, partin':ti,ab

#20. 'tables, partin':ti,ab

#19. 'partin table':ti,ab

#18. 'partin tables':ti,ab

#17. 'nomogram':ti,ab

#16. 'nomograms'/exp

#15. #1 OR #2 OR #3 OR #4 OR #5 OR #6 OR #7 OR #8 OR #9 OR #10 OR #11 OR #12 OR #13 OR #14

#14. 'malignant gliomas':ti,ab

#13. 'gliomas, malignant':ti,ab

#12. 'glioma, malignant':ti,ab

#11. 'malignant glioma':ti,ab

#10. 'mixed gliomas':ti,ab

#9. 'gliomas, mixed':ti,ab

#8. 'glioma, mixed':ti,ab

#7. 'mixed glioma':ti,ab

#6. 'tumors, glial cell':ti,ab

#5. 'tumor, glial cell':ti,ab

#4. 'glial cell tumor':ti,ab

#3. 'glial cell tumors':ti,ab

#2. 'gliomas':ti,ab

#1. 'glioma'/exp

**Web of Science**

#3. #1 AND #2

#2. TS=(Nomogram* OR Nomogram OR Partin Table* OR Partin Table OR Table, Partin OR Table*, Partin OR Partin Nomogram* OR Nomogram, Partin OR Nomogram*, Partin OR Partin Nomogram)

#1. TS=(Glioma OR Glioma* OR Glial Cell Tumor* OR Glial Cell Tumor OR Tumor, Glial Cell OR Tumor*, Glial Cell OR Mixed Glioma OR Glioma, Mixed OR Glioma*, Mixed OR Mixed Glioma* OR Malignant Glioma OR Glioma, Malignant OR Glioma*, Malignant OR Malignant Glioma*)

**Supplementary Method 2.** Data extraction and preprocessing

Genomic and accompanying clinical data for samples in The Cancer Genome Atlas Research Network (TCGA, <http://cancergenome.nih.gov>) and CGGA (<http://www.cgga.org.cn>) were used for analysis. In addition, gene expression and glioma patient survival data in the Gravendeel database were downloaded from GlioVis (<http://gliovis.bioinfo.cnio.es/>). Metadata of patients in the TCGA-LGG, ZN-LGG, and SU-LGG cohorts collected by Liu et al. was obtained as well (<https://doi.org/10.1093/neuonc/noac154>). We processed the downloaded data according to the specific requirements for each dataset in the included literature (**Table 1**). Furthermore, if a risk score formula is constructed within the literature, we proceed to calculate the risk score for each corresponding patient according to the formula provided.

| **Supplementary Table 45.** Bibliographic details, inclusion or exclusion criteria for patients, and variables used in the nomogram of all articles encompassed in this review | | | | | | | |
| --- | --- | --- | --- | --- | --- | --- | --- |
| **Investigator** | **Title** | **DOI** | **Source** | **Year** | **Inclusion or exclusion criteria for patients** | **Variables used in the nomogram** | **Predicted Time** |
| An et al.^18^ | High expression of *SIGLEC7* may promote M2-type macrophage polarization leading to adverse prognosis in glioma patients | 10.3389/fimmu.2024.1411072 | Front Immunol | 2024 | Cases with incomplete information were handled by treating them as missing data. | *SIGLEC7* expression levels, WHO grade, IDH mutation status, 1p/19q status, patient age | 1-year, 2-year, 3-year, and 5-year OS |
| Chang et al.^19^ | *DDOST* Correlated with Malignancies and Immune Microenvironment in Gliomas | 10.3389/fimmu.2022.917014 | Front Immunol | 2022 | Patients with unknown or incomplete data were excluded. | DNA repair genes, age, PRS type, grade, IDH, 1p/19q, and MGMT status | 1-, 3-, and 5-year OS |
| Yu et al.^20^ | High Expression of *CKS2* Predicts Adverse Outcomes: A Potential Therapeutic Target for Glioma | 10.3389/fimmu.2022.881453 | Front Immunol | 2022 | NA | *CKS2* expression data, age, tumor grade, and IDH status | 1-, 3-, and 5-year OS |
| Jiang et al.^21^ | *CLCF1* Is a Novel Potential Immune-Related Target With Predictive Value for Prognosis and Immunotherapy Response in Glioma | 10.3389/fimmu.2022.810832 | Front Immunol | 2022 | Inclusion criteria: (a) primary glioma patients with OS > 1 month; (b) patients with mRNA sequencing data; and (c) complete WHO Grade classification data for each patient. | WHO grade, *CLCF1* expression | 1-, 3-, and 5-year OS |
| Wang et al.^22^ | *CLEC7A* regulates M2 macrophages to suppress the immune microenvironment and implies poorer prognosis of glioma | 10.3389/fimmu.2024.1361351 | Front Immunol | 2024 | Inclusion criteria: (a) computation of the mean for any duplicated patient sequencing outcomes within the database; and (b) each selected sample had to include comprehensive clinical information about the patient, including survival time, survival status, age, and gender, among others. | TCGA: *CLEC7A* expression, grade, age, IDH status  CGGA: *CLEC7A* expression, PRS type, age, IDH, 1p/19q status | 1-, 3-, and 5-year OS |
| Song et al.^23^ | Novel prognostic biomarker *TBC1D1* is associated with immunotherapy resistance in gliomas | 10.3389/fimmu.2024.1372113 | Front Immunol | 2024 | NA | *TBC1D1* expression, age, race, gender, grade, IDH1 status, histological type and laterality | 1-, 3-, and 5-year OS |
| Chen et al.^25^ | Antigen Presentation Machinery Signature-Derived CALR Mediates Migration, Polarization of Macrophages in Glioma and Predicts Immunotherapy Response | 10.3389/fimmu.2022.833792 | Front Immunol | 2022 | Samples with incomplete overall survival information of patients were excluded. Samples with missed information of IDH status were also excluded. | RS, tumor grade, age, IDH, 1p/19q status | 1-, 3-, 4-, and 5-year OS |
| Han et al.^26^ | Comprehensive characterization of *TNFSF14/LIGHT* with implications in prognosis and immunotherapy of human gliomas | 10.3389/fimmu.2022.1025286 | Front Immunol | 2022 | NA | *TNFSF14/LIGHT* expression level, WHO grade, gender, age, radiotherapy, chemotherapy, IDH, 1p/19q, and MGMT status | 60-, 90-, and 120-month OS |
| Zhang et al.^27^ | Construction and validation of a cuproptosis-related prognostic model for glioblastoma | 10.3389/fimmu.2023.1082974 | Front Immunol | 2023 | Samples without OS information were excluded. | RS, age, gender, and IDH1 mutation status | 0.5-, 1-, and 1.5-year OS |
| Wang et al.^28^ | Integrated machine learning methods identify *FNDC3B* as a potential prognostic biomarker and correlated with immune infiltrates in glioma | 10.3389/fimmu.2022.1027154 | Front Immunol | 2022 | NA | Age, grade, IDH status, and *FNDC3B* expression | 2-, 3-, and 5-year OS |
| Zhang et al.^29^ | Molecular and immunological features of *TREM1* and its emergence as a prognostic indicator in glioma | 10.3389/fimmu.2024.1324010 | Front Immunol | 2024 | NA | *TREM1* expression, grade, gender, age, radiotherapy, chemotherapy, IDH status | 1-, 3-, and 5-year OS |
| Ma et al.^30^ | *SAMD9* Is Relating With M2 Macrophage and Remarkable Malignancy Characters in Low-Grade Glioma | 10.3389/fimmu.2021.659659 | Front Immunol | 2021 | NA | TCGA: age, grade, IDH status, *SAMD9* expression  CGGA325: grade, 1p/19q, radiotherapy status, *SAMD9* expression | 1-, 3-, and 5-year OS |
| Ge et al.^31^ | *TP53I13* promotes metastasis in glioma via macrophages, neutrophils, and fibroblasts and is a potential prognostic biomarker | 10.3389/fimmu.2022.974346 | Front Immunol | 2022 | NA | *TP53I13* expression level, WHO grade, 1p/19q codeletion status, IDH mutation status, gender, and age | 1-, 3-, and 5-year OS |
| Peng et al.^32^ | Phosducin-like 3 is a novel prognostic and onco-immunological biomarker in glioma: A multi-omics analysis with experimental verification | 10.3389/fimmu.2023.1128151 | Front Immunol | 2023 | NA | Age, WHO grade, IDH status, 1p/19q codeletion and *PDCL3* expression | 1-, 3-, and 5-year OS |
| Zhi et al.^33^ | PLEKHA4 is a novel prognostic biomarker that reshapes the tumor microenvironment in lower-grade glioma | 10.3389/fimmu.2023.1128244 | Front Immunol | 2023 | NA | WHO grade, age, IDH status, primary therapy outcome, and *PLEKHA4* expression | 1-, 3-, and 5-year OS  1-, 3-, and 5-year DSS |
| Zhang et al.^34^ | An Immune-Related Signature for Predicting the Prognosis of Lower-Grade Gliomas | 10.3389/fimmu.2020.603341 | Front Immunol | 2020 | Samples with missing information or with a survival time less than 90 days were excluded. | Age, grade, IDH1 status, and immune-related risk | 1-, 3-, and 5-year OS |
| Li et al.^35^ | Establishment and validation of a novel prognostic model for lower-grade glioma based on senescence-related genes | 10.3389/fimmu.2022.1018942 | Front Immunol | 2022 | NA | Age, gender, WHO grade, IDH, 1p/19q status, SnG-Risk score | 1-, 3-, and 5-year OS |
| Li et al.^36^ | Identification of heterogeneous subtypes and a prognostic model for gliomas based on mitochondrial dysfunction and oxidative stress-related genes | 10.3389/fimmu.2023.1183475 | Front Immunol | 2023 | NA | Age, gender, WHO grade, IDH, 1p/19q status, RS | 1-, 3-, and 5-year OS |
| Wang et al.^37^ | The CXCL Family Contributes to Immunosuppressive Microenvironment in Gliomas and Assists in Gliomas Chemotherapy | 10.3389/fimmu.2021.731751 | Front Immunol | 2021 | NA | RS, cancer type, age, and 1p/19q status | 3-, 5-year OS |
| Zhu et al.^38^ | Cuprotosis clusters predict prognosis and immunotherapy response in low-grade glioma | 10.1007/s10495-023-01880-y | Apoptosis | 2023 | NA | Gender, grade, age, and RS | 1-, 3-, and 5-year OS |
| Geng et al.^39^ | *SIRPB1* regulates inflammatory factor expression in the glioma microenvironment via SYK: functional and bioinformatics insights | 10.1186/s12967-024-05149-z | J Transl Med | 2024 | NA | IDH status, primary therapy outcome, age, and *SIRPB1* expression | 1-, 3-, 5-year OS and PFI |
| Yu et al.^40^ | Crosstalk of different cell-death patterns predicts prognosis and drug sensitivity in glioma | 10.1016/j.compbiomed.2024.108532 | Comput Biol Med | 2024 | NA | CDRS, age, WHO grade, IDH, 1p/19q, and MGMT status | 2-, 3-, and 5-year OS |
| Zhou et al.^41^ | Identification of telomere-associated gene signatures to predict prognosis and drug sensitivity in glioma | 10.1016/j.compbiomed.2023.107750 | Comput Biol Med | 2023 | NA | Age, RS | 1-, 3-, and 5-year OS |
| Wu et al.^42^ | Identification of *CREB5* as a prognostic and immunotherapeutic biomarker in glioma through multi-omics pan-cancer analysis | 10.1016/j.compbiomed.2024.108307 | Comput Biol Med | 2024 | NA | Age, gender, WHO grade, IDH, 1p/19q status, and RS | 1-, 3-, and 5-year OS |
| Peng et al.^43^ | Development and validation of a glioma-associated mesenchymal stem cell-related gene prognostic index for predicting prognosis and guiding individualized therapy in glioma | 10.1186/s13287-023-03285-9 | Stem Cell Res Ther | 2023 | Cases without survival data or overall survival < 30 days or without definitive histopathological diagnosis were excluded. | Age, grade, and GA-MSCRGPI | 2-, 3-, and 5-year OS |
| Wang et al.^44^ | Comprehensive analysis of a TNF family based-signature in diffuse gliomas with regard to prognosis and immune significance | 10.1186/s12964-021-00814-y | Cell Commun Signal | 2022 | NA | Age, RS | 1-, 3-, and 5-year OS |
| Lin et al.^11^ | *VMP1*, a novel prognostic biomarker, contributes to glioma development by regulating autophagy | 10.1186/s12974-021-02213-z | J Neuroinflammation | 2021 | NA | *VMP1* expression, age, primary recurrence type, grade, radiotherapy status, chemotherapy status, IDH mutation status, and 1p/19q codeletion status | 1-, 3-, and 5-year OS |
| Han et al.^10^ | Immune checkpoint molecule herpes virus entry mediator is overexpressed and associated with poor prognosis in human glioblastoma | 10.1016/j.ebiom.2019.04.002 | EBioMedicine | 2019 | All glioma tissues were histopathologically confirmed to be grade II, III or IV gliomas. The clinical end point event, OS, was calculated from the date of initial diagnosis until death or last follow-up examination. | WHO grade, gender, age, IDH, radiotherapy, chemotherapy status, HVEM expression level | 1-, 3-, and 5-year OS |
| Liu et al.^45^ | Clinical significance and molecular annotation of cellular morphometric subtypes in lower-grade gliomas discovered by machine learning | 10.1093/neuonc/noac154 | Neuro Oncol | 2023 | The inclusion criteria were primary LGG and GBM with diagnostic slides and OS information available. | Age, grade, IDH, ATRX mutation status, and subtype | 3-, 5-year OS |
| He et al.^46^ | GINS2 regulates temozolomide chemosensitivity via the EGR1/ECT2 axis in gliomas | 10.1038/s41419-024-06586-w | Cell Death Dis | 2024 | NA | RS, age, gender and glioma grade typing | 1-, 3-, and 5-year OS |
| Zeng et al.^24^ | A specific immune signature for predicting the prognosis of glioma patients with IDH1-mutation and guiding immune checkpoint blockade therapy | 10.3389/fimmu.2022.1001381 | Front Immunol | 2022 | Glioma patients without IDH1-mutation information and survival information were excluded. | Age, gender, grade, RS | 1-, 3-, and 5-year OS |
| Zhang et al.^47^ | *TGIF2* is a potential biomarker for diagnosis and prognosis of glioma | 10.3389/fimmu.2024.1356833 | Front Immunol | 2024 | NA | *TGIF2* expression level, age, histological type, primary therapy outcome, 1p/19q status | 1-, 3-, and 5-year OS |
| Zhao et al.^48^ | Identification and validation of neurotrophic factor-related gene signatures in glioblastoma and Parkinson's disease | 10.3389/fimmu.2023.1090040 | Front Immunol | 2023 | NA | Age, gender, IDH mutation status, and RS | 1-, 1.5-, and 2-year OS |
| Wang et al.^49^ | *ARL3* is downregulated and acts as a prognostic biomarker in glioma | 10.1186/s12967-019-1914-3. | J Transl Med | 2019 | The inclusion criteria for data extraction in the predictive model were patients diagnosed with WHO grade II–IV glioma. The exclusion criteria included patients with missing or incomplete data such as survival status and time, age, sex, grade, and IDH status. | *ARL3* expression level, age, sex, WHO grade and IDH status | 3-, 5-year OS |
| Song et al.^17^ | Prognostic and predictive value of an immune infiltration signature in diffuse lower-grade gliomas | 10.1172/jci.insight.133811 | JCI Insight | 2020 | The exclusion criteria for the downloaded data were as follows: (a) brainstem glioma, (b) recurrent glioma, (c) patients who underwent biopsy alone without tumor resection, and (d) patients with survival data ≤ 30 days. | RS, age, and grade | 3-, 5-year OS |
| Dai et al.^50^ | *PDCD2* as a prognostic biomarker in glioma correlates with malignant phenotype | 10.1016/j.gendis.2023.101106 | Genes Dis | 2023 | NA | *PDCD2* expression, WHO grade | 1-, 3-, and 5-year OS  1-, 3-, and 5-year DSS  1-, 3-, and 5-year PFI |
| Xie et al.^51^ | Comprehensive analysis of mitochondrial dynamic-related genes on their functions and prognostic values for glioblastoma multiforme | 10.1016/j.gendis.2023.101084 | Genes Dis | 2023 | NA | Race, age, gender, *MFF*, *MSTO*1, *MFN1*, *MIEF1*, *MIEF2* | 1-, 2-year OS |
| Zeng et al.^14^ | Comprehensive profiling identifies a novel signature with robust predictive value and reveals the potential drug resistance mechanism in glioma | 10.1186/s12964-019-0492-6 | Cell Commun Signal | 2020 | NA | RS, grade, and radiotherapy status | 1-, 3-, and 5-year OS |
| Wang et al.^52^ | Circadian clock genes promote glioma progression by affecting tumour immune infiltration and tumour cell proliferation | 10.1111/cpr.12988 | Cell Prolif | 2021 | NA | RS, age, 1p/19q status | 3-, 5-year OS |
| **Abbreviations**:1p/19q, chromosome arms 1p and 19q; ARL3, ADP-ribosylation factor-like 3; ATRX, alpha thalassemia/mental retardation syndrome X-linked; CKS2, cyclin-dependent kinase regulatory subunit 2; CLCF1, cardiotrophin-like cytokine factor 1; CLEC7A, C-type lectin domain family 7, member A; CREB5, cyclic adenosine monophosphate (cAMP)-response element-binding protein 5; DSS, disease-specific survival; FNDC3B, fibronectin type III domain containing 3B; GA-MSCRGPI, glioma-associated mesenchymal stem cells related gene prognostic index; HVEM, herpes virus entry mediator; IDH, isocitrate dehydrogenase; MFF, mitochondrial fission factor; MFN1, mitofusin-1; MIEF1, mitochondrial elongation factor 1; MIEF2, mitochondrial elongation factor 2;MSTO1, Misato homolog 1; NA, not available; O6-methylguanine-DNA methyltransferase, MGMT; OS, overall survival; PDCD2, programmed cell death 2; PDCL3, phosducin-like 3; PFI, progression-free interval; PLEKHA4, pleckstrin homology domain containing A4; PRS, primary, recurrent, secondary; RS, risk score; SAMD9, sterile alpha motif domain-containing protein 9; SIGLEC7, sialic acid-binding immunoglobulin-like lectin 7; SIRPB1, signal regulatory protein beta 1; SnG, senescence genes; TBC1D1, TBC1 domain family member 1; TGIF2, TGFB-induced factor homeobox 2; TNFSF14, tumor necrosis factor superfamily member 14; TP53I13, tumor protein p53 inducible protein 13; TREM1, triggering receptor expressed on myeloid cells 1; VMP1, vacuole membrane protein 1; WHO, world health organization | | | | | | | |
