# Supplementary material for: Limitations of nomogram models in predicting survival outcomes for glioma patients
Source: Front Immunol. 2025 Mar 18;16:1547506. doi: 10.3389/fimmu.2025.1547506 (PMC11959071; doi:10.3389/fimmu.2025.1547506)
Supplement: Supplementary file 3 [file DataSheet3.zip › Data Supplement 3.docx]

###### Data Supplement 3

**Supplementary Table 1.** PH assumption test on the nomogram model proposed by An et al.^18^

**Supplementary Table 2.** PH assumption test on the nomogram model proposed by Chang et al.^19^

**Supplementary Table 3.** PH assumption test on the nomogram model proposed by Yu et al.^20^

**Supplementary Table 4.** PH assumption test on the nomogram model proposed by Jiang et al.^21^

**Supplementary Table 5.** PH assumption test on the nomogram model proposed by Wang et al.^22^

**Supplementary Table 6.** PH assumption test on the nomogram model proposed by Song et al.^23^

**Supplementary Table 7.** PH assumption test on the nomogram model proposed by Chen et al.^25^

**Supplementary Table 8.** PH assumption test on the nomogram model proposed by Han et al.^26^

**Supplementary Table 9.** PH assumption test on the nomogram model proposed by Zhang et al.^27^

**Supplementary Table 10.** PH assumption test on the nomogram model proposed by Wang et al.^28^

**Supplementary Table 11.** PH assumption test on the nomogram model proposed by Zhang et al.^29^

**Supplementary Table 12.** PH assumption test on the nomogram model proposed by Ma et al.^30^

**Supplementary Table 13.** PH assumption test on the nomogram model proposed by Ge et al.^31^

**Supplementary Table 14.** PH assumption test on the nomogram model proposed by Peng et al.^32^

**Supplementary Table 15.** PH assumption test on the nomogram model proposed by Zhi et al.^33^

**Supplementary Table 16.** PH assumption test on the nomogram model proposed by Zhang et al.^34^

**Supplementary Table 17.** PH assumption test on the nomogram model proposed by Li et al.^35^

**Supplementary Table 18.** PH assumption test on the nomogram model proposed by Li et al.^36^

**Supplementary Table 19.** PH assumption test on the nomogram model proposed by Wang et al.^37^

**Supplementary Table 20.** PH assumption test on the nomogram model proposed by Zhu et al.^38^

**Supplementary Table 21.** PH assumption test on the nomogram model proposed by Geng et al.^39^

**Supplementary Table 22.** PH assumption test on the nomogram model proposed by Yu et al.^40^

**Supplementary Table 23.** PH assumption test on the nomogram model proposed by Zhou et al.^41^

**Supplementary Table 24.** PH assumption test on the nomogram model proposed by Wu et al.^42^

**Supplementary Table 25.** PH assumption test on the nomogram model proposed by Peng et al.^43^

**Supplementary Table 26.** PH assumption test on the nomogram model proposed by Wang et al.^44^

**Supplementary Table 27.** PH assumption test on the nomogram model proposed by Lin et al.^11^

**Supplementary Table 28.** PH assumption test on the nomogram model proposed by Han et al.^10^

**Supplementary Table 29.** PH assumption test on the nomogram model proposed by Liu et al.^45^

**Supplementary Table 30.** PH assumption test on the nomogram model proposed by He et al.^46^

**Supplementary Table 31.** PH assumption test on the nomogram model proposed by Zeng et al.^24^

**Supplementary Table 32.** PH assumption test on the nomogram model proposed by Zhang et al.^47^

**Supplementary Table 33.** PH assumption test on the nomogram model proposed by Zhao et al.^48^

**Supplementary Table 34.** PH assumption test on the nomogram model proposed by Wang et al.^49^

**Supplementary Table 35.** PH assumption test on the nomogram model proposed by Song et al.^17^

**Supplementary Table 36.** PH assumption test on the nomogram model proposed by Dai et al.^50^

**Supplementary Table 37.** PH assumption test on the nomogram model proposed by Xie et al.^51^

**Supplementary Table 38.** PH assumption test on the nomogram model proposed by Zeng et al.^14^

**Supplementary Table 39.** PH assumption test on the nomogram model proposed by Wang et al.^52^

**Supplementary Table 40.** Metadata of patients in TCGA-LGG cohort acquired from Liu et al.^45^

**Supplementary Table 41.** Metadata of patients in ZN-LGG cohort acquired from Liu et al.^45^

**Supplementary Table 42**. Metadata of patients in SU-LGG cohort acquired from Liu et al.^45^

**Supplementary Table 43.** PH assumption test on the first proposed nomogram model

**Supplementary Table 44.** PH assumption test on the reconstructed nomogram model

| **Supplementary Table 1.** PH assumption test on the nomogram model proposed by An et al.^18^ | | | | |
| --- | --- | --- | --- | --- |
|  | **Characteristic** | **Chi-Square value** | **Degree of freedom** | ***P*-value** |
| **TCGA database** | SIGLEC7 | 3.1248 | 1 | 0.0771 |
|  | WHO grade | 9.5 | 2 | 0.0087 |
|  | IDH status | 26.936 | 1 | 2.1e-07 |
|  | 1p/19q codeletion | 1.7444 | 1 | 0.1866 |
|  | Age | 6.5086 | 1 | 0.0107 |
|  | Global | 30.114 | 6 | **3.74e-05** |
| **CGGA database** | SIGLEC7 | 3.7866 | 1 | 0.0517 |
|  | WHO grade | 13.988 | 2 | 0.0009 |
|  | IDH status | 16.971 | 1 | 3.8e-05 |
|  | 1p/19q codeletion | 12.593 | 1 | 0.0004 |
|  | Age | 0.070846 | 1 | 0.7901 |
|  | Global | 26.543 | 6 | **0.0002** |
| If the *p*-value in the global test > 0.05, it indicates that the nomogram adheres to the PH assumption. | | | | |

| **Supplementary Table 2.** PH assumption test on the nomogram model proposed by Chang et al.^19^ | | | | |
| --- | --- | --- | --- | --- |
|  | **Characteristic** | **Chi-Square value** | **Degree of freedom** | ***P*-value** |
| **CGGA325 database** | IDH status | 0.013939 | 1 | 0.9060 |
|  | Gender | 4.5436 | 1 | 0.0330 |
|  | MGMT | 1.4272 | 1 | 0.2322 |
|  | Age | 3.5966 | 1 | 0.0579 |
|  | 1p/19q codeletion | 0.099991 | 1 | 0.7518 |
|  | WHO grade | 10.551 | 2 | 0.0051 |
|  | DDOST | 2.8791 | 1 | 0.0897 |
|  | PRS type | 10.079 | 2 | 0.0065 |
|  | Global | 22.115 | 10 | **0.0145** |
| If the *p*-value in the global test > 0.05, it indicates that the nomogram adheres to the PH assumption. | | | | |

| **Supplementary Table 3.** PH assumption test on the nomogram model proposed by Yu et al.^20^ | | | | |
| --- | --- | --- | --- | --- |
|  | **Characteristic** | **Chi-Square value** | **Degree of freedom** | ***P*-value** |
| **TCGA database** | Age | 4.5525 | 1 | 0.0329 |
|  | WHO grade | 10.834 | 1 | 0.0010 |
|  | IDH status | 38.737 | 1 | 4.85e-10 |
|  | CKS2 | 20.804 | 1 | 5.09e-06 |
|  | Global | 42.569 | 4 | **1.27e-08** |
| If the *p*-value in the global test > 0.05, it indicates that the nomogram adheres to the PH assumption. | | | | |

| **Supplementary Table 4.** PH assumption test on the nomogram model proposed by Jiang et al.^21^ | | | | |
| --- | --- | --- | --- | --- |
|  | **Characteristic** | **Chi-Square value** | **Degree of freedom** | ***P*-value** |
| **TCGA database** | WHO grade | 6.2256 | 2 | 0.0445 |
|  | CLCF1 | 9.5578 | 1 | 0.0020 |
|  | Global | 11.109 | 3 | **0.0112** |
| **CGGA database** | WHO grade | 21.457 | 2 | 2.19e-05 |
|  | CLCF1 | 0.98316 | 1 | 0.3214 |
|  | Global | 21.458 | 3 | **8.45e-05** |
| If the *p*-value in the global test > 0.05, it indicates that the nomogram adheres to the PH assumption. | | | | |

| **Supplementary Table 5.** PH assumption test on the nomogram model proposed by Wang et al.^22^ | | | | |
| --- | --- | --- | --- | --- |
|  | **Characteristic** | **Chi-Square value** | **Degree of freedom** | ***P*-value** |
| **TCGA database** | CLEC7A | 4.2419 | 1 | 0.0394 |
|  | WHO grade | 8.2187 | 2 | 0.0164 |
|  | Age | 6.9371 | 1 | 0.0084 |
|  | IDH status | 20.856 | 1 | 4.95e-06 |
|  | Global | 23.927 | 5 | **0.0002** |
| **CGGA database** | CLEC7A | 3.2496 | 1 | 0.0714 |
|  | PRS type | 6.0886 | 1 | 0.0136 |
|  | Age | 0.020384 | 1 | 0.8865 |
|  | IDH status | 21.576 | 1 | 3.4e-06 |
|  | 1p/19q codeletion | 15.641 | 1 | 7.66e-05 |
|  | Global | 34.887 | 5 | **1.58e-06** |
| If the *p*-value in the global test > 0.05, it indicates that the nomogram adheres to the PH assumption. | | | | |

| **Supplementary Table 6.** PH assumption test on the nomogram model proposed by Song et al.^23^ | | | | |
| --- | --- | --- | --- | --- |
|  | **Characteristic** | **Chi-Square value** | **Degree of freedom** | ***P*-value** |
| **TCGA database** | Age | 1.0163 | 1 | 0.3134 |
|  | Race | 0.53373 | 1 | 0.4650 |
|  | Gender | 0.89051 | 1 | 0.3453 |
|  | IDH status | 18.294 | 1 | 1.89e-05 |
|  | WHO grade | 4.1073 | 1 | 0.0427 |
|  | Histological type | 5.1727 | 2 | 0.0753 |
|  | Laterality | 0.50718 | 2 | 0.7760 |
|  | TBC1D1 | 4.68 | 1 | 0.0305 |
|  | Global | 26.278 | 10 | **0.0034** |
| If the *p*-value in the global test > 0.05, it indicates that the nomogram adheres to the PH assumption. | | | | |

| **Supplementary Table 7.** PH assumption test on the nomogram model proposed by Chen et al.^25^ | | | | |
| --- | --- | --- | --- | --- |
|  | **Characteristic** | **Chi-Square value** | **Degree of freedom** | ***P*-value** |
| **TCGA database** | Risk score | 0.38704 | 1 | 0.5339 |
|  | WHO grade | 2.9751 | 2 | 0.2259 |
|  | Age | 2.3368 | 1 | 0.1263 |
|  | IDH status | 13.644 | 1 | 0.0002 |
|  | 1p/19q codeletion | 0.076765 | 1 | 0.7817 |
|  | Global | 18.83 | 6 | **0.0045** |
| If the *p*-value in the global test > 0.05, it indicates that the nomogram adheres to the PH assumption. | | | | |

| **Supplementary Table 8.** PH assumption test on the nomogram model proposed by Han et al.^26^ | | | | |
| --- | --- | --- | --- | --- |
|  | **Characteristic** | **Chi-Square value** | **Degree of freedom** | ***P*-value** |
| **CGGA693 database** | WHO grade | 10.34 | 2 | 0.0057 |
|  | Gender | 4.2598 | 1 | 0.0390 |
|  | Age | 0.19946 | 1 | 0.6552 |
|  | Radiotherapy status | 4.8354 | 1 | 0.0279 |
|  | Chemotherapy status | 0.16756 | 1 | 0.6823 |
|  | IDH status | 14.381 | 1 | 0.0001 |
|  | 1p/19q codeletion | 7.9006 | 1 | 0.0049 |
|  | MGMT | 2.6555 | 1 | 0.1032 |
|  | LIGHT | 3.1066 | 1 | 0.0780 |
|  | Global | 36.528 | 10 | **6.83e-05** |
| If the *p*-value in the global test > 0.05, it indicates that the nomogram adheres to the PH assumption. | | | | |

| **Supplementary Table 9.** PH assumption test on the nomogram model proposed by Zhang et al.^27^ | | | | |
| --- | --- | --- | --- | --- |
|  | **Characteristic** | **Chi-Square value** | **Degree of freedom** | ***P*-value** |
| **TCGA database** | Gender | 1.8536 | 1 | 0.1734 |
|  | IDH status | 11.821 | 1 | 0.0006 |
|  | Age | 4.2528 | 1 | 0.0392 |
|  | Risk score | 30.935 | 1 | 2.67e-08 |
|  | Global | 32.934 | 4 | **1.23e-06** |
| If the *p*-value in the global test > 0.05, it indicates that the nomogram adheres to the PH assumption. | | | | |

| **Supplementary Table 10.** PH assumption test on the nomogram model proposed by Wang et al.^28^ | | | | |
| --- | --- | --- | --- | --- |
|  | **Characteristic** | **Chi-Square value** | **Degree of freedom** | ***P*-value** |
| **TCGA database** | FNDC3B | 2.5251 | 1 | 0.1120 |
|  | Age | 2.9327 | 1 | 0.0868 |
|  | WHO grade | 2.3163 | 1 | 0.1280 |
|  | IDH status | 15.443 | 1 | 8.5e-05 |
|  | Global | 22.503 | 4 | **0.0002** |
| **CGGA301 database** | FNDC3B | 3.3014 | 1 | 0.0692 |
|  | Age | 0.31869 | 1 | 0.5724 |
|  | WHO grade | 6.2766 | 1 | 0.0122 |
|  | IDH status | 0.032732 | 1 | 0.8564 |
|  | Global | 9.4125 | 4 | 0.0516 |
| **CGGA325 database** | FNDC3B | 0.2348 | 1 | 0.6280 |
|  | Age | 0.44701 | 1 | 0.5038 |
|  | WHO grade | 12.249 | 1 | 0.0005 |
|  | IDH status | 0.042336 | 1 | 0.8370 |
|  | Global | 15.681 | 4 | **0.0035** |
| **CGGA693 database** | FNDC3B | 0.63967 | 1 | 0.4238 |
|  | Age | 0.88887 | 1 | 0.3458 |
|  | WHO grade | 6.5418e-05 | 1 | 0.9935 |
|  | IDH status | 8.6465 | 1 | 0.0033 |
|  | Global | 9.418 | 4 | 0.0515 |
| If the *p*-value in the global test > 0.05, it indicates that the nomogram adheres to the PH assumption. | | | | |

| **Supplementary Table 11.** PH assumption test on the nomogram model proposed by Zhang et al.^29^ | | | | |
| --- | --- | --- | --- | --- |
|  | **Characteristic** | **Chi-Square value** | **Degree of freedom** | ***P*-value** |
| **TCGA database** | TREM1 | 1.1844 | 1 | 0.2765 |
|  | WHO grade | 11.342 | 2 | 0.0034 |
|  | Gender | 2.8773 | 1 | 0.0898 |
|  | Age | 1.3837 | 1 | 0.2395 |
|  | Radiotherapy status | 9.4439 | 1 | 0.0021 |
|  | Chemotherapy status | 0.10415 | 1 | 0.7469 |
|  | IDH status | 17.229 | 1 | 3.31e-05 |
|  | Global | 34.465 | 8 | **3.35e-05** |
| If the *p*-value in the global test > 0.05, it indicates that the nomogram adheres to the PH assumption. | | | | |

| **Supplementary Table 12.** PH assumption test on the nomogram model proposed by Ma et al.^30^ | | | | |
| --- | --- | --- | --- | --- |
|  | **Characteristic** | **Chi-Square value** | **Degree of freedom** | ***P*-value** |
| **TCGA database** | Age | 3.2941 | 1 | 0.0695 |
|  | WHO grade | 1.8516 | 1 | 0.1736 |
|  | IDH status | 12.118 | 1 | 0.0005 |
|  | SAMD9 | 1.0043 | 1 | 0.3163 |
|  | Global | 19.011 | 4 | **0.0008** |
| **CGGA database** | WHO grade | 10.255 | 1 | 0.0014 |
|  | 1p/19q codeletion | 1.282 | 1 | 0.2575 |
|  | Radio status | 5.4447 | 1 | 0.0196 |
|  | SAMD9 | 0.029981 | 1 | 0.8625 |
|  | Global | 15.715 | 4 | **0.0034** |
| If the *p*-value in the global test > 0.05, it indicates that the nomogram adheres to the PH assumption. | | | | |

| **Supplementary Table 13.** PH assumption test on the nomogram model proposed by Ge et al.^31^ | | | | |
| --- | --- | --- | --- | --- |
|  | **Characteristic** | **Chi-Square value** | **Degree of freedom** | ***P*-value** |
| **TCGA database** | WHO grade | 11.581 | 1 | 0.0007 |
|  | 1p/19q codeletion | 3.3034 | 1 | 0.0691 |
|  | IDH status | 42.186 | 1 | 8.3e-11 |
|  | Gender | 6.1618 | 1 | 0.0131 |
|  | Age | 6.6147 | 1 | 0.0101 |
|  | TP53I13 | 5.747 | 1 | 0.0165 |
|  | Global | 47.074 | 6 | **1.81e-08** |
| If the *p*-value in the global test > 0.05, it indicates that the nomogram adheres to the PH assumption. | | | | |

| **Supplementary Table 14.** PH assumption test on the nomogram model proposed by Peng et al.^32^ | | | | |
| --- | --- | --- | --- | --- |
|  | **Characteristic** | **Chi-Square value** | **Degree of freedom** | ***P*-value** |
| **TCGA database** | Age | 6.426 | 1 | 0.0112 |
|  | WHO grade | 7.4556 | 2 | 0.0240 |
|  | IDH status | 25.304 | 1 | 4.9e-07 |
|  | 1p/19q codeletion | 1.465 | 1 | 0.2261 |
|  | PDCL3 | 5.5687 | 1 | 0.0183 |
|  | Global | 30.203 | 6 | **3.6e-05** |
| If the *p*-value in the global test > 0.05, it indicates that the nomogram adheres to the PH assumption. | | | | |

| **Supplementary Table 15.** PH assumption test on the nomogram model proposed by Zhi et al.^33^ | | | | |
| --- | --- | --- | --- | --- |
|  | **Characteristic** | **Chi-Square value** | **Degree of freedom** | ***P*-value** |
| **TCGA database (OS)** | WHO grade | 3.378 | 1 | 0.0661 |
|  | Age | 0.35552 | 1 | 0.5510 |
|  | IDH status | 24.101 | 1 | 9.14e-07 |
|  | Primary therapy outcome | 23.378 | 3 | 3.37e-05 |
|  | PLEKHA4 | 10.474 | 1 | 0.0012 |
|  | Global | 37.616 | 7 | **3.58e-06** |
| **TCGA database (DSS)** | WHO grade | 2.7495 | 1 | 0.0973 |
|  | Age | 0.086291 | 1 | 0.7689 |
|  | IDH status | 22.662 | 1 | 1.93e-06 |
|  | Primary therapy outcome | 24.662 | 3 | 1.82e-05 |
|  | PLEKHA4 | 9.5047 | 1 | 0.0020 |
|  | Global | 38.877 | 7 | **2.06e-06** |
| If the *p*-value in the global test > 0.05, it indicates that the nomogram adheres to the PH assumption. OS, overall survival; DSS, disease-specific survival. | | | | |

| **Supplementary Table 16.** PH assumption test on the nomogram model proposed by Zhang et al.^34^ | | | | |
| --- | --- | --- | --- | --- |
|  | **Characteristic** | **Chi-Square value** | **Degree of freedom** | ***P*-value** |
| **TCGA database** | WHO grade | 3.0297 | 1 | 0.0818 |
|  | IDH status | 13.18 | 1 | 0.0003 |
|  | CRG score | 0.085189 | 1 | 0.7704 |
|  | Age | 4.1187 | 1 | 0.0424 |
|  | Global | 21.386 | 4 | **0.0003** |
| **CGGA database** | WHO grade | 17.48 | 1 | 2.9e-05 |
|  | IDH status | 0.0034287 | 1 | 0.9533 |
|  | CRG score | 4.2969 | 1 | 0.0382 |
|  | Age | 0.57522 | 1 | 0.4482 |
|  | Global | 23.545 | 4 | **9.85e-05** |
| If the *p*-value in the global test > 0.05, it indicates that the nomogram adheres to the PH assumption. | | | | |

| **Supplementary Table 17.** PH assumption test on the nomogram model proposed by Li et al.^35^ | | | | |
| --- | --- | --- | --- | --- |
|  | **Characteristic** | **Chi-Square value** | **Degree of freedom** | ***P*-value** |
| **TCGA database** | Age | 1.0749 | 1 | 0.2998 |
|  | Gender | 1.1756 | 1 | 0.2783 |
|  | WHO grade | 1.9116 | 1 | 0.1668 |
|  | IDH status | 13.458 | 1 | 0.0002 |
|  | 1p/19q codeletion | 0.45584 | 1 | 0.4996 |
|  | SnG-Risk score | 23.937 | 1 | 9.95e-07 |
|  | Global | 30.376 | 6 | **3.33e-05** |
| If the *p*-value in the global test > 0.05, it indicates that the nomogram adheres to the PH assumption. | | | | |

| **Supplementary Table 18.** PH assumption test on the nomogram model proposed by Li et al.^36^ | | | | |
| --- | --- | --- | --- | --- |
|  | **Characteristic** | **Chi-Square value** | **Degree of freedom** | ***P*-value** |
| **TCGA database** | Age | 3.8954 | 1 | 0.0484 |
|  | Gender | 1.2523 | 1 | 0.2631 |
|  | WHO grade | 3.4526 | 1 | 0.0632 |
|  | IDH status | 13.225 | 1 | 0.0003 |
|  | 1p/19q codeletion | 0.60019 | 1 | 0.4385 |
|  | Risk score | 4.7263 | 1 | 0.0297 |
|  | Global | 19.182 | 6 | **0.0039** |
| If the *p*-value in the global test > 0.05, it indicates that the nomogram adheres to the PH assumption. | | | | |

| **Supplementary Table 19.** PH assumption test on the nomogram model proposed by Wang et al.^37^ | | | | |
| --- | --- | --- | --- | --- |
|  | **Characteristic** | **Chi-Square value** | **Degree of freedom** | ***P*-value** |
| **TCGA database** | Risk score | 7.2702 | 1 | 0.0070 |
|  | Cancer | 0.032726 | 1 | 0.8564 |
|  | Age | 3.9171 | 1 | 0.0478 |
|  | 1p/19q codeletion | 0.33528 | 1 | 0.5626 |
|  | Global | 10.67 | 4 | **0.0305** |
| If the *p*-value in the global test > 0.05, it indicates that the nomogram adheres to the PH assumption. | | | | |

| **Supplementary Table 20.** PH assumption test on the nomogram model proposed by Zhu et al.^38^ | | | | |
| --- | --- | --- | --- | --- |
|  | **Characteristic** | **Chi-Square value** | **Degree of freedom** | ***P*-value** |
| **TCGA database** | Gender | 0.0064581 | 1 | 0.9359 |
|  | WHO grade | 1.1663 | 1 | 0.2802 |
|  | Age | 7.6714 | 1 | 0.0056 |
|  | Risk score | 1.5434 | 1 | 0.2141 |
|  | Global | 11.344 | 4 | **0.0230** |
| If the *p*-value in the global test > 0.05, it indicates that the nomogram adheres to the PH assumption. | | | | |

| **Supplementary Table 21.** PH assumption test on the nomogram model proposed by Geng et al.^39^ | | | | |
| --- | --- | --- | --- | --- |
|  | **Characteristic** | **Chi-Square value** | **Degree of freedom** | ***P*-value** |
| **TCGA database (OS)** | IDH status | 13.647 | 1 | 0.0002 |
|  | Primary therapy outcome | 0.96204 | 1 | 0.3267 |
|  | Age | 3.3791 | 1 | 0.0660 |
|  | SIRPB1 | 4.2989 | 1 | 0.0381 |
|  | Global | 17.697 | 4 | **0.0014** |
| **TCGA database (PFI)** | IDH status | 0.56489 | 1 | 0.4523 |
|  | Primary therapy outcome | 0.95422 | 1 | 0.3286 |
|  | Age | 2.3495 | 1 | 0.1253 |
|  | SIRPB1 | 1.1869 | 1 | 0.2759 |
|  | Global | 4.6071 | 4 | 0.3300 |
| If the *p*-value in the global test > 0.05, it indicates that the nomogram adheres to the PH assumption. OS, overall survival; PFI, progression-free interval. | | | | |

| **Supplementary Table 22.** PH assumption test on the nomogram model proposed by Yu et al.^40^ | | | | |
| --- | --- | --- | --- | --- |
|  | **Characteristic** | **Chi-Square value** | **Degree of freedom** | ***P*-value** |
| **TCGA database** | CDRS risk score | 3.2805 | 1 | 0.0701 |
|  | Age | 3.0648 | 1 | 0.0800 |
|  | IDH status | 11.833 | 1 | 0.0006 |
|  | 1p/19q codeletion | 0.34381 | 1 | 0.5576 |
|  | WHO grade | 2.0732 | 2 | 0.3547 |
|  | MGMT | 5.6722 | 1 | 0.0172 |
|  | Global | 18.911 | 7 | **0.0085** |
| If the *p*-value in the global test > 0.05, it indicates that the nomogram adheres to the PH assumption. | | | | |

| **Supplementary Table 23.** PH assumption test on the nomogram model proposed by Zhou et al.^41^ | | | | |
| --- | --- | --- | --- | --- |
|  | **Characteristic** | **Chi-Square value** | **Degree of freedom** | ***P*-value** |
| **TCGA database** | Age | 7.9659 | 1 | 0.0048 |
|  | Risk score | 4.5772 | 1 | 0.0324 |
|  | Global | 13.429 | 2 | **0.0012** |
| If the *p*-value in the global test > 0.05, it indicates that the nomogram adheres to the PH assumption. | | | | |

| **Supplementary Table 24.** PH assumption test on the nomogram model proposed by Wu et al.^42^ | | | | |
| --- | --- | --- | --- | --- |
|  | **Characteristic** | **Chi-Square value** | **Degree of freedom** | ***P*-value** |
| **TCGA database** | Age | 2.5003 | 1 | 0.1138 |
|  | Gender | 1.2656 | 1 | 0.2606 |
|  | IDH status | 13.043 | 1 | 0.0003 |
|  | 1p/19q codeletion | 0.067368 | 1 | 0.7952 |
|  | WHO grade | 2.3233 | 2 | 0.3130 |
|  | Risk score | 2.2343 | 1 | 0.1350 |
|  | Global | 17.206 | 7 | **0.0161** |
| If the *p*-value in the global test > 0.05, it indicates that the nomogram adheres to the PH assumption. | | | | |

| **Supplementary Table 25.** PH assumption test on the nomogram model proposed by Peng et al.^43^ | | | | |
| --- | --- | --- | --- | --- |
|  | **Characteristic** | **Chi-Square value** | **Degree of freedom** | ***P*-value** |
| **CGGA693 database** | Age | 0.48333 | 1 | 0.4869 |
|  | WHO grade | 11.744 | 2 | 0.0028 |
|  | GA-MSCRGPI | 7.8611 | 1 | 0.0051 |
|  | Global | 16.115 | 4 | **0.0029** |
| **CGGA325 database** | Age | 1.1891 | 1 | 0.2755 |
|  | WHO grade | 18.327 | 2 | 0.0001 |
|  | GA-MSCRGPI | 0.08639 | 1 | 0.7688 |
|  | Global | 22.01 | 4 | **0.0002** |
| **TCGA database** | Age | 3.8483 | 1 | 0.0498 |
|  | WHO grade | 0.90991 | 2 | 0.6345 |
|  | GA-MSCRGPI | 0.59626 | 1 | 0.4400 |
|  | Global | 5.6127 | 4 | 0.2300 |
| If the *p*-value in the global test > 0.05, it indicates that the nomogram adheres to the PH assumption. | | | | |

| **Supplementary Table 26.** PH assumption test on the nomogram model proposed by Wang et al.^44^ | | | | |
| --- | --- | --- | --- | --- |
|  | **Characteristic** | **Chi-Square value** | **Degree of freedom** | ***P*-value** |
| **TCGA database** | Age | 2.529 | 1 | 0.1118 |
|  | Risk score | 3.7068 | 1 | 0.0542 |
|  | Global | 6.1753 | 2 | **0.0456** |
| If the *p*-value in the global test > 0.05, it indicates that the nomogram adheres to the PH assumption. | | | | |

| **Supplementary Table 27.** PH assumption test on the nomogram model proposed by Lin et al.^11^ | | | | |
| --- | --- | --- | --- | --- |
|  | **Characteristic** | **Chi-Square value** | **Degree of freedom** | ***P*-value** |
| **CGGA database** | Age | 0.090421 | 1 | 0.7636 |
|  | PRS type | 12.387 | 1 | 0.0004 |
|  | WHO grade | 17.199 | 2 | 0.0002 |
|  | Radiotherapy status | 8.5671 | 1 | 0.0034 |
|  | Chemotherapy status | 0.48496 | 1 | 0.4862 |
|  | IDH status | 18.545 | 1 | 1.66e-05 |
|  | 1p/19q codeletion | 10.071 | 1 | 0.0015 |
|  | VMP1 | 4.1226 | 1 | 0.0423 |
|  | Global | 57.444 | 9 | **4.15e-09** |
| If the *p*-value in the global test > 0.05, it indicates that the nomogram adheres to the PH assumption. | | | | |

| **Supplementary Table 28.** PH assumption test on the nomogram model proposed by Han et al.^10^ | | | | |
| --- | --- | --- | --- | --- |
|  | **Characteristic** | **Chi-Square value** | **Degree of freedom** | ***P*-value** |
| **CGGA database** | WHO grade | 13.461 | 1 | 0.0002 |
|  | Sex | 5.0072 | 1 | 0.0252 |
|  | Age at diagnosis | 2.5224 | 1 | 0.1122 |
|  | IDH status | 0.69489 | 1 | 0.4045 |
|  | Radiotherapy status | 6.0283 | 1 | 0.0141 |
|  | Chemotherapy status | 2.7208 | 1 | 0.0990 |
|  | HVEM level | 0.00012854 | 1 | 0.9910 |
|  | Global | 39.423 | 7 | **1.62e-06** |
| If the *p*-value in the global test > 0.05, it indicates that the nomogram adheres to the PH assumption. | | | | |

| **Supplementary Table 29.** PH assumption test on the nomogram model proposed by Liu et al.^45^ | | | | |
| --- | --- | --- | --- | --- |
|  | **Characteristic** | **Chi-Square value** | **Degree of freedom** | ***P*-value** |
| **TCGA database** | Age | 6.5376 | 1 | 0.0106 |
|  | IDH status | 18.811 | 1 | 1.44e-05 |
|  | WHO grade | 8.6928 | 1 | 0.0032 |
|  | ATRX status | 0.01652 | 1 | 0.8977 |
|  | Subtype | 2.0456 | 1 | 0.1526 |
|  | Global | 26.932 | 5 | **5.88e-05** |
| If the *p*-value in the global test > 0.05, it indicates that the nomogram adheres to the PH assumption. | | | | |

| **Supplementary Table 30.** PH assumption test on the nomogram model proposed by He et al.^46^ | | | | |
| --- | --- | --- | --- | --- |
|  | **Characteristic** | **Chi-Square value** | **Degree of freedom** | ***P*-value** |
| **TCGA database** | Age | 3.8211 | 1 | 0.0506 |
|  | Gender | 0.40145 | 1 | 0.5263 |
|  | Type | 0.01323 | 1 | 0.9084 |
|  | Risk score | 0.037215 | 1 | 0.8470 |
|  | Global | 5.0403 | 4 | 0.2832 |
| If the *p*-value in the global test > 0.05, it indicates that the nomogram adheres to the PH assumption. | | | | |

| **Supplementary Table 31.** PH assumption test on the nomogram model proposed by Zeng et al.^24^ | | | | |
| --- | --- | --- | --- | --- |
|  | **Characteristic** | **Chi-Square value** | **Degree of freedom** | ***P*-value** |
| **TCGA database** | Age | 3.6832 | 1 | 0.0550 |
|  | Gender | 0.017351 | 1 | 0.8952 |
|  | WHO grade | 1.5072 | 2 | 0.4707 |
|  | Risk score | 0.00011956 | 1 | 0.9913 |
|  | Global | 6.4335 | 5 | 0.2663 |
| If the *p*-value in the global test > 0.05, it indicates that the nomogram adheres to the PH assumption. | | | | |

| **Supplementary Table 32.** PH assumption test on the nomogram model proposed by Zhang et al.^47^ | | | | |
| --- | --- | --- | --- | --- |
|  | **Characteristic** | **Chi-Square value** | **Degree of freedom** | ***P*-value** |
| **TCGA database** | Age | 5.1444 | 1 | 0.0233 |
|  | 1p/19q codeletion | 0.044504 | 1 | 0.8329 |
|  | Histological type | 2.0678 | 1 | 0.1504 |
|  | Primary therapy outcome | 1.6982 | 1 | 0.1925 |
|  | TGIF2 | 0.84994 | 1 | 0.3566 |
|  | Global | 9.9005 | 5 | 0.0781 |
| If the *p*-value in the global test > 0.05, it indicates that the nomogram adheres to the PH assumption. | | | | |

| **Supplementary Table 33.** PH assumption test on the nomogram model proposed by Zhao et al.^48^ | | | | |
| --- | --- | --- | --- | --- |
|  | **Characteristic** | **Chi-Square value** | **Degree of freedom** | ***P*-value** |
| **TCGA database** | Gender | 0.15641 | 1 | 0.6925 |
|  | Age | 1.08 | 1 | 0.2987 |
|  | IDH status | 0.67318 | 1 | 0.4119 |
|  | Risk score | 1.2174 | 1 | 0.2699 |
|  | Global | 2.6878 | 4 | 0.6113 |
| If the *p*-value in the global test > 0.05, it indicates that the nomogram adheres to the PH assumption. | | | | |

| **Supplementary Table 34.** PH assumption test on the nomogram model proposed by Wang et al.^49^ | | | | |
| --- | --- | --- | --- | --- |
|  | **Characteristic** | **Chi-Square value** | **Degree of freedom** | ***P*-value** |
| **CGGA301 database** | ARL3 | 0.071385 | 1 | 0.7893 |
|  | Age | 0.060621 | 1 | 0.8055 |
|  | WHO grade | 9.0828 | 2 | 0.0107 |
|  | IDH status | 0.10086 | 1 | 0.7508 |
|  | Gender | 0.017206 | 1 | 0.8956 |
|  | Global | 12.274 | 6 | 0.0561 |
| **Gravendeel database** | ARL3 | 0.47253 | 1 | 0.4918 |
|  | Age | 0.082463 | 1 | 0.7740 |
|  | WHO grade | 2.8943 | 3 | 0.4082 |
|  | IDH status | 0.032333 | 1 | 0.8573 |
|  | Gender | 0.8433 | 1 | 0.3585 |
|  | Global | 7.2752 | 7 | 0.4008 |
| **TCGA database** | ARL3 | 4.4963 | 1 | 0.0340 |
|  | Age | 9.8635 | 1 | 0.0017 |
|  | WHO grade | 5.5109 | 2 | 0.0636 |
|  | IDH status | 19.412 | 1 | 1.05e-05 |
|  | Gender | 0.58016 | 1 | 0.4463 |
|  | Global | 24.848 | 6 | **0.0004** |
| If the *p*-value in the global test > 0.05, it indicates that the nomogram adheres to the PH assumption. | | | | |

| **Supplementary Table 35.** PH assumption test on the nomogram model proposed by Song et al.^17^ | | | | |
| --- | --- | --- | --- | --- |
|  | **Characteristic** | **Chi-Square value** | **Degree of freedom** | ***P*-value** |
| **TCGA database** | Age | 2.1988 | 1 | 0.1381 |
|  | WHO grade | 9.4892e-05 | 1 | 0.9922 |
|  | Risk score | 4.0677 | 1 | 0.0437 |
|  | Global | 6.4765 | 3 | 0.0906 |
| If the *p*-value in the global test > 0.05, it indicates that the nomogram adheres to the PH assumption. | | | | |

| **Supplementary Table 36.** PH assumption test on the nomogram model proposed by Dai et al.^50^ | | | | |
| --- | --- | --- | --- | --- |
|  | **Characteristic** | **Chi-Square value** | **Degree of freedom** | ***P*-value** |
| **TCGA database (OS)** | WHO grade | 5.924 | 2 | 0.0517 |
|  | PDCD2 | 0.34806 | 1 | 0.5552 |
|  | Global | 7.0896 | 3 | 0.0691 |
| **TCGA database (DSS)** | WHO grade | 3.6542 | 2 | 0.1609 |
|  | PDCD2 | 0.66838 | 1 | 0.4136 |
|  | Global | 5.0577 | 3 | 0.1676 |
| **TCGA database (PFI)** | WHO grade | 9.5978 | 2 | 0.0082 |
|  | PDCD2 | 0.59853 | 1 | 0.4391 |
|  | Global | 9.6841 | 3 | **0.0215** |
| If the *p*-value in the global test > 0.05, it indicates that the nomogram adheres to the PH assumption. OS, overall survival; DSS, disease-specific survival; PFI, progression-free interval. | | | | |

| **Supplementary Table 37.** PH assumption test on the nomogram model proposed by Xie et al.^51^ | | | | |
| --- | --- | --- | --- | --- |
|  | **Characteristic** | **Chi-Square value** | **Degree of freedom** | ***P*-value** |
| **TCGA database** | Race | 0.00095515 | 1 | 0.9753 |
|  | Age | 2.8288 | 1 | 0.0926 |
|  | MFF | 0.0020107 | 1 | 0.9642 |
|  | MSTO1 | 0.006712 | 1 | 0.9347 |
|  | MFN1 | 0.16779 | 1 | 0.6821 |
|  | MIEF1 | 0.67885 | 1 | 0.4100 |
|  | MIEF2 | 0.36694 | 1 | 0.5447 |
|  | Gender | 0.60072 | 1 | 0.4383 |
|  | Global | 7.1877 | 8 | 0.5165 |
| If the *p*-value in the global test > 0.05, it indicates that the nomogram adheres to the PH assumption. | | | | |

| **Supplementary Table 38.** PH assumption test on the nomogram model proposed by Zeng et al.^14^ | | | | |
| --- | --- | --- | --- | --- |
|  | **Characteristic** | **Chi-Square value** | **Degree of freedom** | ***P*-value** |
| **TCGA database** | WHO grade | 1.7522 | 2 | 0.4164 |
|  | Age | 3.7647 | 1 | 0.0523 |
|  | Risk score | 0.00085029 | 1 | 0.9767 |
|  | Global | 6.6244 | 4 | 0.1571 |
| If the *p*-value in the global test > 0.05, it indicates that the nomogram adheres to the PH assumption. | | | | |

| **Supplementary Table 39.** PH assumption test on the nomogram model proposed by Wang et al.^52^ | | | | |
| --- | --- | --- | --- | --- |
|  | **Characteristic** | **Chi-Square value** | **Degree of freedom** | ***P*-value** |
| **TCGA database** | Risk score | 0.0094967 | 1 | 0.9224 |
|  | Age | 2.9722 | 1 | 0.0847 |
|  | 1p/19q codeletion | 0.13358 | 1 | 0.7148 |
|  | Global | 3.0924 | 3 | 0.3776 |
| **CGGA325 database** | Risk score | 0.80064 | 1 | 0.3709 |
|  | Age | 2.6167 | 1 | 0.1057 |
|  | 1p/19q codeletion | 0.84426 | 1 | 0.3582 |
|  | Global | 4.9045 | 3 | 0.1789 |
| If the *p*-value in the global test > 0.05, it indicates that the nomogram adheres to the PH assumption. | | | | |

| **Supplementary Table 40.** Metadata of patients in TCGA-LGG cohort acquired from Liu et al.^45^ | | | | | | | | | | | | | | | |
| --- | --- | --- | --- | --- | --- | --- | --- | --- | --- | --- | --- | --- | --- | --- | --- |
| **ID** | **OS time (Day)** | **OS** | **Subtype** | **Gender** | **Histological type*** | **Age** | **Laterality** | **Grade** | **IDH status** | **X1p 19q codeletion** | **MGMT promoter status** | **TERT promoter status** | **ATRX status** | **Primary therapy response** | **Followup treatment response** |
| TCGA-CS-4938 | 3574 | 0 | 2 | Female | A | 31 | Right | G2 | Mutant | non-codel | Unmethylated | WT | Mutant | NR | NR |
| TCGA-CS-4941 | 234 | 1 | 2 | Male | A | 67 | Right | G3 | WT | non-codel | Methylated | Mutant | WT | NR | NR |
| TCGA-CS-4942 | 1335 | 1 | 2 | Female | A | 44 | Right | G3 | Mutant | non-codel | Unmethylated | WT | Mutant | NR | NR |
| TCGA-CS-4943 | 1106 | 1 | 1 | Male | A | 37 | Left | G3 | Mutant | non-codel | Methylated | WT | Mutant | NA | NA |
| TCGA-CS-4944 | 1828 | 0 | 1 | Male | A | 50 | Right | G2 | Mutant | non-codel | Methylated | Mutant | WT | NR | NR |
| TCGA-CS-5393 | 1222 | 0 | 2 | Male | A | 39 | Left | G3 | Mutant | non-codel | Methylated | WT | WT | R | R |
| TCGA-CS-5394 | 8 | 0 | 2 | Male | A | 40 | Left | G3 | Mutant | non-codel | Methylated | WT | WT | NA | NA |
| TCGA-CS-5395 | 639 | 1 | 2 | Male | O | 43 | Right | G2 | WT | non-codel | Unmethylated | Mutant | WT | NR | NR |
| TCGA-CS-5396 | 1631 | 0 | 1 | Female | O | 53 | Right | G3 | Mutant | codel | Methylated | Mutant | WT | R | NR |
| TCGA-CS-5397 | 194 | 1 | 2 | Female | A | 54 | Left | G3 | WT | non-codel | Unmethylated | Mutant | WT | NR | NA |
| TCGA-CS-6186 | 538 | 1 | 1 | Male | OA | 58 | Right | G3 | WT | non-codel | Unmethylated | Mutant | WT | NR | NR |
| TCGA-CS-6188 | 814 | 1 | 1 | Male | A | 48 | Right | G3 | WT | non-codel | Unmethylated | Mutant | WT | NR | NA |
| TCGA-CS-6290 | 1137 | 1 | 2 | Male | A | 31 | Left | G3 | Mutant | non-codel | Methylated | WT | WT | NR | NA |
| TCGA-CS-6665 | 1568 | 0 | 2 | Female | A | 51 | Right | G3 | Mutant | non-codel | Methylated | WT | WT | NR | NR |
| TCGA-CS-6666 | 1428 | 0 | 2 | Male | A | 22 | Right | G3 | Mutant | non-codel | Methylated | WT | Mutant | R | R |
| TCGA-CS-6667 | 1469 | 0 | 2 | Female | A | 39 | Left | G2 | Mutant | non-codel | Methylated | WT | WT | NR | NR |
| TCGA-CS-6668 | 1519 | 0 | 2 | Female | O | 57 | Left | G2 | Mutant | codel | Methylated | Mutant | WT | NR | NR |
| TCGA-CS-6669 | 1494 | 0 | 1 | Female | O | 26 | Right | G2 | WT | non-codel | Unmethylated | WT | WT | NR | NR |
| TCGA-CS-6670 | 1426 | 0 | 1 | Male | O | 43 | Right | G3 | Mutant | codel | Methylated | NA | WT | NA | NR |
| TCGA-DB-5270 | 3733 | 0 | 1 | Female | OA | 38 | Left | G3 | Mutant | non-codel | Methylated | NA | Mutant | R | R |
| TCGA-DB-5273 | 2493 | 0 | 1 | Male | A | 33 | Right | G3 | Mutant | non-codel | Unmethylated | WT | Mutant | R | R |
| TCGA-DB-5274 | 2289 | 0 | 1 | Female | OA | 37 | Left | G3 | Mutant | codel | Methylated | Mutant | WT | R | R |
| TCGA-DB-5275 | 1458 | 0 | 1 | Male | OA | 36 | Right | G3 | Mutant | non-codel | Methylated | WT | Mutant | R | NA |
| TCGA-DB-5276 | 2218 | 0 | 1 | Male | OA | 32 | Left | G3 | Mutant | non-codel | Methylated | WT | Mutant | R | R |
| TCGA-DB-5277 | 1547 | 1 | 1 | Male | A | 34 | Left | G3 | Mutant | non-codel | Methylated | WT | WT | R | NR |
| TCGA-DB-5278 | 2772 | 0 | 1 | Male | O | 17 | Left | G2 | Mutant | codel | Methylated | WT | WT | NR | NR |
| TCGA-DB-5279 | 1354 | 0 | 1 | Male | O | 59 | Right | G2 | Mutant | codel | Methylated | Mutant | WT | R | R |
| TCGA-DB-5280 | 1112 | 0 | 1 | Male | OA | 43 | Right | G2 | Mutant | non-codel | Methylated | WT | Mutant | R | R |
| TCGA-DB-5281 | 2381 | 0 | 1 | Male | OA | 61 | Left | G3 | Mutant | non-codel | Methylated | WT | Mutant | NR | NR |
| TCGA-DB-A4X9 | 1412 | 0 | 1 | Female | OA | 33 | Left | G2 | Mutant | non-codel | Methylated | WT | WT | R | R |
| TCGA-DB-A4XA | 573 | 0 | 1 | Male | OA | 30 | Left | G2 | Mutant | codel | Methylated | Mutant | WT | NR | NA |
| TCGA-DB-A4XB | 919 | 0 | 1 | Male | A | 38 | Left | G3 | Mutant | non-codel | Methylated | WT | WT | NR | R |
| TCGA-DB-A4XC | 1421 | 0 | 1 | Male | OA | 26 | Left | G2 | Mutant | non-codel | Methylated | WT | Mutant | R | NR |
| TCGA-DB-A4XD | 1210 | 0 | 1 | Male | A | 32 | Right | G3 | Mutant | non-codel | Methylated | WT | Mutant | R | R |
| TCGA-DB-A4XE | 1139 | 0 | 1 | Female | OA | 27 | Left | G3 | Mutant | non-codel | Methylated | WT | Mutant | R | R |
| TCGA-DB-A4XF | 1021 | 0 | 1 | Female | A | 41 | Right | G3 | Mutant | non-codel | Methylated | WT | WT | NR | R |
| TCGA-DB-A4XG | 2219 | 0 | 1 | Male | O | 34 | Right | G3 | Mutant | codel | Methylated | Mutant | WT | NR | NA |
| TCGA-DB-A4XH | 962 | 0 | 1 | Female | OA | 53 | Left | G2 | Mutant | codel | Methylated | Mutant | WT | NR | R |
| TCGA-DB-A64L | 736 | 0 | 1 | Female | O | 67 | Right | G2 | Mutant | codel | Methylated | Mutant | WT | R | NR |
| TCGA-DB-A64O | 775 | 1 | 1 | Male | OA | 59 | Left | G2 | WT | non-codel | Unmethylated | Mutant | WT | NR | NA |
| TCGA-DB-A64P | 916 | 0 | 1 | Male | O | 40 | Left | G3 | Mutant | codel | Methylated | Mutant | WT | R | R |
| TCGA-DB-A64Q | 182 | 0 | 1 | Female | OA | 31 | Left | G2 | Mutant | codel | Methylated | Mutant | WT | NR | NA |
| TCGA-DB-A64R | 955 | 0 | 1 | Female | O | 24 | Right | G2 | Mutant | codel | Methylated | Mutant | WT | R | R |
| TCGA-DB-A64S | 835 | 0 | 1 | Male | OA | 20 | NA | G2 | Mutant | non-codel | Unmethylated | WT | Mutant | R | R |
| TCGA-DB-A64U | 846 | 0 | 1 | Female | OA | 38 | Right | G2 | Mutant | codel | Methylated | Mutant | WT | R | NA |
| TCGA-DB-A64V | 889 | 0 | 1 | Male | O | 54 | Left | G2 | Mutant | codel | Methylated | Mutant | WT | NR | R |
| TCGA-DB-A64W | 438 | 1 | 1 | Female | OA | 65 | Right | G3 | Mutant | codel | Methylated | Mutant | WT | NR | NR |
| TCGA-DB-A64X | 563 | 0 | 1 | Female | A | 56 | Right | G3 | Mutant | non-codel | Methylated | WT | WT | NR | R |
| TCGA-DB-A75K | 368 | 0 | 1 | Female | OA | 55 | Right | G3 | Mutant | codel | Methylated | NA | WT | R | R |
| TCGA-DB-A75L | 342 | 0 | 1 | Female | A | 36 | Left | G3 | Mutant | non-codel | Methylated | NA | WT | R | R |
| TCGA-DB-A75M | 544 | 0 | 1 | Male | A | 47 | Right | G2 | Mutant | non-codel | Methylated | NA | Mutant | R | R |
| TCGA-DB-A75O | 935 | 0 | 1 | Male | A | 29 | Left | G3 | Mutant | non-codel | Methylated | NA | WT | R | NA |
| TCGA-DB-A75P | 492 | 0 | 1 | Female | A | 25 | Right | G2 | WT | non-codel | Unmethylated | NA | WT | R | R |
| TCGA-DH-5140 | 607 | 1 | 1 | Female | OA | 38 | Left | G3 | WT | non-codel | Unmethylated | WT | WT | NR | NR |
| TCGA-DH-5141 | 968 | 0 | 1 | Male | O | 32 | Left | G3 | Mutant | codel | Methylated | Mutant | WT | NR | NA |
| TCGA-DH-5142 | 1943 | 0 | 1 | Male | A | 29 | Left | G3 | Mutant | non-codel | Methylated | WT | Mutant | NR | NR |
| TCGA-DH-5143 | 1401 | 0 | 1 | Male | OA | 30 | Left | G3 | Mutant | non-codel | Methylated | WT | WT | NR | NR |
| TCGA-DH-5144 | 748 | 0 | 1 | Female | O | 56 | Right | G3 | Mutant | codel | Methylated | Mutant | Mutant | R | NR |
| TCGA-DH-A669 | 919 | 1 | 1 | Male | O | 70 | Left | G3 | Mutant | codel | Methylated | Mutant | WT | NA | NA |
| TCGA-DH-A66B | 1279 | 0 | 1 | Male | A | 52 | Left | G3 | Mutant | non-codel | Methylated | WT | WT | NR | NR |
| TCGA-DH-A66D | 846 | 0 | 1 | Female | A | 43 | Right | G3 | Mutant | non-codel | Methylated | NA | Mutant | NR | NR |
| TCGA-DH-A66F | 523 | 0 | 1 | Male | O | 49 | Left | G2 | Mutant | codel | Methylated | Mutant | WT | R | R |
| TCGA-DH-A66G | 523 | 0 | 1 | Female | O | 49 | Left | G3 | Mutant | non-codel | Methylated | NA | Mutant | NR | R |
| TCGA-DH-A7UR | 1650 | 0 | 1 | Female | O | 59 | Right | G3 | Mutant | codel | Methylated | NA | WT | R | NA |
| TCGA-DH-A7US | 706 | 0 | 1 | Male | O | 50 | Right | G2 | Mutant | codel | Methylated | NA | WT | NR | NR |
| TCGA-DH-A7UT | 531 | 1 | 1 | Male | A | 30 | Left | G3 | Mutant | non-codel | Methylated | NA | WT | NR | NR |
| TCGA-DH-A7UU | 417 | 0 | 1 | Male | A | 43 | Right | G3 | Mutant | non-codel | Methylated | NA | WT | NR | NR |
| TCGA-DH-A7UV | 566 | 0 | 1 | Male | A | 49 | Right | G3 | Mutant | non-codel | Methylated | NA | Mutant | NR | NR |
| TCGA-DU-5847 | 548 | 0 | 2 | Female | A | 34 | Right | G3 | WT | non-codel | Methylated | Mutant | WT | NR | NA |
| TCGA-DU-5849 | 443 | 0 | 2 | Male | O | 48 | Left | G2 | Mutant | codel | Methylated | Mutant | WT | NR | NA |
| TCGA-DU-5851 | 531 | 0 | 2 | Female | OA | 40 | Right | G3 | Mutant | non-codel | Unmethylated | WT | Mutant | NR | NA |
| TCGA-DU-5852 | 205 | 1 | 2 | Female | OA | 61 | Left | G3 | WT | non-codel | Methylated | Mutant | Mutant | NR | NR |
| TCGA-DU-5853 | 407 | 0 | 2 | Male | OA | 29 | Left | G2 | Mutant | non-codel | Methylated | WT | Mutant | NR | NA |
| TCGA-DU-5854 | 257 | 0 | 2 | Female | A | 57 | Right | G3 | WT | non-codel | Unmethylated | Mutant | WT | NR | NA |
| TCGA-DU-5855 | 207 | 0 | 2 | Female | OA | 49 | Right | G3 | Mutant | non-codel | Methylated | WT | Mutant | NR | NA |
| TCGA-DU-5870 | 5546 | 0 | 1 | Female | O | 34 | Right | G2 | Mutant | codel | Methylated | Mutant | WT | NR | NR |
| TCGA-DU-5871 | 576 | 0 | 2 | Female | OA | 37 | Left | G2 | Mutant | non-codel | Methylated | WT | Mutant | NR | NA |
| TCGA-DU-5872 | 532 | 0 | 2 | Female | OA | 43 | Left | G2 | Mutant | non-codel | Methylated | WT | Mutant | NR | NR |
| TCGA-DU-5874 | 461 | 0 | 2 | Female | O | 62 | Left | G2 | Mutant | codel | Methylated | Mutant | WT | NR | NA |
| TCGA-DU-6392 | 6423 | 0 | 1 | Female | A | 35 | Right | G3 | WT | non-codel | Unmethylated | NA | Mutant | NR | NR |
| TCGA-DU-6393 | 1585 | 1 | 2 | Male | O | 66 | Left | G3 | Mutant | codel | Methylated | Mutant | WT | NA | NA |
| TCGA-DU-6394 | 682 | 1 | 2 | Male | O | 53 | Midline | G3 | Mutant | codel | Methylated | Mutant | WT | NR | NR |
| TCGA-DU-6395 | 1491 | 1 | 2 | Male | OA | 31 | Left | G2 | Mutant | non-codel | Methylated | NA | Mutant | NR | NR |
| TCGA-DU-6396 | 2286 | 1 | 2 | Female | OA | 31 | Right | G3 | Mutant | non-codel | Methylated | WT | Mutant | NR | NR |
| TCGA-DU-6397 | 1401 | 1 | 2 | Male | O | 45 | Right | G3 | Mutant | codel | Methylated | Mutant | WT | NR | NR |
| TCGA-DU-6399 | 2000 | 1 | 2 | Male | O | 54 | Right | G2 | Mutant | non-codel | Methylated | WT | Mutant | NR | NR |
| TCGA-DU-6400 | 37 | 1 | 2 | Female | O | 66 | Left | G2 | Mutant | codel | Methylated | Mutant | WT | NA | NA |
| TCGA-DU-6401 | 2660 | 1 | 2 | Female | O | 31 | Left | G2 | Mutant | non-codel | Methylated | WT | Mutant | NR | NR |
| TCGA-DU-6402 | 214 | 1 | 2 | Male | A | 52 | Right | G3 | WT | non-codel | Unmethylated | Mutant | WT | NR | NR |
| TCGA-DU-6403 | 354 | 1 | 2 | Female | OA | 60 | Right | G3 | WT | non-codel | Unmethylated | Mutant | WT | NA | NA |
| TCGA-DU-6404 | 4068 | 1 | 2 | Female | O | 24 | Right | G3 | WT | non-codel | Unmethylated | WT | WT | NR | NR |
| TCGA-DU-6405 | 605 | 1 | 2 | Female | A | 51 | Right | G3 | WT | non-codel | Methylated | Mutant | WT | NR | NR |
| TCGA-DU-6406 | 512 | 1 | 2 | Female | OA | 59 | Left | G3 | WT | non-codel | Unmethylated | NA | WT | NA | NA |
| TCGA-DU-6407 | 2875 | 1 | 2 | Female | O | 35 | Right | G2 | Mutant | non-codel | Methylated | WT | Mutant | NR | NR |
| TCGA-DU-6408 | 3470 | 1 | 2 | Female | O | 23 | Right | G3 | Mutant | non-codel | Methylated | WT | Mutant | NR | NR |
| TCGA-DU-6410 | 242 | 0 | 2 | Male | O | 56 | Midline | G3 | Mutant | codel | Methylated | Mutant | WT | NA | NA |
| TCGA-DU-6542 | 73 | 0 | 2 | Male | OA | 25 | Right | G3 | Mutant | non-codel | Methylated | WT | WT | NR | NA |
| TCGA-DU-7006 | 349 | 1 | 1 | Female | A | 60 | Left | G3 | WT | non-codel | Methylated | Mutant | WT | NR | NA |
| TCGA-DU-7007 | 1915 | 1 | 1 | Male | A | 33 | Right | G2 | Mutant | non-codel | Methylated | WT | Mutant | NA | NA |
| TCGA-DU-7008 | 4752 | 0 | 1 | Female | O | 41 | Right | G2 | Mutant | non-codel | Methylated | WT | Mutant | NR | NR |
| TCGA-DU-7009 | 4695 | 1 | 1 | Female | O | 32 | Left | G2 | Mutant | codel | Methylated | Mutant | WT | NR | NR |
| TCGA-DU-7010 | 456 | 1 | 2 | Female | A | 58 | Left | G3 | Mutant | non-codel | Methylated | WT | WT | NR | NR |
| TCGA-DU-7011 | 3200 | 1 | 1 | Male | OA | 25 | Right | G2 | Mutant | non-codel | Unmethylated | NA | Mutant | NR | NA |
| TCGA-DU-7012 | 199 | 1 | 1 | Female | A | 74 | Left | G3 | WT | non-codel | Methylated | Mutant | WT | NR | NR |
| TCGA-DU-7013 | 269 | 1 | 1 | Male | A | 59 | Left | G3 | WT | non-codel | Unmethylated | Mutant | WT | NR | NR |
| TCGA-DU-7014 | 3571 | 1 | 1 | Male | O | 59 | Right | G2 | NA | non-codel | Methylated | NA | NA | NR | NR |
| TCGA-DU-7015 | 2761 | 0 | 1 | Female | O | 41 | Right | G2 | Mutant | non-codel | Methylated | WT | Mutant | NR | NR |
| TCGA-DU-7018 | 933 | 1 | 2 | Female | O | 57 | Right | G3 | Mutant | codel | Methylated | Mutant | WT | NR | NR |
| TCGA-DU-7019 | 800 | 0 | 1 | Male | OA | 39 | Right | NA | Mutant | non-codel | Methylated | WT | WT | NR | NA |
| TCGA-DU-7290 | 315 | 1 | 1 | Female | A | 45 | Right | G3 | WT | non-codel | Unmethylated | Mutant | WT | NA | NA |
| TCGA-DU-7292 | 242 | 1 | 1 | Male | A | 69 | Left | G3 | WT | non-codel | Methylated | WT | WT | NR | NR |
| TCGA-DU-7294 | 2869 | 0 | 1 | Female | O | 53 | Right | G2 | Mutant | codel | Methylated | Mutant | WT | NR | NR |
| TCGA-DU-7298 | 576 | 1 | 1 | Female | A | 38 | Left | G3 | Mutant | non-codel | Methylated | WT | Mutant | NR | NA |
| TCGA-DU-7299 | 1339 | 1 | 1 | Male | A | 33 | Left | G3 | Mutant | non-codel | Methylated | WT | WT | NR | NR |
| TCGA-DU-7300 | 1886 | 1 | 1 | Female | O | 53 | Right | G3 | Mutant | codel | Methylated | Mutant | WT | NR | NR |
| TCGA-DU-7301 | 788 | 1 | 1 | Male | O | 53 | Left | G2 | Mutant | non-codel | Methylated | WT | Mutant | NR | NR |
| TCGA-DU-7302 | 1834 | 0 | 1 | Female | O | 48 | Left | G3 | Mutant | codel | Methylated | Mutant | WT | NR | NR |
| TCGA-DU-7304 | 709 | 1 | 1 | Male | OA | 43 | Left | G3 | Mutant | non-codel | Methylated | WT | Mutant | NR | NR |
| TCGA-DU-7306 | 1277 | 0 | 1 | Male | OA | 67 | Right | G2 | Mutant | non-codel | Methylated | WT | Mutant | NR | NA |
| TCGA-DU-7309 | 84 | 0 | 1 | Female | O | 41 | Left | G3 | Mutant | non-codel | Methylated | WT | Mutant | NR | NA |
| TCGA-DU-8158 | 155 | 1 | 2 | Female | A | 57 | Left | G3 | WT | non-codel | Unmethylated | Mutant | WT | NA | NA |
| TCGA-DU-8161 | 722 | 1 | 2 | Female | OA | 63 | Left | G3 | WT | non-codel | Unmethylated | Mutant | WT | NR | NR |
| TCGA-DU-8162 | 444 | 1 | 2 | Female | OA | 61 | Right | G3 | WT | non-codel | Unmethylated | WT | WT | NR | NA |
| TCGA-DU-8163 | 629 | 0 | 2 | Male | OA | 29 | Left | G3 | Mutant | non-codel | Unmethylated | WT | Mutant | NR | NA |
| TCGA-DU-8164 | 651 | 0 | 2 | Male | O | 51 | Right | G2 | Mutant | codel | Methylated | Mutant | WT | NR | NR |
| TCGA-DU-8165 | 582 | 0 | 2 | Female | O | 60 | Left | G3 | WT | non-codel | Unmethylated | Mutant | WT | NR | NR |
| TCGA-DU-8166 | 516 | 0 | 2 | Female | OA | 29 | Left | G2 | Mutant | non-codel | Methylated | WT | Mutant | NR | NR |
| TCGA-DU-8167 | 471 | 0 | 2 | Female | OA | 69 | Right | G2 | Mutant | non-codel | Methylated | WT | WT | NR | NR |
| TCGA-DU-8168 | 431 | 0 | 2 | Female | O | 55 | Right | G3 | Mutant | codel | Methylated | Mutant | WT | NR | NR |
| TCGA-DU-A5TP | 1012 | 0 | 1 | Male | A | 33 | Right | G3 | Mutant | non-codel | Methylated | WT | Mutant | NA | NR |
| TCGA-DU-A5TR | 908 | 0 | 1 | Male | OA | 51 | Left | G2 | Mutant | non-codel | Methylated | WT | Mutant | NA | NR |
| TCGA-DU-A5TS | 964 | 0 | 1 | Male | O | 42 | Right | G2 | Mutant | non-codel | Methylated | WT | Mutant | NR | NR |
| TCGA-DU-A5TT | 743 | 0 | 1 | Male | O | 70 | Left | G3 | WT | non-codel | Methylated | Mutant | WT | NA | NR |
| TCGA-DU-A5TU | 796 | 0 | 1 | Female | A | 62 | Left | G2 | Mutant | non-codel | Methylated | WT | Mutant | NR | NR |
| TCGA-DU-A5TW | 760 | 0 | 1 | Female | A | 33 | Right | G3 | Mutant | non-codel | Methylated | WT | Mutant | NR | NR |
| TCGA-DU-A5TY | 1033 | 1 | 1 | Female | A | 46 | Left | G3 | WT | non-codel | Methylated | Mutant | WT | NA | NR |
| TCGA-DU-A6S2 | 777 | 0 | 1 | Female | O | 37 | Right | G2 | Mutant | codel | Methylated | NA | WT | NR | NR |
| TCGA-DU-A6S3 | 656 | 0 | 1 | Male | O | 60 | Right | G2 | Mutant | codel | Methylated | NA | WT | NR | NA |
| TCGA-DU-A6S6 | 2893 | 0 | 1 | Female | OA | 35 | Right | G2 | Mutant | codel | Methylated | NA | WT | NA | NR |
| TCGA-DU-A6S7 | 638 | 0 | 1 | Female | A | 27 | Left | G3 | Mutant | non-codel | Methylated | NA | Mutant | NR | NR |
| TCGA-DU-A6S8 | 678 | 0 | 2 | Female | O | 74 | Right | G3 | Mutant | codel | Methylated | NA | WT | NR | NA |
| TCGA-DU-A76K | 347 | 1 | 1 | Male | O | 87 | Right | G2 | WT | non-codel | Unmethylated | NA | WT | NA | NA |
| TCGA-DU-A76L | 814 | 1 | 1 | Male | O | 54 | Right | G3 | WT | non-codel | Methylated | NA | WT | NA | NA |
| TCGA-DU-A76O | 522 | 0 | 1 | Male | A | 30 | Left | G2 | Mutant | non-codel | Methylated | NA | Mutant | NA | NA |
| TCGA-DU-A76R | 648 | 1 | 1 | Male | O | 51 | Right | G3 | Mutant | codel | Methylated | NA | WT | NA | NA |
| TCGA-DU-A7T6 | 547 | 1 | 1 | Female | O | 73 | Right | G3 | Mutant | codel | Methylated | NA | WT | NA | NA |
| TCGA-DU-A7T8 | 4229 | 1 | 1 | Male | OA | 35 | Right | G3 | Mutant | non-codel | Methylated | NA | Mutant | NR | NR |
| TCGA-DU-A7TA | 2565 | 0 | 1 | Male | O | 32 | Right | G2 | Mutant | non-codel | Methylated | NA | Mutant | NR | NA |
| TCGA-DU-A7TB | 1567 | 0 | 1 | Male | O | 56 | Right | G2 | WT | non-codel | Unmethylated | NA | WT | NR | NR |
| TCGA-DU-A7TC | 1137 | 0 | 1 | Male | A | 32 | Right | G2 | Mutant | non-codel | Methylated | NA | WT | NA | NA |
| TCGA-DU-A7TD | 228 | 1 | 1 | Male | OA | 52 | Right | G3 | WT | non-codel | Unmethylated | NA | WT | NA | NA |
| TCGA-DU-A7TG | 1351 | 1 | 1 | Male | O | 40 | Right | G2 | Mutant | non-codel | Methylated | NA | WT | NA | NA |
| TCGA-DU-A7TI | 1183 | 1 | 2 | Male | A | 32 | Left | G3 | NA | non-codel | Methylated | NA | NA | NA | NA |
| TCGA-DU-A7TJ | 17 | 0 | 2 | Male | A | 55 | Right | G3 | WT | non-codel | Methylated | NA | WT | NA | NA |
| TCGA-E1-A7YD | 435 | 1 | 1 | Male | A | 57 | Left | G3 | WT | non-codel | Unmethylated | NA | WT | NA | NA |
| TCGA-E1-A7YE | 886 | 1 | 1 | Female | A | 32 | Left | G3 | Mutant | non-codel | Unmethylated | NA | Mutant | NR | NR |
| TCGA-E1-A7YH | 2835 | 1 | 1 | Female | A | 47 | Right | G3 | Mutant | non-codel | Methylated | NA | Mutant | NR | NR |
| TCGA-E1-A7YI | 111 | 1 | 1 | Female | A | 33 | Right | G3 | Mutant | non-codel | Methylated | NA | Mutant | NA | NA |
| TCGA-E1-A7YJ | 592 | 1 | 1 | Male | A | 55 | Right | G3 | WT | non-codel | Unmethylated | NA | WT | NR | NR |
| TCGA-E1-A7YK | 378 | 1 | 1 | Male | A | 52 | Left | G3 | Mutant | non-codel | Methylated | NA | Mutant | NR | NA |
| TCGA-E1-A7YL | 492 | 1 | 1 | Male | A | 46 | Right | G3 | WT | non-codel | Unmethylated | NA | WT | NR | NR |
| TCGA-E1-A7YM | 648 | 1 | 1 | Male | A | 63 | Left | G3 | WT | non-codel | Unmethylated | NA | WT | NR | NR |
| TCGA-E1-A7YN | 727 | 1 | 1 | Female | A | 63 | Right | G3 | WT | non-codel | Methylated | NA | WT | NR | NR |
| TCGA-E1-A7YO | 2282 | 1 | 1 | Male | O | 45 | Right | G3 | Mutant | codel | Methylated | NA | WT | NA | NR |
| TCGA-E1-A7YS | 466 | 1 | 1 | Male | O | 71 | Left | G3 | Mutant | codel | Methylated | NA | WT | NR | NR |
| TCGA-E1-A7YU | 23 | 1 | 1 | Male | OA | 42 | NA | G3 | Mutant | non-codel | Methylated | NA | WT | NA | NA |
| TCGA-E1-A7YV | 987 | 1 | 1 | Female | OA | 26 | Left | G3 | Mutant | non-codel | Methylated | NA | Mutant | NR | NR |
| TCGA-E1-A7YW | 1120 | 1 | 1 | Male | OA | 28 | Left | G2 | Mutant | non-codel | Methylated | NA | Mutant | NR | NR |
| TCGA-E1-A7YY | 4445 | 1 | 1 | Female | O | 27 | Right | G2 | Mutant | non-codel | Methylated | NA | WT | NR | NR |
| TCGA-E1-A7Z2 | 398 | 1 | 1 | Female | O | 58 | Left | G2 | WT | non-codel | Unmethylated | NA | WT | NR | NR |
| TCGA-E1-A7Z3 | 2235 | 1 | 1 | Female | A | 31 | Right | G2 | Mutant | non-codel | Methylated | NA | Mutant | NR | NR |
| TCGA-E1-A7Z4 | 4412 | 1 | 1 | Male | A | 35 | Left | G2 | Mutant | non-codel | Methylated | NA | Mutant | NR | NR |
| TCGA-E1-A7Z6 | 984 | 1 | 1 | Female | A | 41 | Right | G2 | Mutant | non-codel | Methylated | NA | Mutant | NR | NR |
| TCGA-EZ-7264 | 1201 | 0 | 2 | Female | O | 47 | Right | G2 | Mutant | codel | Methylated | Mutant | WT | NR | NR |
| TCGA-F6-A8O3 | 7 | 0 | 1 | Male | O | 34 | NA | G2 | Mutant | codel | Methylated | NA | WT | NA | NA |
| TCGA-F6-A8O4 | 6 | 0 | 1 | Male | A | 44 | Left | G2 | Mutant | non-codel | Methylated | NA | Mutant | NA | NA |
| TCGA-FG-5962 | 1453 | 0 | 1 | Male | O | 54 | Left | G3 | Mutant | codel | Methylated | Mutant | WT | NR | NR |
| TCGA-FG-5963 | 775 | 1 | 1 | Male | A | 23 | Right | G3 | WT | non-codel | Unmethylated | WT | Mutant | NR | NR |
| TCGA-FG-5964 | 1588 | 0 | 2 | Male | O | 62 | Left | G2 | Mutant | codel | Methylated | Mutant | WT | NR | NR |
| TCGA-FG-5965 | 1120 | 1 | 1 | Female | OA | 39 | Right | G2 | Mutant | non-codel | Methylated | WT | Mutant | NR | NR |
| TCGA-FG-6688 | 571 | 0 | 1 | Female | A | 59 | Left | G3 | WT | non-codel | Methylated | Mutant | WT | NR | NR |
| TCGA-FG-6689 | 454 | 0 | 1 | Male | A | 30 | Right | G2 | Mutant | non-codel | Methylated | NA | Mutant | NR | NR |
| TCGA-FG-6690 | 1294 | 0 | 2 | Male | O | 70 | Left | G2 | Mutant | non-codel | Methylated | WT | Mutant | NR | NR |
| TCGA-FG-6691 | 1257 | 0 | 1 | Female | A | 23 | Right | G2 | Mutant | non-codel | Unmethylated | WT | Mutant | R | NR |
| TCGA-FG-6692 | 561 | 1 | 2 | Male | O | 63 | Right | G3 | WT | non-codel | Methylated | Mutant | WT | NR | NR |
| TCGA-FG-7634 | 467 | 0 | 2 | Male | O | 28 | Left | G2 | Mutant | codel | Methylated | Mutant | WT | NR | NR |
| TCGA-FG-7636 | 544 | 0 | 2 | Male | A | 48 | Left | G3 | Mutant | non-codel | Methylated | WT | Mutant | R | R |
| TCGA-FG-7637 | 1219 | 0 | 1 | Male | OA | 49 | Right | G2 | Mutant | non-codel | Methylated | WT | WT | NR | NR |
| TCGA-FG-7638 | 686 | 0 | 2 | Female | O | 31 | Left | G3 | Mutant | codel | Methylated | Mutant | Mutant | NA | NR |
| TCGA-FG-7641 | 627 | 0 | 2 | Male | O | 31 | Left | G2 | Mutant | codel | Methylated | Mutant | WT | R | NR |
| TCGA-FG-7643 | 611 | 0 | 1 | Female | OA | 49 | Right | G2 | WT | non-codel | Methylated | Mutant | WT | NR | NR |
| TCGA-FG-8181 | 862 | 0 | 2 | Male | OA | 23 | Left | G3 | WT | non-codel | Unmethylated | WT | WT | NR | NR |
| TCGA-FG-8182 | 416 | 0 | 2 | Male | O | 35 | Left | G2 | Mutant | non-codel | Methylated | WT | Mutant | NR | NR |
| TCGA-FG-8185 | 433 | 0 | 2 | Male | A | 37 | Right | G3 | Mutant | non-codel | Methylated | WT | Mutant | R | R |
| TCGA-FG-8186 | 487 | 0 | 1 | Female | OA | 42 | Right | G3 | Mutant | codel | Methylated | Mutant | WT | NR | NR |
| TCGA-FG-8187 | 611 | 0 | 2 | Male | OA | 31 | Right | G2 | Mutant | codel | Methylated | Mutant | WT | R | NR |
| TCGA-FG-8188 | 455 | 0 | 1 | Male | OA | 41 | Left | G2 | Mutant | non-codel | Methylated | WT | Mutant | NR | NA |
| TCGA-FG-8189 | 685 | 0 | 1 | Female | O | 33 | Right | G2 | Mutant | non-codel | Methylated | WT | WT | NR | NR |
| TCGA-FG-8191 | 992 | 0 | 2 | Male | O | 30 | Right | G3 | Mutant | non-codel | Unmethylated | WT | Mutant | NR | NR |
| TCGA-FG-A4MT | 1164 | 0 | 1 | Female | O | 27 | Left | G2 | Mutant | non-codel | Methylated | WT | Mutant | R | R |
| TCGA-FG-A4MU | 326 | 0 | 1 | Male | OA | 58 | Right | G3 | WT | non-codel | Methylated | Mutant | WT | NA | NA |
| TCGA-FG-A4MW | 559 | 1 | 1 | Male | OA | 63 | Right | G3 | WT | non-codel | Methylated | Mutant | WT | NR | NR |
| TCGA-FG-A4MX | 569 | 0 | 1 | Male | A | 47 | Right | G2 | Mutant | non-codel | Methylated | WT | Mutant | R | R |
| TCGA-FG-A4MY | 721 | 0 | 2 | Female | OA | 44 | Right | G2 | Mutant | non-codel | Methylated | WT | Mutant | NR | NR |
| TCGA-FG-A60J | 449 | 0 | 2 | Female | OA | 47 | Left | G2 | Mutant | non-codel | Methylated | Mutant | WT | R | NA |
| TCGA-FG-A60K | 542 | 0 | 1 | Female | OA | 34 | Right | G2 | Mutant | codel | Methylated | Mutant | WT | NR | NR |
| TCGA-FG-A60L | 655 | 0 | 1 | Female | A | 34 | Left | G2 | Mutant | non-codel | Methylated | NA | WT | R | R |
| TCGA-FG-A6IZ | 457 | 0 | 2 | Male | O | 60 | Left | G2 | Mutant | codel | Methylated | NA | WT | R | R |
| TCGA-FG-A6J1 | 500 | 0 | 2 | Female | O | 44 | Left | G2 | Mutant | codel | Methylated | NA | WT | NR | NR |
| TCGA-FG-A6J3 | 677 | 0 | 1 | Female | A | 52 | Right | G3 | Mutant | non-codel | Methylated | NA | WT | NR | NR |
| TCGA-FG-A70Y | 868 | 0 | 1 | Female | O | 20 | Left | G2 | Mutant | non-codel | Methylated | NA | Mutant | R | NR |
| TCGA-FG-A70Z | 328 | 0 | 1 | Female | OA | 53 | Left | G3 | WT | non-codel | Methylated | NA | WT | NR | NR |
| TCGA-FG-A710 | 1115 | 0 | 1 | Female | O | 50 | Left | G2 | Mutant | codel | Methylated | NA | WT | NR | NR |
| TCGA-FG-A711 | 1481 | 1 | 1 | Female | O | 33 | Left | G2 | Mutant | non-codel | Unmethylated | NA | Mutant | NR | NR |
| TCGA-FG-A713 | 622 | 0 | 1 | Female | OA | 74 | Right | G2 | Mutant | codel | Methylated | NA | Mutant | R | R |
| TCGA-FG-A87N | 567 | 0 | 1 | Female | A | 37 | Left | G3 | Mutant | non-codel | Methylated | NA | WT | NR | NR |
| TCGA-FG-A87Q | 174 | 0 | 1 | Female | A | 61 | Right | G3 | WT | non-codel | Methylated | NA | WT | R | NA |
| TCGA-FN-7833 | 837 | 0 | 2 | Male | OA | 25 | Midline | G3 | Mutant | non-codel | Methylated | WT | Mutant | R | R |
| TCGA-HT-7467 | 3 | 0 | 1 | Male | O | 54 | Right | G2 | Mutant | codel | Methylated | Mutant | WT | R | R |
| TCGA-HT-7468 | 203 | 0 | 1 | Male | O | 30 | Right | G3 | Mutant | codel | Methylated | Mutant | WT | NR | NR |
| TCGA-HT-7469 | 351 | 1 | 1 | Male | O | 30 | Left | G3 | WT | non-codel | Methylated | WT | Mutant | NR | NR |
| TCGA-HT-7470 | 1220 | 1 | 1 | Male | O | 37 | Right | G3 | Mutant | non-codel | Methylated | WT | Mutant | NR | NR |
| TCGA-HT-7472 | 1 | 0 | 1 | Male | O | 38 | Right | G2 | Mutant | non-codel | Methylated | WT | Mutant | NR | NR |
| TCGA-HT-7473 | 503 | 0 | 1 | Male | OA | 28 | Right | G2 | Mutant | non-codel | Unmethylated | WT | Mutant | R | NA |
| TCGA-HT-7474 | 114 | 0 | 1 | Male | OA | 52 | Right | G2 | Mutant | non-codel | Methylated | WT | Mutant | NR | NR |
| TCGA-HT-7475 | 530 | 0 | 1 | Male | OA | 67 | Right | G3 | Mutant | non-codel | Methylated | WT | Mutant | R | R |
| TCGA-HT-7476 | 199 | 0 | 1 | Male | A | 26 | Left | G2 | Mutant | non-codel | Methylated | WT | Mutant | NR | NA |
| TCGA-HT-7477 | 738 | 0 | 1 | Male | A | 62 | Left | G3 | Mutant | non-codel | Methylated | WT | Mutant | R | R |
| TCGA-HT-7478 | 194 | 0 | 2 | Male | A | 36 | Left | G2 | Mutant | non-codel | Unmethylated | WT | Mutant | R | R |
| TCGA-HT-7479 | 1227 | 0 | 1 | Male | A | 44 | Right | G3 | Mutant | non-codel | Methylated | Mutant | WT | R | R |
| TCGA-HT-7480 | 2287 | 0 | 1 | Male | O | 33 | Right | G2 | Mutant | codel | Methylated | Mutant | WT | NR | NR |
| TCGA-HT-7481 | 2918 | 0 | 1 | Male | O | 39 | Right | G2 | Mutant | codel | Methylated | Mutant | WT | R | R |
| TCGA-HT-7482 | 3253 | 0 | 1 | Female | OA | 18 | Left | G2 | Mutant | non-codel | Methylated | WT | Mutant | R | R |
| TCGA-HT-7483 | 5255 | 0 | 1 | Male | OA | 14 | Right | G2 | Mutant | non-codel | Unmethylated | WT | Mutant | R | R |
| TCGA-HT-7485 | 122 | 0 | 1 | Male | A | 42 | Right | G2 | Mutant | non-codel | Methylated | WT | Mutant | R | R |
| TCGA-HT-7601 | 153 | 0 | 1 | Female | A | 30 | Left | G3 | Mutant | non-codel | Methylated | WT | WT | R | NA |
| TCGA-HT-7602 | 908 | 0 | 1 | Male | O | 21 | Right | G2 | Mutant | non-codel | Methylated | WT | WT | NA | NA |
| TCGA-HT-7603 | 705 | 0 | 1 | Male | O | 29 | NA | G2 | Mutant | non-codel | Methylated | WT | Mutant | R | NA |
| TCGA-HT-7604 | 3725 | 0 | 1 | Male | A | 50 | Left | G2 | Mutant | non-codel | Methylated | WT | Mutant | R | R |
| TCGA-HT-7605 | 139 | 0 | 1 | Male | O | 38 | Right | G2 | Mutant | codel | Methylated | Mutant | WT | NA | NA |
| TCGA-HT-7606 | 526 | 0 | 1 | Female | A | 30 | Left | G2 | Mutant | non-codel | Unmethylated | WT | WT | R | NA |
| TCGA-HT-7607 | 96 | 1 | 1 | Female | A | 61 | Left | G2 | Mutant | codel | Methylated | Mutant | WT | NR | NR |
| TCGA-HT-7608 | 671 | 0 | 1 | Male | OA | 61 | Right | G2 | Mutant | codel | Methylated | Mutant | WT | NR | NR |
| TCGA-HT-7609 | 1399 | 0 | 1 | Male | OA | 34 | Right | G3 | Mutant | non-codel | Methylated | WT | WT | NR | NR |
| TCGA-HT-7610 | 1706 | 0 | 1 | Female | OA | 25 | Left | G2 | Mutant | non-codel | Methylated | WT | Mutant | NR | NR |
| TCGA-HT-7611 | 1752 | 0 | 1 | Male | OA | 36 | Right | G2 | Mutant | non-codel | Methylated | WT | Mutant | NA | NA |
| TCGA-HT-7616 | 7 | 1 | 1 | Male | O | 75 | Right | G3 | Mutant | codel | Methylated | Mutant | WT | NR | NR |
| TCGA-HT-7620 | 434 | 0 | 1 | Male | O | 40 | Left | G3 | Mutant | codel | Methylated | Mutant | WT | R | NR |
| TCGA-HT-7676 | 5 | 0 | 1 | Male | O | 26 | Left | G2 | Mutant | non-codel | Unmethylated | WT | Mutant | NA | NA |
| TCGA-HT-7677 | 494 | 0 | 1 | Male | O | 53 | Left | G3 | Mutant | codel | Methylated | Mutant | WT | NR | NR |
| TCGA-HT-7680 | 23 | 0 | 1 | Female | A | 32 | Midline | G2 | WT | non-codel | Unmethylated | WT | WT | NR | NR |
| TCGA-HT-7681 | 1359 | 0 | 1 | Female | OA | 29 | Right | G2 | Mutant | codel | Methylated | Mutant | WT | R | R |
| TCGA-HT-7684 | 184 | 0 | 1 | Male | OA | 58 | Left | G3 | Mutant | non-codel | Methylated | Mutant | WT | R | R |
| TCGA-HT-7686 | 1300 | 0 | 1 | Female | A | 29 | Left | G3 | Mutant | non-codel | Methylated | WT | Mutant | R | R |
| TCGA-HT-7687 | 3 | 0 | 1 | Male | O | 74 | Right | G3 | Mutant | codel | Methylated | Mutant | WT | NR | NA |
| TCGA-HT-7688 | 964 | 0 | 1 | Male | O | 59 | Left | G3 | Mutant | non-codel | Methylated | WT | Mutant | R | R |
| TCGA-HT-7689 | 455 | 0 | 1 | Female | O | 58 | Left | G2 | Mutant | non-codel | Methylated | WT | Mutant | R | NA |
| TCGA-HT-7690 | 3 | 0 | 1 | Male | OA | 29 | Right | G3 | Mutant | non-codel | Methylated | WT | Mutant | NA | NA |
| TCGA-HT-7691 | 3 | 0 | 1 | Female | A | 31 | Right | G2 | WT | non-codel | Unmethylated | WT | WT | NA | NA |
| TCGA-HT-7692 | 90 | 0 | 1 | Male | OA | 43 | Left | G2 | Mutant | codel | Methylated | Mutant | WT | R | NA |
| TCGA-HT-7693 | 533 | 0 | 1 | Female | O | 51 | Right | G2 | Mutant | non-codel | Methylated | WT | Mutant | R | NA |
| TCGA-HT-7694 | 210 | 0 | 1 | Male | O | 60 | Right | G3 | Mutant | codel | Methylated | Mutant | WT | R | NA |
| TCGA-HT-7695 | 442 | 0 | 1 | Female | O | 29 | Left | G2 | Mutant | codel | Methylated | Mutant | WT | R | NA |
| TCGA-HT-7854 | 1201 | 0 | 1 | Male | A | 62 | Left | G2 | WT | non-codel | Unmethylated | Mutant | WT | NR | NR |
| TCGA-HT-7855 | 585 | 0 | 1 | Male | A | 39 | Left | G3 | Mutant | non-codel | Methylated | WT | Mutant | R | R |
| TCGA-HT-7856 | 1189 | 0 | 1 | Male | O | 35 | Left | G3 | Mutant | codel | Methylated | Mutant | WT | R | R |
| TCGA-HT-7857 | 7 | 0 | 1 | Female | A | 24 | Left | G3 | WT | non-codel | Unmethylated | WT | Mutant | NA | NA |
| TCGA-HT-7858 | 1540 | 0 | 1 | Male | A | 28 | Right | G2 | Mutant | non-codel | Methylated | WT | Mutant | R | R |
| TCGA-HT-7860 | 15 | 0 | 1 | Female | A | 60 | Left | G3 | WT | non-codel | Methylated | Mutant | WT | NA | NA |
| TCGA-HT-7873 | 718 | 0 | 1 | Male | OA | 29 | Right | G2 | Mutant | non-codel | Methylated | WT | Mutant | NR | NR |
| TCGA-HT-7874 | 1130 | 0 | 1 | Female | O | 41 | Right | G3 | Mutant | codel | Methylated | Mutant | WT | R | R |
| TCGA-HT-7875 | 10 | 0 | 1 | Male | O | 56 | Left | G2 | Mutant | codel | Methylated | Mutant | WT | NR | NR |
| TCGA-HT-7877 | 4 | 0 | 1 | Female | O | 20 | Left | G2 | Mutant | codel | Methylated | Mutant | WT | NA | NA |
| TCGA-HT-7879 | 112 | 0 | 1 | Male | OA | 31 | Right | G3 | Mutant | non-codel | Methylated | WT | Mutant | R | NA |
| TCGA-HT-7880 | 162 | 0 | 1 | Male | OA | 30 | Right | G2 | Mutant | non-codel | Methylated | WT | Mutant | NA | NA |
| TCGA-HT-7881 | 1079 | 0 | 1 | Male | O | 38 | Left | G2 | Mutant | codel | Methylated | Mutant | WT | R | R |
| TCGA-HT-7882 | 113 | 1 | 1 | Male | O | 66 | Left | G3 | WT | non-codel | Methylated | Mutant | WT | NR | NR |
| TCGA-HT-7884 | 343 | 0 | 2 | Female | A | 44 | Left | G2 | Mutant | non-codel | Methylated | WT | Mutant | NR | NR |
| TCGA-HT-7902 | 956 | 0 | 1 | Female | OA | 30 | Right | G2 | Mutant | non-codel | Methylated | WT | Mutant | NR | NR |
| TCGA-HT-8010 | 50 | 0 | 1 | Female | O | 64 | Right | G2 | Mutant | codel | Methylated | WT | WT | NR | NR |
| TCGA-HT-8011 | 494 | 0 | 1 | Male | A | 55 | Right | G3 | WT | non-codel | Unmethylated | Mutant | WT | NR | NR |
| TCGA-HT-8012 | 286 | 0 | 1 | Female | O | 30 | Right | G2 | Mutant | codel | Methylated | Mutant | WT | NR | NR |
| TCGA-HT-8013 | 1933 | 1 | 1 | Female | OA | 37 | Left | G2 | Mutant | non-codel | Methylated | WT | Mutant | NR | NA |
| TCGA-HT-8015 | 3 | 0 | 1 | Male | A | 21 | Left | G2 | WT | non-codel | Unmethylated | WT | WT | NA | NA |
| TCGA-HT-8018 | 1152 | 1 | 1 | Female | OA | 40 | Left | G2 | Mutant | non-codel | Methylated | WT | WT | R | NR |
| TCGA-HT-8019 | 1004 | 0 | 1 | Female | O | 34 | Right | G3 | WT | non-codel | Unmethylated | WT | WT | R | R |
| TCGA-HT-8104 | 372 | 0 | 1 | Female | A | 51 | Left | G3 | WT | non-codel | Unmethylated | Mutant | WT | NA | NA |
| TCGA-HT-8105 | 190 | 0 | 1 | Male | O | 54 | Right | G3 | Mutant | codel | Methylated | Mutant | WT | NR | NR |
| TCGA-HT-8106 | 3 | 0 | 1 | Male | A | 53 | Midline | G3 | Mutant | non-codel | Methylated | WT | WT | NR | NR |
| TCGA-HT-8107 | 14 | 0 | 1 | Male | O | 62 | Left | G2 | WT | non-codel | Methylated | WT | WT | NR | NR |
| TCGA-HT-8108 | 76 | 0 | 1 | Female | O | 26 | Left | G2 | Mutant | non-codel | Methylated | WT | Mutant | NR | NR |
| TCGA-HT-8109 | 169 | 0 | 1 | Male | O | 64 | Right | G3 | Mutant | codel | Methylated | Mutant | WT | NA | NA |
| TCGA-HT-8110 | 419 | 0 | 1 | Male | A | 57 | Right | G3 | WT | non-codel | Methylated | Mutant | WT | R | R |
| TCGA-HT-8111 | 7 | 0 | 1 | Male | OA | 32 | Left | G3 | Mutant | non-codel | Methylated | WT | WT | NA | NA |
| TCGA-HT-8113 | 900 | 0 | 1 | Female | O | 49 | Left | G2 | Mutant | non-codel | Methylated | Mutant | WT | R | NA |
| TCGA-HT-8114 | 1040 | 0 | 1 | Male | OA | 36 | Left | G3 | Mutant | non-codel | Methylated | WT | Mutant | R | R |
| TCGA-HT-8558 | 418 | 0 | 1 | Female | O | 29 | Left | G2 | WT | non-codel | Unmethylated | WT | WT | R | R |
| TCGA-HT-8563 | 860 | 0 | 1 | Female | A | 30 | Right | G3 | Mutant | non-codel | Unmethylated | WT | Mutant | R | R |
| TCGA-HT-8564 | 478 | 0 | 1 | Male | A | 47 | Left | G3 | WT | non-codel | Unmethylated | WT | WT | NR | NR |
| TCGA-HT-A4DS | 7 | 0 | 1 | Female | A | 55 | Right | G3 | WT | non-codel | Unmethylated | Mutant | WT | NA | NA |
| TCGA-HT-A4DV | 792 | 0 | 1 | Female | O | 51 | Left | G3 | Mutant | codel | Methylated | Mutant | WT | R | NA |
| TCGA-HT-A5R5 | 4 | 0 | 1 | Female | O | 33 | Left | G2 | Mutant | non-codel | Methylated | WT | Mutant | NA | NA |
| TCGA-HT-A5R7 | 615 | 0 | 1 | Female | A | 33 | Right | G3 | Mutant | non-codel | Methylated | WT | Mutant | R | R |
| TCGA-HT-A5R9 | 2 | 0 | 1 | Female | O | 48 | Right | G3 | Mutant | codel | Methylated | Mutant | WT | NA | NA |
| TCGA-HT-A5RA | 832 | 0 | 1 | Female | A | 65 | Right | G3 | WT | non-codel | Unmethylated | Mutant | WT | R | R |
| TCGA-HT-A5RB | 3 | 0 | 1 | Male | A | 24 | Right | G2 | Mutant | non-codel | Methylated | WT | Mutant | NA | NA |
| TCGA-HT-A5RC | 162 | 1 | 1 | Female | A | 70 | Left | G3 | WT | non-codel | Unmethylated | WT | WT | NA | NA |
| TCGA-HT-A614 | 82 | 0 | 1 | Male | OA | 47 | Right | G2 | Mutant | non-codel | Methylated | WT | Mutant | R | R |
| TCGA-HT-A615 | 515 | 0 | 1 | Female | O | 38 | Left | G2 | Mutant | codel | Methylated | Mutant | WT | R | R |
| TCGA-HT-A616 | 55 | 0 | 1 | Female | A | 36 | Left | G2 | Mutant | non-codel | Methylated | WT | Mutant | NA | NA |
| TCGA-HT-A617 | 491 | 0 | 1 | Male | O | 47 | Right | G2 | WT | non-codel | Unmethylated | Mutant | WT | NA | NA |
| TCGA-HT-A618 | 512 | 0 | 1 | Female | A | 37 | NA | G3 | Mutant | non-codel | Methylated | WT | Mutant | R | R |
| TCGA-HT-A619 | 651 | 0 | 1 | Female | O | 51 | Left | G3 | Mutant | codel | Methylated | Mutant | WT | R | R |
| TCGA-HT-A61A | 194 | 0 | 1 | Female | O | 20 | Left | G2 | Mutant | non-codel | Methylated | Mutant | WT | NA | NA |
| TCGA-HT-A61B | 533 | 0 | 1 | Male | A | 22 | Left | G3 | Mutant | non-codel | Methylated | WT | WT | R | R |
| TCGA-HT-A61C | 537 | 1 | 1 | Male | O | 66 | Left | G3 | WT | non-codel | Unmethylated | Mutant | WT | R | NR |
| TCGA-HT-A74H | 74 | 0 | 1 | Male | A | 62 | Left | G3 | WT | non-codel | Unmethylated | NA | WT | NA | NA |
| TCGA-HT-A74J | 320 | 0 | 1 | Male | OA | 33 | Left | G2 | Mutant | non-codel | Unmethylated | NA | Mutant | NA | NA |
| TCGA-HT-A74K | 467 | 0 | 1 | Female | O | 58 | Right | G3 | Mutant | codel | Methylated | NA | WT | NA | NA |
| TCGA-HT-A74L | 336 | 0 | 1 | Female | OA | 22 | Right | G2 | Mutant | codel | Methylated | NA | WT | R | R |
| TCGA-HT-A74O | 3 | 0 | 1 | Male | A | 34 | Left | G3 | Mutant | non-codel | Methylated | NA | Mutant | NA | NA |
| TCGA-HW-7486 | 1721 | 0 | 1 | Male | O | 37 | Right | G2 | Mutant | codel | Methylated | Mutant | WT | NR | NR |
| TCGA-HW-7487 | 1382 | 0 | 1 | Male | O | 39 | Left | G2 | Mutant | codel | Methylated | Mutant | WT | NR | NR |
| TCGA-HW-7489 | 1262 | 1 | 1 | Male | OA | 38 | Right | G2 | Mutant | non-codel | Methylated | WT | Mutant | NR | NR |
| TCGA-HW-7490 | 1387 | 0 | 1 | Male | A | 41 | Left | G2 | Mutant | non-codel | Methylated | WT | Mutant | NR | NR |
| TCGA-HW-7491 | 2078 | 0 | 1 | Male | O | 35 | Right | G2 | Mutant | codel | Unmethylated | Mutant | WT | NR | NR |
| TCGA-HW-7493 | 2107 | 0 | 1 | Female | A | 40 | Right | G2 | NA | non-codel | Methylated | NA | NA | NR | NR |
| TCGA-HW-7495 | 1078 | 0 | 1 | Female | O | 45 | Right | G2 | Mutant | codel | Methylated | Mutant | WT | NR | NR |
| TCGA-HW-8319 | 1209 | 1 | 2 | Female | A | 34 | Left | G3 | Mutant | non-codel | Unmethylated | WT | Mutant | NR | NR |
| TCGA-HW-8320 | 1217 | 0 | 1 | Male | A | 36 | Right | G3 | Mutant | non-codel | Methylated | WT | WT | NR | NR |
| TCGA-HW-8321 | 1294 | 0 | 2 | Male | A | 31 | Left | G3 | Mutant | non-codel | Methylated | WT | WT | NR | NR |
| TCGA-HW-8322 | 758 | 0 | 1 | Male | O | 39 | Left | G2 | Mutant | codel | Methylated | Mutant | WT | NR | NR |
| TCGA-HW-A5KJ | 962 | 1 | 2 | Male | O | 68 | Right | G3 | Mutant | codel | Methylated | Mutant | WT | NR | NR |
| TCGA-HW-A5KK | 388 | 1 | 2 | Male | A | 64 | Right | G3 | WT | non-codel | Methylated | Mutant | WT | NR | NA |
| TCGA-HW-A5KL | 878 | 0 | 2 | Female | A | 42 | Right | G2 | Mutant | non-codel | Methylated | WT | Mutant | NR | NR |
| TCGA-HW-A5KM | 630 | 0 | 2 | Male | A | 35 | Left | G2 | Mutant | non-codel | Methylated | WT | WT | NR | NR |
| TCGA-IK-7675 | 578 | 1 | 2 | Male | O | 43 | Right | G2 | Mutant | non-codel | Methylated | WT | Mutant | NR | NR |
| TCGA-IK-8125 | 1301 | 0 | 1 | Male | OA | 62 | Left | G3 | Mutant | codel | Methylated | Mutant | WT | R | R |
| TCGA-KT-A74X | 438 | 0 | 1 | Male | OA | 26 | Left | G3 | Mutant | codel | Methylated | NA | WT | NR | NR |
| TCGA-KT-A7W1 | 438 | 0 | 1 | Female | A | 45 | Right | G3 | WT | non-codel | Methylated | NA | WT | NR | NR |
| TCGA-P5-A72U | 7 | 0 | 1 | Female | O | 71 | Right | G3 | WT | non-codel | Methylated | NA | WT | R | NA |
| TCGA-P5-A72W | 317 | 0 | 1 | Male | A | 35 | Left | G3 | Mutant | non-codel | Unmethylated | NA | Mutant | NA | NA |
| TCGA-P5-A72X | 403 | 0 | 1 | Male | A | 21 | Left | G3 | Mutant | non-codel | Methylated | NA | WT | NA | NA |
| TCGA-P5-A72Z | 354 | 0 | 1 | Female | O | 64 | Left | G3 | Mutant | codel | Methylated | NA | WT | NA | NA |
| TCGA-P5-A730 | 333 | 0 | 1 | Male | OA | 22 | Right | G3 | Mutant | codel | Methylated | NA | WT | R | NA |
| TCGA-P5-A731 | 274 | 0 | 1 | Female | OA | 59 | Right | G2 | Mutant | non-codel | Methylated | NA | WT | NA | NA |
| TCGA-P5-A733 | 414 | 0 | 1 | Female | A | 52 | Left | G2 | Mutant | non-codel | Methylated | NA | Mutant | NA | NA |
| TCGA-P5-A735 | 292 | 0 | 1 | Female | A | 38 | Left | G2 | Mutant | non-codel | Methylated | NA | Mutant | R | R |
| TCGA-P5-A736 | 230 | 0 | 1 | Female | A | 44 | Right | G3 | Mutant | non-codel | Methylated | NA | Mutant | R | R |
| TCGA-P5-A737 | 372 | 0 | 1 | Male | OA | 47 | Right | G2 | Mutant | codel | Methylated | NA | WT | NA | NA |
| TCGA-P5-A77W | 584 | 0 | 1 | Female | OA | 37 | Left | G3 | Mutant | codel | Methylated | NA | WT | NA | NA |
| TCGA-P5-A77X | 105 | 0 | 1 | Female | OA | 55 | Right | G2 | Mutant | codel | Methylated | NA | WT | R | R |
| TCGA-P5-A780 | 72 | 0 | 1 | Female | A | 44 | Right | G3 | Mutant | non-codel | Methylated | NA | WT | R | R |
| TCGA-P5-A781 | 134 | 0 | 1 | Female | A | 34 | Right | G3 | Mutant | codel | Methylated | NA | WT | R | R |
| TCGA-QH-A65R | 461 | 0 | 1 | Female | O | 38 | Left | G3 | Mutant | codel | Methylated | NA | WT | R | NR |
| TCGA-QH-A65S | 77 | 0 | 1 | Female | OA | 32 | Left | G2 | Mutant | non-codel | Methylated | WT | Mutant | R | R |
| TCGA-QH-A65V | 384 | 0 | 1 | Female | O | 43 | Right | G2 | Mutant | codel | Methylated | Mutant | WT | NR | NA |
| TCGA-QH-A65X | 482 | 0 | 1 | Female | OA | 28 | Right | G3 | Mutant | codel | Methylated | NA | WT | NR | NR |
| TCGA-QH-A65Z | 427 | 0 | 1 | Male | O | 54 | Right | G2 | Mutant | codel | Methylated | Mutant | WT | NR | NA |
| TCGA-QH-A6CS | 588 | 0 | 1 | Male | A | 41 | Right | G3 | WT | non-codel | Unmethylated | NA | WT | NR | NA |
| TCGA-QH-A6CU | 451 | 0 | 1 | Female | O | 52 | Left | G3 | Mutant | codel | Methylated | NA | WT | NR | NR |
| TCGA-QH-A6CV | 442 | 0 | 1 | Male | OA | 51 | Right | G3 | WT | non-codel | Unmethylated | NA | WT | NR | NR |
| TCGA-QH-A6CW | 414 | 0 | 1 | Male | OA | 43 | Right | G3 | Mutant | non-codel | Methylated | NA | Mutant | R | R |
| TCGA-QH-A6CX | 372 | 1 | 1 | Male | A | 66 | Left | G2 | WT | non-codel | Unmethylated | NA | WT | NR | NA |
| TCGA-QH-A6CY | 408 | 0 | 1 | Male | OA | 38 | Right | G3 | Mutant | codel | Methylated | NA | WT | NR | NR |
| TCGA-QH-A6CZ | 279 | 0 | 1 | Male | OA | 38 | Right | G2 | Mutant | codel | Methylated | NA | WT | NR | NR |
| TCGA-QH-A6X3 | 313 | 0 | 2 | Male | OA | 27 | Right | G2 | Mutant | non-codel | Methylated | NA | Mutant | R | R |
| TCGA-QH-A6X4 | 442 | 0 | 1 | Male | OA | 47 | Left | G3 | Mutant | codel | Methylated | NA | WT | R | R |
| TCGA-QH-A6X5 | 497 | 0 | 1 | Female | OA | 58 | Left | G2 | Mutant | codel | Methylated | NA | WT | NA | NA |
| TCGA-QH-A6X8 | 509 | 0 | 1 | Female | O | 56 | Left | G3 | Mutant | codel | Methylated | NA | WT | NR | NR |
| TCGA-QH-A6X9 | 58 | 0 | 2 | Female | O | 73 | Right | G2 | Mutant | non-codel | Methylated | NA | Mutant | NA | NA |
| TCGA-QH-A6XA | 502 | 0 | 1 | Female | OA | 23 | Left | G2 | Mutant | non-codel | Methylated | NA | Mutant | R | R |
| TCGA-QH-A6XC | 508 | 0 | 1 | Male | A | 48 | Right | G3 | WT | non-codel | Methylated | NA | WT | NA | NA |
| TCGA-QH-A86X | 337 | 0 | 1 | Male | O | 33 | Left | G2 | Mutant | codel | Methylated | NA | WT | R | R |
| TCGA-QH-A870 | 395 | 0 | 1 | Female | OA | 38 | Right | G3 | Mutant | non-codel | Methylated | NA | Mutant | R | NR |
| TCGA-R8-A6MK | 2702 | 0 | 1 | Male | O | 40 | Right | G2 | Mutant | codel | Methylated | NA | WT | NA | NA |
| TCGA-R8-A6ML | 2860 | 0 | 1 | Male | O | 52 | Left | G3 | Mutant | codel | Methylated | NA | WT | NA | NA |
| TCGA-R8-A6MO | 993 | 0 | 1 | Female | O | 53 | Right | G2 | Mutant | codel | Methylated | NA | WT | NA | NA |
| TCGA-R8-A73M | 1806 | 0 | 1 | Female | O | 48 | Left | G2 | Mutant | codel | Methylated | NA | WT | NA | NA |
| TCGA-RY-A83X | 939 | 0 | 1 | Female | O | 46 | Right | G2 | Mutant | codel | Methylated | NA | WT | NA | NR |
| TCGA-RY-A83Y | 166 | 0 | 1 | Male | O | 45 | Left | G2 | Mutant | codel | Methylated | NA | WT | NA | NA |
| TCGA-RY-A83Z | 301 | 0 | 1 | Female | A | 54 | Left | G3 | Mutant | non-codel | Methylated | NA | Mutant | NA | NA |
| TCGA-RY-A840 | 854 | 0 | 1 | Male | O | 47 | Left | G3 | Mutant | codel | Methylated | NA | WT | NR | NR |
| TCGA-RY-A843 | 63 | 0 | 1 | Male | A | 30 | Left | G3 | Mutant | non-codel | Methylated | NA | WT | NA | NR |
| TCGA-RY-A845 | 596 | 0 | 1 | Female | OA | 40 | Left | G2 | Mutant | non-codel | Methylated | NA | WT | NA | NA |
| TCGA-RY-A847 | 933 | 0 | 1 | Male | O | 45 | Right | G2 | Mutant | codel | Methylated | NA | WT | NA | NR |
| TCGA-S9-A6TS | 1891 | 1 | 2 | Female | A | 48 | Right | G3 | Mutant | non-codel | Methylated | NA | Mutant | NR | NR |
| TCGA-S9-A6TU | 2650 | 0 | 2 | Male | A | 38 | Left | G2 | Mutant | non-codel | Methylated | NA | WT | R | NR |
| TCGA-S9-A6TV | 571 | 0 | 2 | Male | OA | 50 | Right | G3 | Mutant | non-codel | Methylated | NA | Mutant | R | NA |
| TCGA-S9-A6TW | 1250 | 0 | 1 | Male | O | 40 | Left | G3 | Mutant | codel | Methylated | NA | WT | R | R |
| TCGA-S9-A6TX | 1245 | 0 | 1 | Male | O | 46 | Left | G3 | Mutant | codel | Methylated | NA | WT | R | R |
| TCGA-S9-A6TY | 1076 | 0 | 1 | Male | O | 50 | Left | G2 | Mutant | codel | Methylated | NA | WT | R | NR |
| TCGA-S9-A6TZ | 1120 | 0 | 1 | Female | A | 39 | Left | G2 | Mutant | non-codel | Methylated | NA | WT | R | NR |
| TCGA-S9-A6U0 | 742 | 1 | 1 | Male | A | 46 | Right | G3 | WT | non-codel | Methylated | NA | WT | R | NR |
| TCGA-S9-A6U1 | 785 | 0 | 1 | Female | A | 22 | Right | G3 | Mutant | non-codel | Methylated | NA | Mutant | R | R |
| TCGA-S9-A6U2 | 908 | 0 | 1 | Female | O | 48 | Right | G2 | Mutant | codel | Methylated | NA | WT | R | NR |
| TCGA-S9-A6U5 | 992 | 0 | 1 | Male | A | 33 | Right | G2 | Mutant | codel | Methylated | NA | WT | R | NA |
| TCGA-S9-A6U6 | 1069 | 0 | 2 | Male | A | 28 | Left | G3 | Mutant | non-codel | Methylated | NA | Mutant | R | R |
| TCGA-S9-A6U8 | 2988 | 1 | 1 | Male | A | 24 | Left | G2 | Mutant | non-codel | Methylated | NA | Mutant | R | NR |
| TCGA-S9-A6U9 | 2918 | 0 | 1 | Male | A | 36 | Right | G3 | Mutant | non-codel | Methylated | NA | Mutant | R | NR |
| TCGA-S9-A6UA | 178 | 1 | 2 | Male | A | 66 | Right | G3 | WT | non-codel | Methylated | NA | Mutant | NR | NR |
| TCGA-S9-A6UB | 3761 | 0 | 1 | Male | O | 52 | Right | G2 | Mutant | codel | Methylated | NA | WT | R | NR |
| TCGA-S9-A6WD | 2289 | 0 | 1 | Male | O | 58 | Left | G3 | Mutant | codel | Methylated | NA | WT | R | NR |
| TCGA-S9-A6WE | 4113 | 0 | 2 | Male | O | 34 | Right | G2 | Mutant | codel | Methylated | NA | WT | R | NA |
| TCGA-S9-A6WG | 2602 | 0 | 2 | Male | A | 31 | Right | G3 | Mutant | non-codel | Methylated | NA | Mutant | NR | NR |
| TCGA-S9-A6WH | 1173 | 0 | 1 | Female | OA | 73 | Right | G2 | Mutant | codel | Methylated | NA | WT | R | NR |
| TCGA-S9-A6WI | 2585 | 0 | 1 | Female | OA | 56 | Right | G2 | Mutant | non-codel | Methylated | NA | WT | R | NR |
| TCGA-S9-A6WL | 946 | 0 | 1 | Male | A | 52 | Right | G3 | Mutant | codel | Methylated | NA | WT | NR | NA |
| TCGA-S9-A6WM | 604 | 0 | 2 | Female | A | 59 | Left | G3 | WT | non-codel | Unmethylated | NA | WT | R | NR |
| TCGA-S9-A6WN | 805 | 0 | 2 | Female | A | 38 | Left | G3 | Mutant | codel | Methylated | NA | WT | NR | NR |
| TCGA-S9-A6WO | 566 | 0 | 2 | Male | A | 29 | Left | G2 | Mutant | non-codel | Methylated | NA | Mutant | NR | NR |
| TCGA-S9-A6WP | 567 | 0 | 1 | Male | OA | 42 | Right | G3 | Mutant | codel | Methylated | NA | WT | R | NR |
| TCGA-S9-A6WQ | 428 | 0 | 1 | Female | OA | 57 | Left | G2 | Mutant | non-codel | Methylated | NA | Mutant | R | NR |
| TCGA-S9-A7IQ | 1099 | 0 | 1 | Female | OA | 45 | Left | G2 | Mutant | codel | Methylated | NA | WT | R | NR |
| TCGA-S9-A7IS | 241 | 1 | 1 | Female | A | 33 | Right | G3 | Mutant | non-codel | Methylated | NA | Mutant | NR | NR |
| TCGA-S9-A7IX | 819 | 1 | 1 | Male | A | 57 | Right | G3 | WT | non-codel | Unmethylated | NA | WT | NR | NR |
| TCGA-S9-A7IY | 715 | 0 | 2 | Male | OA | 39 | Right | G3 | Mutant | codel | Methylated | NA | WT | R | R |
| TCGA-S9-A7IZ | 608 | 0 | 1 | Female | A | 48 | Left | G3 | Mutant | non-codel | Methylated | NA | Mutant | R | NR |
| TCGA-S9-A7J0 | 609 | 0 | 1 | Female | O | 30 | Right | G3 | Mutant | non-codel | Methylated | NA | WT | R | NR |
| TCGA-S9-A7J1 | 249 | 0 | 1 | Male | O | 43 | Right | G2 | Mutant | codel | Methylated | NA | WT | R | NA |
| TCGA-S9-A7J2 | 62 | 0 | 1 | Male | O | 25 | Left | G3 | Mutant | codel | Methylated | NA | WT | R | NA |
| TCGA-S9-A7J3 | 629 | 0 | 2 | Female | O | 52 | Left | G3 | Mutant | codel | Methylated | NA | WT | R | NR |
| TCGA-S9-A7QW | 458 | 0 | 2 | Female | A | 54 | Left | G3 | Mutant | non-codel | Methylated | NA | Mutant | R | NR |
| TCGA-S9-A7QX | 564 | 0 | 1 | Female | A | 36 | Right | G3 | Mutant | non-codel | Methylated | NA | Mutant | R | NR |
| TCGA-S9-A7QY | 863 | 0 | 1 | Female | OA | 35 | Left | G2 | Mutant | codel | Methylated | NA | WT | R | NR |
| TCGA-S9-A7QZ | 826 | 0 | 1 | Male | O | 41 | Left | G2 | Mutant | codel | Methylated | NA | WT | R | R |
| TCGA-S9-A7R1 | 5166 | 1 | 1 | Male | O | 35 | Midline | G2 | Mutant | codel | Methylated | NA | WT | R | NA |
| TCGA-S9-A7R2 | 316 | 1 | 2 | Male | A | 69 | Left | G3 | WT | non-codel | Unmethylated | NA | WT | NA | NA |
| TCGA-S9-A7R3 | 3013 | 0 | 1 | Female | A | 28 | Left | G2 | Mutant | non-codel | Methylated | NA | WT | R | NR |
| TCGA-S9-A7R4 | 914 | 0 | 1 | Male | A | 46 | Left | G3 | Mutant | non-codel | Methylated | NA | WT | R | NA |
| TCGA-S9-A7R7 | 3000 | 0 | 2 | Male | A | 27 | Left | G2 | Mutant | non-codel | Methylated | NA | Mutant | NR | NR |
| TCGA-S9-A7R8 | 961 | 1 | 1 | Female | A | 44 | Right | G3 | Mutant | non-codel | Methylated | NA | Mutant | NA | NA |
| TCGA-S9-A89V | 569 | 0 | 1 | Male | A | 70 | Right | G3 | WT | non-codel | Methylated | NA | Mutant | NR | NA |
| TCGA-S9-A89Z | 623 | 0 | 1 | Male | A | 40 | Right | G3 | Mutant | non-codel | Methylated | NA | WT | R | NR |
| TCGA-TM-A7C3 | 1666 | 1 | 2 | Female | A | 43 | Left | G3 | WT | non-codel | Methylated | NA | WT | R | NR |
| TCGA-TM-A7C4 | 1470 | 0 | 2 | Female | A | 39 | Right | G2 | Mutant | non-codel | Methylated | NA | Mutant | R | R |
| TCGA-TM-A7C5 | 1500 | 0 | 1 | Male | OA | 30 | Left | G2 | Mutant | codel | Methylated | NA | WT | R | R |
| TCGA-TM-A7CA | 1058 | 0 | 1 | Male | A | 44 | Left | G2 | Mutant | non-codel | Methylated | NA | Mutant | R | R |
| TCGA-TM-A7CF | 1989 | 0 | 1 | Female | A | 41 | Right | G2 | Mutant | non-codel | Methylated | WT | WT | R | NR |
| TCGA-TM-A84B | 758 | 1 | 1 | Male | A | 40 | Left | G3 | WT | non-codel | Unmethylated | NA | WT | NR | NR |
| TCGA-TM-A84C | 492 | 1 | 1 | Male | A | 32 | Midline | G2 | WT | non-codel | Unmethylated | NA | WT | NR | NR |
| TCGA-TM-A84F | 1796 | 0 | 1 | Male | A | 48 | Right | G3 | Mutant | non-codel | Methylated | NA | WT | R | R |
| TCGA-TM-A84G | 1236 | 0 | 1 | Female | O | 54 | Right | G3 | Mutant | codel | Methylated | NA | WT | R | R |
| TCGA-TM-A84H | 926 | 0 | 1 | Female | OA | 44 | Right | G3 | Mutant | non-codel | Methylated | NA | Mutant | R | R |
| TCGA-TM-A84I | 854 | 0 | 1 | Male | A | 30 | Left | G3 | Mutant | non-codel | Methylated | NA | Mutant | R | R |
| TCGA-TM-A84J | 735 | 0 | 1 | Male | O | 63 | Left | G3 | WT | non-codel | Unmethylated | NA | WT | NR | NR |
| TCGA-TM-A84L | 1242 | 1 | 1 | Male | OA | 31 | Left | G2 | Mutant | non-codel | Methylated | NA | Mutant | R | NR |
| TCGA-TM-A84M | 754 | 0 | 1 | Male | O | 40 | Right | G3 | Mutant | codel | Methylated | NA | WT | R | R |
| TCGA-TM-A84O | 1011 | 1 | 1 | Female | O | 61 | Right | G3 | Mutant | codel | Methylated | NA | WT | R | NR |
| TCGA-TM-A84Q | 772 | 0 | 1 | Male | A | 31 | Left | G2 | Mutant | non-codel | Methylated | NA | Mutant | R | R |
| TCGA-TM-A84R | 566 | 0 | 1 | Male | O | 46 | Left | G2 | Mutant | codel | Methylated | NA | WT | R | R |
| TCGA-TM-A84S | 454 | 0 | 1 | Male | O | 36 | Left | G3 | Mutant | codel | Methylated | NA | WT | R | R |
| TCGA-TM-A84T | 724 | 0 | 1 | Male | OA | 19 | Left | G2 | Mutant | non-codel | Methylated | NA | Mutant | NR | NR |
| TCGA-TQ-A7RF | 650 | 0 | 1 | Female | O | 27 | Right | G3 | Mutant | non-codel | Unmethylated | NA | WT | R | NA |
| TCGA-TQ-A7RG | 599 | 0 | 1 | Male | OA | 36 | Left | G2 | Mutant | codel | Methylated | NA | WT | R | NA |
| TCGA-TQ-A7RH | 486 | 0 | 1 | Male | OA | 39 | Right | G2 | Mutant | non-codel | Methylated | NA | Mutant | R | NA |
| TCGA-TQ-A7RI | 62 | 0 | 1 | Female | O | 37 | Left | G2 | Mutant | codel | Methylated | NA | WT | NA | NA |
| TCGA-TQ-A7RJ | 1229 | 0 | 1 | Female | OA | 25 | Left | G2 | Mutant | non-codel | Methylated | NA | Mutant | NR | NA |
| TCGA-TQ-A7RK | 1341 | 0 | 1 | Male | OA | 29 | Left | G2 | Mutant | non-codel | Methylated | WT | Mutant | R | R |
| TCGA-TQ-A7RM | 1116 | 0 | 1 | Female | OA | 41 | Left | G3 | Mutant | non-codel | Methylated | NA | WT | R | R |
| TCGA-TQ-A7RN | 1026 | 0 | 1 | Male | O | 32 | Left | G2 | Mutant | codel | Methylated | NA | WT | R | R |
| TCGA-TQ-A7RO | 949 | 0 | 1 | Male | OA | 29 | Left | G2 | Mutant | codel | Methylated | NA | WT | R | R |
| TCGA-TQ-A7RP | 914 | 0 | 1 | Male | OA | 66 | Left | G2 | WT | non-codel | Methylated | NA | WT | R | R |
| TCGA-TQ-A7RQ | 795 | 0 | 1 | Female | O | 38 | Left | G2 | Mutant | codel | Methylated | NA | WT | R | R |
| TCGA-TQ-A7RR | 787 | 0 | 1 | Male | OA | 38 | Right | G2 | Mutant | non-codel | Unmethylated | NA | Mutant | R | R |
| TCGA-TQ-A7RU | 1032 | 0 | 1 | Male | O | 51 | Left | G2 | Mutant | codel | Methylated | NA | WT | R | R |
| TCGA-TQ-A7RV | 1868 | 0 | 1 | Male | A | 27 | Left | G2 | Mutant | non-codel | Methylated | WT | Mutant | R | NR |
| TCGA-TQ-A7RW | 821 | 1 | 1 | Male | O | 32 | Left | G2 | Mutant | non-codel | Methylated | NA | WT | NR | NR |
| TCGA-TQ-A8XE | 954 | 1 | 1 | Female | O | 42 | Left | G2 | Mutant | non-codel | Methylated | WT | WT | R | NR |
| TCGA-VM-A8C8 | 1397 | 0 | 1 | Female | O | 50 | Right | G2 | Mutant | non-codel | Unmethylated | NA | Mutant | NA | NR |
| TCGA-VM-A8C9 | 1314 | 0 | 1 | Female | A | 37 | Left | G2 | WT | non-codel | Unmethylated | NA | WT | R | R |
| TCGA-VM-A8CA | 411 | 0 | 1 | Male | O | 54 | Left | G2 | Mutant | non-codel | Methylated | NA | WT | NA | NA |
| TCGA-VM-A8CB | 3 | 0 | 1 | Male | O | 33 | Left | G3 | Mutant | codel | Methylated | NA | WT | NA | NA |
| TCGA-VM-A8CD | 240 | 1 | 1 | Male | A | 58 | Right | G3 | WT | non-codel | Unmethylated | NA | WT | NA | NA |
| TCGA-VM-A8CE | 1191 | 0 | 1 | Male | O | 25 | Left | G2 | Mutant | codel | Methylated | NA | WT | NA | NA |
| TCGA-VM-A8CF | 609 | 0 | 1 | Female | A | 44 | Left | G3 | Mutant | non-codel | Methylated | NA | Mutant | NA | NR |
| TCGA-VM-A8CH | 714 | 0 | 1 | Female | A | 24 | Right | G2 | Mutant | non-codel | Unmethylated | NA | Mutant | R | NA |
| TCGA-VV-A829 | 1127 | 0 | 1 | Male | OA | 44 | Left | G3 | Mutant | codel | Methylated | NA | WT | NR | R |
| TCGA-VV-A86M | 487 | 0 | 1 | Female | A | 36 | Left | G3 | Mutant | non-codel | Methylated | NA | WT | NA | R |
| TCGA-W9-A837 | 1553 | 0 | 1 | Male | O | 47 | Right | G2 | Mutant | codel | Methylated | NA | WT | NR | NR |
| TCGA-WH-A86K | 405 | 0 | 1 | Male | A | 65 | Right | G2 | Mutant | non-codel | Methylated | NA | WT | NR | NR |
| TCGA-WY-A858 | 1337 | 0 | 1 | Female | A | 32 | Left | G3 | Mutant | non-codel | Methylated | NA | Mutant | NA | NA |
| TCGA-WY-A859 | 1213 | 0 | 1 | Female | A | 34 | Left | G2 | Mutant | non-codel | Methylated | NA | WT | NA | NA |
| TCGA-WY-A85A | 1320 | 0 | 1 | Male | A | 20 | Right | G2 | Mutant | non-codel | Methylated | NA | Mutant | NA | NA |
| TCGA-WY-A85B | 1393 | 0 | 1 | Male | A | 24 | Right | G2 | Mutant | non-codel | Methylated | NA | Mutant | R | NA |
| TCGA-WY-A85C | 1426 | 0 | 1 | Male | A | 36 | Right | G2 | Mutant | non-codel | Methylated | NA | WT | NR | NR |
| TCGA-WY-A85D | 1147 | 0 | 1 | Male | OA | 60 | Right | G2 | Mutant | non-codel | Unmethylated | NA | Mutant | NA | NA |
| TCGA-WY-A85E | 633 | 0 | 1 | Female | OA | 48 | Left | G2 | Mutant | non-codel | Methylated | NA | Mutant | R | NR |
| **Abbreviations:** *: "A" means "Astrocytoma"; "O" means "Oligodendroglioma"; "OA" means "Oligoastrocytoma" | | | | | | | | | | | | | | | |

| **Supplementary Table 41.** Metadata of patients in ZN-LGG cohort acquired from Liu et al.^45^ | | | | | | | |
| --- | --- | --- | --- | --- | --- | --- | --- |
| **Patient ID** | **Subtype** | **Histological type*** | **Age of Diagnosis** | **Overall Survival Month** | **Overall Survival Status** | **Gender** | **Grade** |
| ZN-005 | 2 | O | 59 | 31.97 | 0 | Female | 2 |
| ZN-006 | 2 | A | 59 | 27.87 | 1 | Female | 2 |
| ZN-008 | 1 | A | 40 | 33.83 | 0 | Male | 2 |
| ZN-009 | 1 | O | 66 | 33.67 | 0 | Female | 2 |
| ZN-010 | 1 | A | 53 | 33.33 | 0 | Male | 2 |
| ZN-016 | 1 | A | 54 | 31.77 | 0 | Male | 2 |
| ZN-017 | 1 | A | 37 | 29.87 | 0 | Male | 2 |
| ZN-018 | 1 | A | 41 | 29.13 | 0 | Male | 2 |
| ZN-023 | 1 | A | 54 | 24.77 | 0 | Female | 2 |
| ZN-025 | 2 | A | 35 | 22.20 | 0 | Female | 2 |
| ZN-027 | 2 | O | 63 | 21.07 | 0 | Female | 2 |
| ZN-028 | 2 | O | 30 | 20.73 | 0 | Male | 2 |
| ZN-030 | 2 | A | 6 | 17.37 | 0 | Male | 2 |
| ZN-032 | 2 | O | 39 | 17.37 | 0 | Male | 2 |
| ZN-033 | 1 | O | 23 | 15.13 | 0 | Male | 2 |
| ZN-036 | 1 | O | 48 | 14.33 | 0 | Male | 2 |
| ZN-037 | 1 | O | 35 | 13.83 | 0 | Female | 2 |
| ZN-039 | 1 | A | 31 | 13.63 | 0 | Male | 2 |
| ZN-040 | 1 | A | 39 | 13.60 | 0 | Male | 2 |
| ZN-043 | 2 | A | 47 | 12.47 | 0 | Female | 2 |
| ZN-045 | 2 | O | 24 | 11.50 | 0 | Male | 2 |
| ZN-047 | 2 | A | 49 | 11.20 | 0 | Male | 2 |
| ZN-048 | 1 | O | 50 | 11.17 | 0 | Female | 2 |
| ZN-050 | 2 | A | 31 | 10.50 | 0 | Female | 2 |
| ZN-051 | 1 | O | 34 | 10.50 | 0 | Male | 2 |
| ZN-052 | 2 | A | 50 | 9.90 | 0 | Female | 2 |
| ZN-053 | 2 | A | 35 | 9.80 | 0 | Male | 2 |
| ZN-056 | 2 | A | 24 | 8.67 | 0 | Female | 2 |
| ZN-060 | 2 | A | 51 | 8.23 | 0 | Male | 2 |
| ZN-063 | 1 | O | 36 | 7.03 | 0 | Female | 2 |
| ZN-067 | 2 | A | 54 | 6.87 | 0 | Female | 2 |
| ZN-068 | 2 | A | 49 | 6.77 | 0 | Female | 2 |
| ZN-070 | 2 | A | 33 | 6.17 | 0 | Female | 2 |
| ZN-071 | 1 | O | 36 | 5.87 | 0 | Male | 2 |
| ZN-072 | 1 | A | 67 | 5.83 | 0 | Female | 2 |
| ZN-076 | 1 | O | 31 | 3.30 | 0 | Female | 2 |
| ZN-078 | 2 | A | 72 | 3.10 | 0 | Female | 2 |
| ZN-001 | 2 | O | 61 | 14.60 | 1 | Male | 3 |
| ZN-003 | 1 | O | 56 | 8.80 | 1 | Male | 3 |
| ZN-007 | 2 | OA | 47 | 17.00 | 1 | Male | 3 |
| ZN-011 | 1 | O | 47 | 29.23 | 0 | Female | 3 |
| ZN-012 | 1 | O | 52 | 33.33 | 0 | Male | 3 |
| ZN-013 | 1 | A | 55 | 16.87 | 1 | Male | 3 |
| ZN-015 | 2 | A | 41 | 32.27 | 0 | Female | 3 |
| ZN-019 | 1 | O | 35 | 29.23 | 0 | Male | 3 |
| ZN-020 | 2 | A | 12 | 6.77 | 1 | Female | 3 |
| ZN-022 | 2 | A | 59 | 46.60 | 0 | Female | 3 |
| ZN-024 | 1 | O | 36 | 24.03 | 0 | Male | 3 |
| ZN-026 | 2 | A | 72 | 10.37 | 1 | Male | 3 |
| ZN-029 | 1 | O | 46 | 18.17 | 0 | Female | 3 |
| ZN-031 | 2 | A | 55 | 8.23 | 1 | Male | 3 |
| ZN-035 | 2 | A | 48 | 7.57 | 1 | Female | 3 |
| ZN-038 | 1 | O | 49 | 13.77 | 0 | Male | 3 |
| ZN-041 | 1 | A | 44 | 9.47 | 1 | Female | 3 |
| ZN-042 | 1 | A | 64 | 13.33 | 0 | Male | 3 |
| ZN-044 | 1 | A | 35 | 11.60 | 0 | Male | 3 |
| ZN-046 | 2 | A | 40 | 11.20 | 0 | Female | 3 |
| ZN-049 | 1 | A | 28 | 10.83 | 0 | Female | 3 |
| ZN-054 | 2 | A | 60 | 4.10 | 1 | Male | 3 |
| ZN-057 | 2 | A | 58 | 1.43 | 1 | Male | 3 |
| ZN-058 | 1 | A | 61 | 8.43 | 0 | Female | 3 |
| ZN-059 | 2 | O | 54 | 8.37 | 0 | Male | 3 |
| ZN-061 | 2 | A | 37 | 8.07 | 0 | Female | 3 |
| ZN-062 | 1 | A | 66 | 7.53 | 0 | Female | 3 |
| ZN-064 | 1 | A | 41 | 7.03 | 0 | Female | 3 |
| ZN-065 | 1 | O | 36 | 7.00 | 0 | Male | 3 |
| ZN-066 | 1 | OA | 51 | 6.97 | 0 | Male | 3 |
| ZN-073 | 1 | O | 49 | 5.43 | 0 | Female | 3 |
| ZN-074 | 1 | O | 56 | 5.40 | 0 | Male | 3 |
| ZN-075 | 1 | A | 42 | 3.33 | 0 | Female | 3 |
| **Abbreviations**: *:"A" means "Astrocytoma"; "O" means "Oligodendroglioma"; "OA" means "Oligoastrocytoma" | | | | | | | |

| **Supplementary Table 42**. Metadata of patients in SU-LGG cohort acquired from Liu et al.^45^ | | | | | | | | |
| --- | --- | --- | --- | --- | --- | --- | --- | --- |
| **Patient ID** | **Subtype** | **Age of Diagnosis** | **Gender** | **Grade** | **Histology type*** | **Laterality** | **OS status** | **OS time** |
| 1012026 | 2 | 27 | Male | 2 | O | Right | 0 | 96.57 |
| 1012029 | 1 | 14 | Male | 2 | A | NA | 0 | 68.07 |
| 1012031 | 1 | 33 | Female | 2 | A | Right | 0 | 95.90 |
| 1012033 | 2 | 44 | Male | 2 | A | NA | 0 | 22.40 |
| 1012034 | 1 | 66 | Male | 2 | O | Left | 0 | 88.80 |
| 1012036 | 1 | 48 | Male | 2 | O | Right | 0 | 89.50 |
| 1012042 | 2 | 1 | Male | 2 | A | Left | 0 | 84.13 |
| 1012044 | 2 | 38 | Male | 2 | O | Right | 0 | 34.07 |
| 1012050 | 1 | 39 | Female | 2 | A | Right | 0 | 89.23 |
| 1012051 | 1 | 60 | Female | 2 | O | Right | 0 | 37.47 |
| 1012055 | 1 | 31 | Male | 2 | A | Left | 0 | 84.93 |
| 1012056 | 1 | 54 | Female | 2 | O | Left | 0 | 87.57 |
| 1012058 | 2 | 19 | Male | 2 | A | Left | 0 | 55.03 |
| 1012060 | 2 | 42 | Male | 2 | A | Right | 0 | 13.23 |
| 1012063 | 2 | 53 | Male | 2 | A | NA | 1 | 6.47 |
| 1012073 | 1 | 48 | Female | 2 | O | Right | 0 | 78.40 |
| 1012075 | 2 | 75 | Male | 2 | A | Right | 1 | 7.00 |
| 1012081 | 1 | 74 | Male | 2 | A | Left | 0 | 0.50 |
| 1012086 | 1 | 19 | Male | 2 | O | Left | 0 | 62.27 |
| 1012087 | 2 | 79 | Female | 2 | A | NA | 1 | 10.03 |
| 1012088 | 2 | 23 | Male | 2 | A | Left | 0 | 75.67 |
| 1012092 | 2 | 8 | Male | 2 | A | Left | 0 | 20.50 |
| 1012093 | 2 | 59 | Male | 2 | NA | NA | 1 | 7.63 |
| 1012027 | 2 | 11 | Female | 3 | A | Left | 1 | 8.63 |
| 1012032 | 2 | 77 | Female | 3 | A | Right | 1 | 9.43 |
| 1012040 | 2 | 44 | Male | 3 | A | Left | 1 | 18.77 |
| 1012046 | 1 | 55 | Male | 3 | A | Right | 1 | 12.50 |
| 1012053 | 2 | 83 | Female | 3 | A | NA | 0 | 5.20 |
| 1012059 | 2 | 10 | Female | 3 | A | Right | 1 | 32.47 |
| 1012065 | 1 | 36 | Male | 3 | A | Right | 0 | 83.73 |
| 1012071 | 2 | 41 | Female | 3 | O | Left | 0 | 79.60 |
| 1012074 | 2 | 40 | Male | 3 | A | Right | 1 | 25.60 |
| 1012076 | 1 | 40 | Female | 3 | A | Left | 0 | 74.70 |
| 1012078 | 1 | 67 | Female | 3 | O | Right | 0 | 73.90 |
| 1012079 | 1 | 37 | Female | 3 | A | Left | 1 | 45.97 |
| 1012084 | 2 | 12 | Female | 3 | A | Right | 1 | 7.07 |
| 1012091 | 2 | 75 | Male | 3.00 | A | Right | 1 | 5.8667 |
| **Abbreviations**: *:"A" means "Astrocytoma"; "O" means "Oligodendroglioma"; "OA" means "Oligoastrocytoma" | | | | | | | | |

| **Supplementary Table 43.** PH assumption test on the first proposed nomogram model | | | | |
| --- | --- | --- | --- | --- |
|  | **Characteristic** | **Chi-Square value** | **Degree of freedom** | ***P*-value** |
| **Training set (TCGA-LGG cohort)** | Subtype | 0.91687 | 1 | 0.3383 |
|  | Histological type | 3.2322 | 2 | 0.1987 |
|  | Gender | 0.25572 | 1 | 0.6131 |
|  | Grade | 4.4009 | 1 | 0.0359 |
|  | Global | 6.5378 | 5 | 0.2573 |
| **External validation set 1 (ZN-LGG cohort)** | Subtype | 0.074901 | 1 | 0.7843 |
|  | Histological type | 1.6784 | 2 | 0.4321 |
|  | Gender | 1.0331 | 1 | 0.3094 |
|  | Grade | 3.0669 | 1 | 0.0799 |
|  | Global | 5.4413 | 5 | 0.3644 |
| **External validation set 2 (SU-LGG cohort)** | Subtype | 0.36475 | 1 | 0.5459 |
|  | Histology type | 9.9666e-09 | 1 | 0.9999 |
|  | Gender | 1.5955 | 1 | 0.2065 |
|  | Grade | 5.3612 | 1 | 0.0206 |
|  | Global | 5.5047 | 4 | 0.2393 |
| If the *p*-value in the global test > 0.05, it indicates that the nomogram adheres to the PH assumption. | | | | |

| **Supplementary Table 44.** PH assumption test on the reconstructed nomogram model | | | | |
| --- | --- | --- | --- | --- |
|  | **Characteristic** | **Chi-Square value** | **Degree of freedom** | ***P*-value** |
| **TCGA-LGG cohort** | Subtype | 3.9894 | 1 | 0.0458 |
|  | Gender | 0.19095 | 1 | 0.6621 |
|  | Histological type | 0.044788 | 2 | 0.9779 |
|  | Age | 4.1495 | 1 | 0.0416 |
|  | Laterality | 1.6448 | 2 | 0.4394 |
|  | Grade | 0.024363 | 1 | 0.8760 |
|  | IDH status | 1.2215 | 1 | 0.2691 |
|  | 1p/19q Codeletion | 0.0013788 | 1 | 0.9704 |
|  | MGMT promoter status | 0.56371 | 1 | 0.4528 |
|  | TERT promoter status | 0.038325 | 1 | 0.8448 |
|  | ATRX status | 0.1162 | 1 | 0.7332 |
|  | Primary therapy response | 1.2949 | 1 | 0.2552 |
|  | Follow-up treatment response | 2.8787e-11 | 1 | 1.0000 |
|  | Global | 19.167 | 15 | 0.2063 |
| If the *p*-value in the global test > 0.05, it indicates that the nomogram adheres to the PH assumption. | | | | |
