# Supplementary material for: Limitations of nomogram models in predicting survival outcomes for glioma patients
Source: Front Immunol. 2025 Mar 18;16:1547506. doi: 10.3389/fimmu.2025.1547506 (PMC11959071; doi:10.3389/fimmu.2025.1547506)
Supplement: Supplementary file 2 [file DataSheet2.zip › Data Supplement 2.docx]

###### Data Supplement 2

**Supplementary Figure 1.** Reproduced nomogram model for predicting the survival of glioma patients based on the research conducted by An et al.^18^

**Supplementary Figure 2.** Reproduced nomogram model for predicting the survival of glioma patients based on the research conducted by Chang et al.^19^

**Supplementary Figure 3.** Reproduced nomogram model for predicting the survival of glioma patients based on the research conducted by Yu et al.^20^

**Supplementary Figure 4.** Reproduced nomogram model for predicting the survival of glioma patients based on the research conducted by Jiang et al.^21^

**Supplementary Figure 5.** Reproduced nomogram model for predicting the survival of glioma patients based on the research conducted by Wang et al.^22^

**Supplementary Figure 6.** Reproduced nomogram model for predicting the survival of glioma patients based on the research conducted by Song et al.^23^

**Supplementary Figure 7.** Reproduced nomogram model for predicting the survival of glioma patients based on the research conducted by Chen et al.^25^

**Supplementary Figure 8.** Reproduced nomogram model for predicting the survival of glioma patients based on the research conducted by Han et al.^26^

**Supplementary Figure 9.** Reproduced nomogram model for predicting the survival of glioblastoma (GBM) patients based on the research conducted by Zhang et al.^27^

**Supplementary Figure 10.** Reproduced nomogram model for predicting the survival of glioma patients based on the research conducted by Wang et al.^28^

**Supplementary Figure 11.** Reproduced nomogram model for predicting the survival of glioma patients based on the research conducted by Zhang et al.^29^

**Supplementary Figure 12.** Reproduced nomogram model for predicting the survival of low-grade glioma (LGG) patients based on the research conducted by Ma et al.^30^

**Supplementary Figure 13.** Reproduced nomogram model for predicting the survival of glioma patients based on the research conducted by Ge et al.^31^

**Supplementary Figure 14.** Reproduced nomogram model for predicting the survival of glioma patients based on the research conducted by Peng et al.^32^

**Supplementary Figure 15.** Reproduced nomogram model for predicting the survival of low-grade glioma (LGG) patients based on the research conducted by Zhi et al.^33^

**Supplementary Figure 16.** Reproduced nomogram model for predicting the survival of low-grade glioma (LGG) patients based on the research conducted by Zhang et al.^34^

**Supplementary Figure 17.** Reproduced nomogram model for predicting the survival of low-grade glioma (LGG) patients based on the research conducted by Li et al.^35^

**Supplementary Figure 18.** Reproduced nomogram model for predicting the survival of low-grade glioma (LGG) patients based on the research conducted by Li et al.^36^

**Supplementary Figure 19.** Reproduced nomogram model for predicting the survival of glioma patients based on the research conducted by Wang et al.^37^

**Supplementary Figure 20.** Reproduced nomogram model for predicting the survival of low-grade glioma (LGG) patients based on the research conducted by Zhu et al.^38^

**Supplementary Figure 21.** Reproduced nomogram model for predicting the survival of glioma patients based on the research conducted by Geng et al.^39^

**Supplementary Figure 22.** Reproduced nomogram model for predicting the survival of glioma patients based on the research conducted by Yu et al.^40^

**Supplementary Figure 23.** Reproduced nomogram model for predicting the survival of glioma patients based on the research conducted by Zhou et al.^41^

**Supplementary Figure 24.** Reproduced nomogram model for predicting the survival of glioma patients based on the research conducted by Wu et al.^42^

**Supplementary Figure 25.** Reproduced nomogram model for predicting the survival of glioma patients based on the research conducted by Peng et al.^43^

**Supplementary Figure 26.** Reproduced nomogram model for predicting the survival of glioma patients based on the research conducted by Wang et al.^44^

**Supplementary Figure 27.** Reproduced nomogram model for predicting the survival of glioma patients based on the research conducted by Lin et al.^11^

**Supplementary Figure 28.** Reproduced nomogram model for predicting the survival of glioma patients based on the research conducted by Han et al.^10^

**Supplementary Figure 29.** Reproduced nomogram model for predicting the survival of glioma patients based on the research conducted by Liu et al.^45^

**Supplementary Figure 30.** Reproduced nomogram model for predicting the survival of glioma patients based on the research conducted by He et al.^46^

**Supplementary Figure 31.** Reproduced nomogram model for predicting the survival of glioma patients based on the research conducted by Zeng et al.^24^

**Supplementary Figure 32.** Reproduced nomogram model for predicting the survival of glioma patients based on the research conducted by Zhang et al.^47^

**Supplementary Figure 33.** Reproduced nomogram model for predicting the survival of glioblastoma (GBM) patients based on the research conducted by Zhao et al.^48^

**Supplementary Figure 34.** Reproduced nomogram model for predicting the survival of glioma patients based on the research conducted by Wang et al.^49^

**Supplementary Figure 35.** Reproduced nomogram model for predicting the survival of low-grade glioma (LGG) patients based on the research conducted by Song et al.^17^

**Supplementary Figure 36.** Reproduced nomogram model for predicting the survival of glioma patients based on the research conducted by Dai et al.^50^

**Supplementary Figure 37.** Reproduced nomogram model for predicting the survival of glioblastoma (GBM) patients based on the research conducted by Xie et al.^51^

**Supplementary Figure 38.** Reproduced nomogram model for predicting the survival of glioma patients based on the research conducted by Zeng et al.^14^

**Supplementary Figure 39.** Reproduced nomogram model for predicting the survival of glioma patients based on the research conducted by Wang et al.^52^

**Supplementary Figure 1.** Reproduced nomogram model for predicting the survival of glioma patients based on the research conducted by An et al.^18^ (**A**) Nomogram predicting the 1-year, 3-year, and 5-year survival probabilities of patients. (**B-C**) Calibration plots showing the predicted probabilities of 1-year, 3-year, and 5-year survival in TCGA and CGGA databases compared to actual outcomes.

**Supplementary Figure 2.** Reproduced nomogram model for predicting the survival of glioma patients based on the research conducted by Chang et al.^19^ (**A**) Nomogram predicting the 1-year, 3-year, and 5-year survival probabilities of patients. (**B**) Calibration plots showing the predicted probabilities of 1-year, 3-year, and 5-year survival in CGGA_325 database compared to actual outcomes.

**Supplementary Figure 3.** Reproduced nomogram model for predicting the survival of glioma patients based on the research conducted by Yu et al.^20^ (**A**) Nomogram predicting the 1-year, 3-year, and 5-year survival probabilities of patients. (**B**) Calibration plots showing the predicted probabilities of 1-year, 3-year, and 5-year survival in TCGA database compared to actual outcomes.

**Supplementary Figure 4.** Reproduced nomogram model for predicting the survival of glioma patients based on the research conducted by Jiang et al.^21^ (**A**) Nomogram predicting the 1-year, 3-year, and 5-year survival probabilities of patients. (**B-C**) Calibration plots showing the predicted probabilities of 1-year, 3-year, and 5-year survival in TCGA and CGGA databases compared to actual outcomes.

**Supplementary Figure 5.** Reproduced nomogram model for predicting the survival of glioma patients based on the research conducted by Wang et al.^22^ The nomogram for the prediction of overall survival time was developed using the TCGA database (**A**) and CGGA database (**C**). The calibration plots for predicting 1-, 3-, and 5-year survival using the TCGA database (**B**) and CGGA database (**D**).

**Supplementary Figure 6.** Reproduced nomogram model for predicting the survival of glioma patients based on the research conducted by Song et al.^23^ (**A**) Nomogram predicting the 1-year, 3-year, and 5-year survival probabilities of patients. (**B**) Calibration plots showing the predicted probabilities of 1-year, 3-year, and 5-year survival in TCGA database compared to actual outcomes.

**Supplementary Figure 7.** Reproduced nomogram model for predicting the survival of glioma patients based on the research conducted by Chen et al.^25^ (**A**) Nomogram predicting the 1-year, 3-year, and 5-year survival probabilities of patients. (**B**) Calibration plots showing the predicted probabilities of 1-year, 3-year, and 5-year survival in TCGA database compared to actual outcomes.

**Supplementary Figure 8.** Reproduced nomogram model for predicting the survival of glioma patients based on the research conducted by Han et al.^26^ (**A**) Nomogram predicting the 1-year, 3-year, and 5-year survival probabilities of patients. (**B**) Calibration plots showing the predicted probabilities of 1-year, 3-year, and 5-year survival in CGGA_693 database compared to actual outcomes.

**Supplementary Figure 9.** Reproduced nomogram model for predicting the survival of glioblastoma (GBM) patients based on the research conducted by Zhang et al.^27^ (**A**) Nomogram predicting the 0.5-year, 1-year, and 1.5-year survival probabilities of patients. (**B**) Calibration plots showing the predicted probabilities of 0.5-year, 1-year, and 1.5-year survival in TCGA database compared to actual outcomes.

**Supplementary Figure 10.** Reproduced nomogram model for predicting the survival of glioma patients based on the research conducted by Wang et al.^28^ (**A**) Nomogram predicting the 2-year, 3-year, and 5-year survival probabilities of patients. (**B-E**) Calibration plots showing the predicted probabilities of 2-year, 3-year, and 5-year survival in TCGA, CGGA_301, CGGA_325, and CGGA_693 databases compared to actual outcomes.

**Supplementary Figure 11.** Reproduced nomogram model for predicting the survival of glioma patients based on the research conducted by Zhang et al.^29^ (**A**) Nomogram predicting the 1-year, 3-year, and 5-year survival probabilities of patients. (**B**) Calibration plots showing the predicted probabilities of 1-year, 3-year, and 5-year survival in TCGA database compared to actual outcomes.

**Supplementary Figure 12.** Reproduced nomogram model for predicting the survival of low-grade glioma (LGG) patients based on the research conducted by Ma et al.^30^ The nomogram for the prediction of overall survival time was developed using the TCGA database (**A**) and CGGA database (**C**). The calibration plots for predicting 1-, 3-, and 5-year survival using the TCGA database (**B**) and CGGA database (**D**).

**Supplementary Figure 13.** Reproduced nomogram model for predicting the survival of glioma patients based on the research conducted by Ge et al.^31^ (**A**) Nomogram predicting the 1-year, 3-year, and 5-year survival probabilities of patients. (**B**) Calibration plots showing the predicted probabilities of 1-year, 3-year, and 5-year survival in TCGA database compared to actual outcomes.

**Supplementary Figure 14.** Reproduced nomogram model for predicting the survival of glioma patients based on the research conducted by Peng et al.^32^ (**A**) Nomogram predicting the 1-year, 3-year, and 5-year survival probabilities of patients. (**B**) Calibration plots showing the predicted probabilities of 1-year, 3-year, and 5-year survival in TCGA database compared to actual outcomes.

**Supplementary Figure 15.** Reproduced nomogram model for predicting the survival of low-grade glioma (LGG) patients based on the research conducted by Zhi et al.^33^ (**A, C**) Nomograms were constructed with *PLEKHA4* expression and clinicopathologic variables to estimate 1-, 3- and 5- (**A**) overall survival (OS) and (**C**) disease-specific survival (DSS) of LGG patients. (**B, D**) Calibration plots to verify the accuracy of the predicted 1-, 3- and 5- (**B**) OS and (**D**) DSS of LGG patients in the nomograms. CR, complete response; PR, partial response; SD, stable disease, PD, progressive disease.

**Supplementary Figure 16.** Reproduced nomogram model for predicting the survival of low-grade glioma (LGG) patients based on the research conducted by Zhang et al.^34^ (**A**) Nomogram predicting the 1-year, 3-year, and 5-year survival probabilities of patients. (**B-C**) Calibration plots showing the predicted probabilities of 1-year, 3-year, and 5-year survival in TCGA and CGGA databases compared to actual outcomes.

**Supplementary Figure 17.** Reproduced nomogram model for predicting the survival of low-grade glioma (LGG) patients based on the research conducted by Li et al.^35^ (**A**) Nomogram predicting the 1-year, 3-year, and 5-year survival probabilities of patients. (**B**) Calibration plots showing the predicted probabilities of 1-year, 3-year, and 5-year survival in TCGA database compared to actual outcomes.

**Supplementary Figure 18.** Reproduced nomogram model for predicting the survival of low-grade glioma (LGG) patients based on the research conducted by Li et al.^36^ (**A**) Nomogram predicting the 1-year, 3-year, and 5-year survival probabilities of patients. (**B**) Calibration plots showing the predicted probabilities of 1-year, 3-year, and 5-year survival in TCGA database compared to actual outcomes.

**Supplementary Figure 19.** Reproduced nomogram model for predicting the survival of glioma patients based on the research conducted by Wang et al.^37^ (**A**) Nomogram predicting the 1-year, 3-year, and 5-year survival probabilities of patients. (**B**) Calibration plots showing the predicted probabilities of 1-year, 3-year, and 5-year survival in TCGA database compared to actual outcomes.

**Supplementary Figure 20.** Reproduced nomogram model for predicting the survival of low-grade glioma (LGG) patients based on the research conducted by Zhu et al.^38^ (**A**) Nomogram predicting the 1-year, 3-year, and 5-year survival probabilities of patients. (**B**) Calibration plots showing the predicted probabilities of 1-year, 3-year, and 5-year survival in TCGA database compared to actual outcomes.

**Supplementary Figure 21.** Reproduced nomogram model for predicting the survival of glioma patients based on the research conducted by Geng et al.^39^ (**A, C**) Nomograms were constructed with *SIRPB1* expression and clinicopathologic variables to estimate 1-, 3- and 5- (**A**) overall survival (OS) and (**C**) progression-free interval (PFI) of glioma patients. (**B, D**) Calibration plots to verify the accuracy of the predicted 1-, 3- and 5- (**B**) OS and (**D**) PFI of glioma patients in the nomograms. CR, complete response; PR, partial response; SD, stable disease, PD, progressive disease.

**Supplementary Figure 22.** Reproduced nomogram model for predicting the survival of glioma patients based on the research conducted by Yu et al.^40^ (**A**) Nomogram predicting the 2-year, 3-year, and 5-year survival probabilities of patients. (**B**) Calibration plots showing the predicted probabilities of 2-year, 3-year, and 5-year survival in TCGA database compared to actual outcomes.

**Supplementary Figure 23.** Reproduced nomogram model for predicting the survival of glioma patients based on the research conducted by Zhou et al.^41^ (**A**) Nomogram predicting the 1-year, 3-year, and 5-year survival probabilities of patients. (**B**) Calibration plots showing the predicted probabilities of 1-year, 3-year, and 5-year survival in TCGA database compared to actual outcomes.

**Supplementary Figure 24.** Reproduced nomogram model for predicting the survival of glioma patients based on the research conducted by Wu et al.^42^ (**A**) Nomogram predicting the 1-year, 3-year, and 5-year survival probabilities of patients. (**B**) Calibration plots showing the predicted probabilities of 1-year, 3-year, and 5-year survival in TCGA database compared to actual outcomes.

**Supplementary Figure 25.** Reproduced nomogram model for predicting the survival of glioma patients based on the research conducted by Peng et al.^43^ (**A**) Nomogram predicting the 2-year, 3-year, and 5-year survival probabilities of patients. (**B-D**) Calibration plots showing the predicted probabilities of 2-year, 3-year, and 5-year survival in CGGA_693, TCGA, and CGGA_325 databases compared to actual outcomes.

**Supplementary Figure 26.** Reproduced nomogram model for predicting the survival of glioma patients based on the research conducted by Wang et al.^44^ (**A**) Nomogram predicting the 1-year, 3-year, and 5-year survival probabilities of patients. (**B**) Calibration plots showing the predicted probabilities of 1-year, 3-year, and 5-year survival in TCGA database compared to actual outcomes.

**Supplementary Figure 27.** Reproduced nomogram model for predicting the survival of glioma patients based on the research conducted by Lin et al.^11^ (**A**) Nomogram predicting the 1-year, 3-year, and 5-year survival probabilities of patients. (**B**) Calibration plots showing the predicted probabilities of 1-year, 3-year, and 5-year survival in CGGA database compared to actual outcomes.

**Supplementary Figure 28.** Reproduced nomogram model for predicting the survival of glioma patients based on the research conducted by Han et al.^10^ (**A**) Nomogram predicting the 1-year, 3-year, and 5-year survival probabilities of patients. (**B**) Calibration plots showing the predicted probabilities of 1-year, 3-year, and 5-year survival in CGGA database compared to actual outcomes.

**Supplementary Figure 29.** Reproduced nomogram model for predicting the survival of glioma patients based on the research conducted by Liu et al.^45^ (**A**) Nomogram predicting the 3-year and 5-year survival probabilities of patients. (**B**) Calibration plots showing the predicted probabilities of 3-year and 5-year survival in TCGA database compared to actual outcomes.

**Supplementary Figure 30.** Reproduced nomogram model for predicting the survival of glioma patients based on the research conducted by He et al.^46^ (**A**) Nomogram predicting the 1-year, 3-year, and 5-year survival probabilities of patients. (**B**) Calibration plots showing the predicted probabilities of 1-year, 3-year, and 5-year survival in TCGA database compared to actual outcomes.

**Supplementary Figure 31.** Reproduced nomogram model for predicting the survival of glioma patients based on the research conducted by Zeng et al.^24^ (**A**) Nomogram predicting the 1-year, 3-year, and 5-year survival probabilities of patients. (**B**) Calibration plots showing the predicted probabilities of 1-year, 3-year, and 5-year survival in TCGA database compared to actual outcomes.

**Supplementary Figure 32.** Reproduced nomogram model for predicting the survival of glioma patients based on the research conducted by Zhang et al.^47^ (**A**) Nomogram predicting the 1-year, 3-year, and 5-year survival probabilities of patients. (**B**) Calibration plots showing the predicted probabilities of 1-year, 3-year, and 5-year survival in TCGA database compared to actual outcomes. CR, complete response; PR, partial response; SD, stable disease, PD, progressive disease.

**Supplementary Figure 33.** Reproduced nomogram model for predicting the survival of glioblastoma (GBM) patients based on the research conducted by Zhao et al.^48^ (**A**) Nomogram predicting the 1-year, 1.5-year, and 2-year survival probabilities of patients. (**B**) Calibration plots showing the predicted probabilities of 1-year, 1.5-year, and 2-year survival in TCGA database compared to actual outcomes.

**Supplementary Figure 34.** Reproduced nomogram model for predicting the survival of glioma patients based on the research conducted by Wang et al.^49^ (**A**) Nomogram predicting the 3-year and 5-year survival probabilities of patients. (**B-D**) Calibration plots showing the predicted probabilities of 3-year and 5-year survival in CGGA_301, Gravendeel, and TCGA databases compared to actual outcomes.

**Supplementary Figure 35.** Reproduced nomogram model for predicting the survival of low-grade glioma (LGG) patients based on the research conducted by Song et al.^17^ (**A**) Nomogram predicting the 3-year and 5-year survival probabilities of patients. (**B**) Calibration plots showing the predicted probabilities of 3-year and 5-year survival in TCGA database compared to actual outcomes.

**Supplementary Figure 36.** Reproduced nomogram model for predicting the survival of glioma patients based on the research conducted by Dai et al.^50^ (**A, C, E**) Nomograms were constructed with *PDCD2* expression and clinicopathologic variables to estimate 1-, 3- and 5- (**A**) overall survival (OS), (**C**) disease-specific survival (DSS), and (**E**) progression-free interval (PFI) of glioma patients. (**B, D, F**) Calibration plots to verify the accuracy of the predicted 1-, 3- and 5- (**B**) OS, (**D**) DSS, and (**F**) PFI of glioma patients in the nomograms.

**Supplementary Figure 37.** Reproduced nomogram model for predicting the survival of glioblastoma (GBM) patients based on the research conducted by Xie et al.^51^ (**A**) Nomogram predicting the 1-year and 2-year survival probabilities of patients. (**B**) Calibration plots showing the predicted probabilities of 1-year and 2-year survival in TCGA database compared to actual outcomes.

**Supplementary Figure 38.** Reproduced nomogram model for predicting the survival of glioma patients based on the research conducted by Zeng et al.^14^ (**A**) Nomogram predicting the 1-year, 3-year, and 5-year survival probabilities of patients. (**B**) Calibration plots showing the predicted probabilities of 1-year, 3-year, and 5-year survival in TCGA database compared to actual outcomes.

**Supplementary Figure 39.** Reproduced nomogram model for predicting the survival of glioma patients based on the research conducted by Wang et al.^52^ (**A**) Nomogram predicting the 1-year, 3-year, and 5-year survival probabilities of patients. (**B-C**) Calibration plots showing the predicted probabilities of 1-year, 3-year, and 5-year survival in TCGA and CGGA_325 databases compared to actual outcomes.
